# Global Burden Attributable to High Low-Density Lipoprotein-Cholesterol From 1990 to 2019

**DOI:** 10.3389/fcvm.2022.903126

**Published:** 2022-06-09

**Authors:** Heyue Du, Qingyang Shi, Peige Song, Xiong-Fei Pan, Xueli Yang, Lingmin Chen, Yazhou He, Geng Zong, Ye Zhu, Baihai Su, Sheyu Li

**Affiliations:** ^1^Department of Endocrinology and Metabolism, West China Hospital, Sichuan University, Chengdu, China; ^2^Department of Nephrology, West China Hospital, Sichuan University, Chengdu, China; ^3^Department of Guideline and Rapid Recommendation, Chinese Evidence-Based Medicine Center, Cochrane China Center and MAGIC China Center, West China Hospital, Sichuan University, Chengdu, China; ^4^School of Public Health, Zhejiang University School of Medicine, Zhejiang University, Hangzhou, China; ^5^Division of Epidemiology, Department of Medicine, Vanderbilt University Medical Center, Nashville, TN, United States; ^6^Ministry of Education Key Laboratory of Environment and Health and State Environmental Protection Key Laboratory of Environment and Health, Department of Epidemiology and Biostatistics, School of Public Health, Tongji Medical College, Huazhong University of Science and Technology, Wuhan, China; ^7^The George Institute for Global Health, Faculty of Medicine, University of New South Wales, Sydney, NSW, Australia; ^8^Department of Occupational and Environmental Health, School of Public Health, Tianjin Medical University, Tianjin, China; ^9^Tianjin Key Laboratory of Environment, Nutrition and Public Health, Tianjin Medical University, Tianjin, China; ^10^Center for International Collaborative Research on Environment, Nutrition and Public Health, Tianjin, China; ^11^Department of Anesthesiology and National Clinical Research Center for Geriatrics, West China Hospital, Sichuan University, Chengdu, China; ^12^Research Units of West China, Chinese Academy of Medical Sciences, Chengdu, China; ^13^West China School of Public Health and West China Fourth Hospital, Sichuan University, Chengdu, China; ^14^CAS Key Laboratory of Nutrition, Metabolism and Food Safety, Shanghai Institute of Nutrition and Health, University of Chinese Academy of Sciences, Chinese Academy of Sciences, Shanghai, China; ^15^Department of Cardiology, West China Hospital, Sichuan University, Chengdu, China

**Keywords:** disease burden, high LDL-C, dyslipidemia, ischemic heart disease, stroke

## Abstract

**Background:**

High low-density lipoprotein-cholesterol (LDL-C) is a public health issue contributing to ischemic heart disease (IHD) and stroke.

**Method:**

In this ecological study, we collected summary exposure values (SEVs), deaths, disability-adjusted life of years (DALYs), and Social Demographic Index (SDI) of high LDL-C from 1990 to 2019 using the query tool from the Global Burden of Disease (GBD) Collaborative Network. Outcomes include SEVs, deaths, and DALYs attributable to high LDL-C stratified by sex, age, region, SDI, countries, and territories. Estimated annual percentage changes (EAPCs) were applied to estimate annual trends of changes in these outcomes. We applied the weighted segmented regression with break-point estimation to detect the linear piecewise relationship between SDI and high LDL-C disease burden.

**Results:**

Globally, 3.00 million (95% uncertainty interval [UI], 2.35–3.76 million) people in 1990 and 4.40 million (95% UI, 3.30–5.65 million) people died from high LDL-C in 2019. The absolute annual burden from deaths and DALYs attributed to high LDL-C increased by 46% (95% UI, 35–56%) and 41% (95% UI, 31–50%) from 1990 to 2019. The age-standardized SEV, death, and DALY was decreased by 9% (95% UI, −11 to −8%), 37% (95% UI, −41−33%), and 32% (95% UI, −37 to −28%), respectively, during the study period. There was a negative association between SDI and high LDL-C-related age-standardized death and DALY rates when SDI surpassed 0.71 and 0.71, respectively.

**Conclusion:**

Although the overall age-standardized burden of high LDL-C is controlled in the past 30 years, it remains increasing in moderate SDI countries, and decreasing trends are disappearing in high SDI countries. New challenges require new actions stratified by countries with different SDI levels.

## Introduction

Hyperlipidemia has been well-recognized as a risk factor for cardiovascular diseases. The United Nations Sustainable Development Goals have called for enhanced early warning, risk reduction, and management of non-communicable chronic diseases, including hyperlipidemia, at national and global levels by 2030 (1, 2). Furthermore, the American Heart Association estimated that 93 million US adults, which is approximately accounted for one-third of US residents, have a total cholesterol level exceeding 5.18 mmol/L (200 mg/dl) (3). Dyslipidemia includes increased total cholesterol, low-density lipoprotein cholesterol (LDL-C), non-high-density lipoprotein cholesterol (HDL-C), triglycerides, and low high-density lipoprotein cholesterol (4). Among these, LDL-C contributed as the largest risk factor and most critical therapeutic target to cardiovascular diseases, including ischemic heart disease (IHD) and ischemic stroke (5–7). For IHD, many patients may experience reduced working activities, lose productivity, and some experience difficulties returning to premorbid activities. Stroke is ranked the second most common cause of death and the third most common cause of disability, with complications, such as pneumonia, deep vein thrombosis, gastric bleeding, mood disorders, and falls (8).

For decades, policymakers and clinical scientists made every effort to control high LDL-C and its complications, especially in high-income countries (9, 10). However, it is unclear whether the latest public health and medical progressions have translated into a reduced disease burden of high LDL-C or the shift of its epicenter. This analysis summarizes the trends in LDL-C-related burden across age, sex, regions, and socio-demographic index (SDI) by retrieving Global Burden of Disease (GBD) data and utilizing advanced statistical methods. The research is aimed to provide healthcare stakeholders with updated LDL-C-attributed disease burdens for 204 countries and territories and inform public health policy decision-making.

## Materials and Methods

### Data Source

We obtained the summary exposure values (SEVs), deaths, and disability-adjusted life of years (DALYs) related to the high LDL-C risk factor from 1990 to 2019 from the latest release of the GBD Collaborative Network^1^ by 16 March 2021 (11). The GBD collaborators have described the detailed comprehensive methodological risk assessment of high LDL-C elsewhere (12). Moreover, informed consent for accessing the GBD data was waived by the University of Washington Institutional Review Board (12). This study followed the Guidelines for Accurate and Transparent Health Estimates Reporting (GATHER), and the checklist is attached (Supplementary Checklist) (13).

### Definitions

High LDL-C is defined as a blood LDL-C concentration that surpasses the theoretical minimum risk exposure level, which is 1.3 mmol/L (50 mg/dl), as determined by a previous study (12). The GBD team accessed 711 input data from 145 countries/territories with available data. The previous publications described the approaches for data collection and metrics estimation (14). We adopted the definitions from the GBD team. SEV summarizes populational exposure distribution to the high LDL-C risk factor. DALY means the total number of years lost due to high LDL-C. Moreover, the detailed definitions of SEV, DALY, SDI, and age stratifications are attached in Supplementary Table 1.

### Statistical Methods

Estimated annual percentage changes (EAPCs) with 95% confidence intervals (CIs) were applied to estimate annual trends of changes in age-standardized SEV, death, and DALY rates (detailed EAPC and DALY definition is in Supplementary Table 7). We applied the weighted segmented regression with break-point estimation to detect the linear piecewise relationship between the 2019 SDI value and LDL-C-attributable disease burden (i.e., EAPCs of age-standardized death and DALY rates) with inverse-variance weighting according to the uncertainty of EAPCs. We assessed the linear correlations for two separate lines in the segmented regressions by weighted Pearson’s product-moment method. A sensitivity analysis was performed to test the robustness of the relationships between SDI level and EAPCs by 1990, 2000, and 2010 SDI values. We drew the scatter plots to display the relationship between the SDI and age-standardized rate (ASR) of death and DALY for 30 years. Results with a *p*-value < 0.05 with a 95% CI excluding 0 were considered statistically significant. All statistical processes were performed by R software (version 4.0.3).

## Results

### The Overall Impact of High Low-Density Lipoprotein-Cholesterol

**FIGURE 1:**
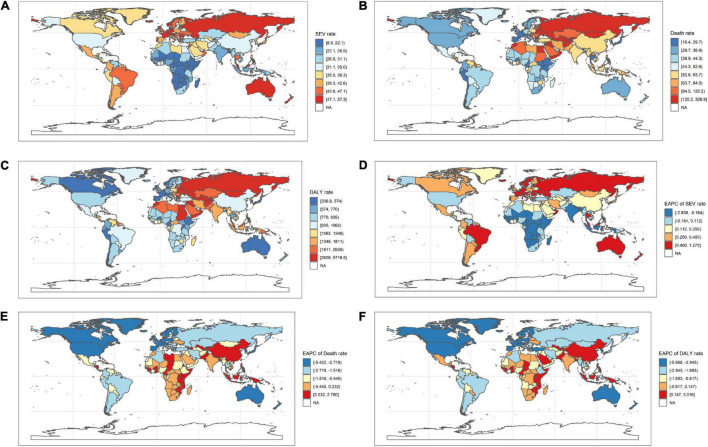
The ASR of high LDL-C attributable death and DALY in 2019, and the EAPC of age-standardized death and DALY rate from 1990 to 2019 in 204 countries and territories. **(A)** ASR of high LDL-C attributable SEV in 2019; **(B)** ASR of high LDL-C attributable death in 2019; **(C)** ASR of high LDL-C attributable to DALY in 2019; **(D)** EAPC of age-standardized SEV rate from 1990 to 2019. **(E)** EAPC of age-standardized death rate from 1990 to 2019; **(F)** EAPC of age-standardized DALY rate from 1990 to 2019. DALY, disability-adjusted life year; SDI, social-demographic index; EAPC, estimated annual percentage change; SEV, summary exposure value; ASR, age-standardized rate; LDL-C, low-density lipoprotein-cholesterol.

Global age-standardized SEV rates attributable to high LDL-C have decreased by 9.1% from 35.7 (95% UI 32.9–38.7) in 1990 to 32.4 (95% UI 29.5–35.6) in 2019. While, global deaths attributable to high LDL-C have increased 0.4-fold from 3.0 million (95% UI 2.4–3.8 million) in 1990 to 4.40 million (95% UI 3.3–5.7 million) in 2019. Global DALYs attributable to high LDL-C have also increased from 69.7 million (95% UI 58.5–83.3 million) in 1990 to 98.6 million (95% UI 80.3–119.0 million) in 2019. In addition, the ASRs for death were 89.3 per 100,000 people (95% UI 67.1–115.6 per 100,000 people) in 1990 and 56.5 per 100,000 people (95% UI 41.8–73.6 per 100,000 people) in 2019 and those for DALYs were 1779.9 per 100,000 people (95% UI 1465.1–2154.3 per 100,000 people) in 1990 and 1207.2 per 100,000 people (95% UI 975.1–1461.1 per 100,000 people) in 2019. Meanwhile, the age-standardized SEV rates were 35.7 per 100,000 people (95% UI 32.9–38.7 per 100,000 people) in 1990 and 32.4 per 100,000 people (95% UI 29.5–35.6 per 100,000 people) in 2019. Nevertheless, the ASRs between 1990 and 2019 of high LDL-C-related SEVs, deaths, and DALYs were decreased by 9.1% (95% UI −10.5% to −7.9%), 36.7% (95% UI −40.6% to −33.1%), and 32.2% (95% UI −36.7% to −27.8%), respectively (Tables 1–3 and Figures 1A–F).

**TABLE 1:** Trends of ASR SEV attributable to high LDL-C in 1990 and 2019.

Characteristics	1990 age-standardized SEV rate per 100,000 people No. (95%UI)	2019 age-standardized SEV rate per 100,000 people No. (95%UI)	1990-2019 EAPC%. (95%UI)	percentage change in age-standardized SEV rates, 1990-2019
Overall	35.7 (32.9, 38.7)	32.4 (29.5, 35.6)	–0.3% (–0.4%, –0.2%)	–9.1% (–10.5%, –7.9%)
**sex**				
Female	37 (34.3, 40.1)	33.7 (30.8, 36.9)	–0.3% (–0.3%, –0.3%)	–9% (–10.4%, –7.8%)
Male	34.1 (31.4, 37.2)	31.1 (28.1, 34.3)	–0.3% (–0.4%, –0.3%)	–9% (–10.5%, –7.7%)
**Socio-demographic index**				
High SDI	54 (51.8, 56.5)	39.5 (36.6, 42.6)	0.1% (0%, 0.1%)	–26.9% (–29.5%, –24.4%)
High-middle SDI	39 (36.3, 42.1)	37.8 (34.9, 41)	–1.1% (–1.2%, –1%)	–3.2% (–4.3%, –2.1%)
Middle SDI	29.9 (26.8, 33.2)	32.2 (29.2, 35.5)	0.2% (0.1%, 0.3%)	7.7% (6.5%, 9.1%)
Low-middle SDI	23.9 (20.9, 27.2)	25.8 (22.8, 29)	0.3% (0.2%, 0.5%)	7.9% (6.5%, 9.5%)
Low SDI	19.8 (16.8, 23)	21.8 (18.8, 25.1)	0.2% (0.1%, 0.4%)	10.3% (8.5%, 12.4%)
**Geographic regions**				
Andean Latin America	31.1 (28, 34.5)	34.3 (31.2, 37.7)	0.4% (0.3%, 0.5%)	10.2% (7.1%, 13.9%)
Australasia	50 (47.2, 52.8)	48.6 (45.6, 51.5)	0% (–0.1%, 0%)	–2.7% (–6.2%, 0.7%)
Caribbean	30.9 (27.9, 34.2)	32.5 (29.4, 35.7)	0.2% (0.1%, 0.3%)	5% (3%, 7%)
Central Asia	31.1 (28, 34.4)	32.9 (29.9, 36.2)	0.1% (0.1%, 0.2%)	5.6% (3.7%, 7.8%)
Central Europe	48.5 (45.8, 51.4)	44.1 (41.2, 47.1)	–0.4% (–0.4%, –0.4%)	–9% (–10.7%, –7.2%)
Central Latin America	34.5 (31.4, 37.7)	38.4 (35.4, 41.4)	0.4% (0.3%, 0.5%)	11.3% (8.5%, 14.6%)
Central Sub-Saharan Africa	21.2 (18.1, 24.5)	21.8 (18.8, 25.3)	0% (–0.1%, 0.2%)	2.9% (0%, 6%)
East Asia	29.2 (26.1, 32.5)	32.3 (29.3, 35.6)	0.4% (0.3%, 0.4%)	10.5% (9%, 12.5%)
Eastern Europe	48 (45.4, 50.9)	47.3 (44.4, 50.2)	–0.1% (–0.1%, –0.1%)	–1.6% (–4.7%, 1.5%)
Eastern Sub-Saharan Africa	16.8 (13.9, 19.9)	19 (16.1, 22.2)	0.4% (0.1%, 0.6%)	13.1% (10.7%, 16.4%)
High-income Asia Pacific	37.3 (34.4, 40.4)	39.5 (36.6, 42.7)	–2.6% (–2.8%, –2.4%)	5.9% (2.8%, 9%)
High-income North America	64.7 (62.5, 67)	31.9 (28.8, 35.2)	–0.1% (–0.2%, –0.1%)	–50.7% (–54.7%, –46.7%)
North Africa and Middle East	33.1 (30.1, 36.4)	35 (32, 38.2)	0.2% (0.1%, 0.3%)	5.5% (4.3%, 7.1%)
Oceania	29 (25.9, 32.4)	30 (26.9, 33.4)	0% (–0.1%, 0.1%)	3.3% (0.6%, 6.5%)
South Asia	22 (19, 25.3)	23.8 (20.7, 27)	0.2% (0.1%, 0.4%)	7.9% (6.5%, 9.7%)
Southeast Asia	30.3 (27.3, 33.5)	32.1 (29.1, 35.3)	0.2% (0%, 0.3%)	5.8% (4.3%, 7.7%)
Southern Latin America	37.1 (33.9, 40.5)	41.3 (38.3, 44.4)	0.4% (0.3%, 0.4%)	11.2% (7.7%, 14.8%)
Southern Sub-Saharan Africa	23.3 (20.2, 26.6)	25.7 (22.6, 28.9)	0.4% (0.2%, 0.5%)	10.3% (6.6%, 14.6%)
Tropical Latin America	40.3 (36.9, 43.7)	45 (41.8, 48.2)	0.4% (0.4%, 0.4%)	11.8% (7%, 17.1%)
Western Europe	54.3 (51.9, 56.7)	45.6 (42.9, 48.5)	–0.8% (–0.8%, –0.7%)	–16% (–17.8%, –14.2%)
Western Sub-Saharan Africa	21.9 (19, 25.1)	23.4 (20.5, 26.7)	0.2% (0.1%, 0.3%)	6.7% (4.1%, 9.6%)

*DALY, disability-adjusted life year; ASR, age-standardized rate; UI, uncertainty interval; CI, confidence interval; EAPC, estimated annual percentage change; SEV, summary exposure value; LDL-C, low-density lipoprotein-cholesterol.*

**TABLE 2:** Trends of deaths and corresponding ASR attributable to high LDL-C in 1990 and 2019.

Characteristics	Death in 1990, *n* (95%UI)	Age-standardized death rate per 100,000 in 1990, *n* (95%UI)	Death in 2019, *n* (95%UI)	Age-standardized death rate per 100,000 in 2019, *n* (95%UI)	EAPC of death from 1990 to 2019,% (95%CI)	EAPC of age-standardized death rates from 1990 to 2019, *n* (95%CI)
Overall	3002610.9 (2350832.4, 3761875.1)	89.3 (67.1, 115.6)	4396983.3 (3301257.8, 5651785.3)	56.5 (41.8, 73.6)	–1.7% (–1.8%, –1.6%)	–36.7% (–40.6%, –33.1%)
**Sex**						
Female	1429806.6 (1066417.3, 1869598.3)	76.2 (55.2, 102)	2037019.9 (1430378.7, 2735095.4)	46.5 (32.7, 62.4)	–1.9% (–1.9%, –1.8%)	–38.9% (–43.7%, –34.6%)
Male	1572804.3 (1270174.6, 1913519)	103.6 (79.5, 131.9)	2359963.4 (1831942.7, 2955417.3)	67.3 (50.8, 86.4)	–1.6% (–1.6%, –1.5%)	–35% (–39.6%, –30.5%)
**Socio-demographic index**						
High SDI	965808 (726808.3, 1233486.8)	93.9 (70.9, 119.7)	692928.3 (483134.6, 942793.5)	32.9 (24, 43.5)	–3.8% (–4.1%, –3.6%)	–64.9% (–67.1%, –62.8%)
High-middle SDI	1048885 (816364.8, 1328195.3)	115.8 (86.9, 151.9)	1387922.1 (1023850.4, 1829040)	70.7 (51.8, 93.7)	–2% (–2.2%, –1.8%)	–39% (–42.8%, –35.5%)
Middle SDI	563865.3 (450489.3, 708879.4)	66.5 (49.8, 87.2)	1354977.9 (1035814.4, 1729138.1)	62.6 (45.5, 82.5)	–0.1% (–0.2%, 0%)	–5.9% (–15%, 2.2%)
Low-middle SDI	315039.5 (250427, 394004)	60.4 (45.4, 79.1)	725430.7 (556524.7, 915367.9)	58.4 (43.1, 76.1)	–0.1% (–0.1%, 0%)	–3.3% (–13.7%, 6.1%)
Low SDI	107449.5 (82914.5, 138543.8)	51.9 (37.9, 70.5)	233277.2 (177952.9, 295556.1)	49.9 (36, 66.4)	–0.2% (–0.2%, –0.1%)	–3.9% (–16.1%, 6.9%)
**Geographic regions**						
Andean Latin America	8878.1 (6776.6, 11530.8)	47.9 (35.1, 64.1)	15885.4 (11266.8, 21610.2)	29.4 (20.5, 40.2)	–1.6% (–1.8%, –1.4%)	–38.6% (–50.8%, –24.9%)
Australasia	21257 (15991.2, 26994.5)	96 (71.4, 122.8)	17343.4 (11724.4, 23585.6)	31.4 (22.1, 42)	–4.2% (–4.4%, –3.9%)	–67.3% (–70.4%, –64.5%)
Caribbean	19616.4 (14957.2, 24867.3)	81.7 (61, 105.6)	29568.9 (21427.1, 38714.5)	56.9 (41.2, 74.5)	–1.3% (–1.5%, –1%)	–30.4% (–39%, –21.1%)
Central Asia	60238.7 (46859.3, 75028)	141.7 (105.8, 182)	93873.2 (72198.4, 118455.1)	155.8 (111.2, 206.2)	–0.1% (–0.5%, 0.4%)	10% (1.1%, 19.2%)
Central Europe	220061.4 (169872.8, 276861.7)	168.2 (126.8, 215.3)	187517.6 (129876.4, 255862.8)	86 (60.3, 116.3)	–2.7% (–2.9%, –2.6%)	–48.9% (–55.1%, –43%)
Central Latin America	44054.2 (34471.8, 54584.3)	59.9 (44.4, 77)	103225.3 (75849.5, 133950.8)	45.3 (32.9, 59.6)	–1.1% (–1.2%, –0.9%)	–24.4% (–33.3%, –13.7%)
Central Sub-Saharan Africa	9302.9 (6700.4, 12569.1)	49.5 (33.9, 69)	20068.9 (13639.1, 27863.6)	45.3 (28.9, 66.9)	–0.4% (–0.5%, –0.4%)	–8.6% (–26.3%, 13.2%)
East Asia	330873.8 (255640.7, 431857.2)	49 (35.3, 68.3)	945415.7 (670742, 1278097)	55.1 (37.3, 76.3)	0.9% (0.7%, 1.1%)	12.3% (–5.6%, 32.3%)
Eastern Europe	470672.1 (363126.3, 600428)	191 (144.7, 248.4)	542562.6 (401965.6, 704241.3)	157.4 (117, 203.2)	–1.2% (–1.7%, –0.8%)	–17.6% (–24.9%, –9.7%)
Eastern Sub-Saharan Africa	20198.4 (14778.3, 27146.9)	32.2 (22.2, 45.9)	45501.6 (31509.7, 62131.1)	33.2 (21.4, 47.4)	0.1% (0%, 0.1%)	3.2% (–20%, 24.5%)
High-income Asia Pacific	81885.9 (59999, 111931.8)	47.3 (33.1, 66.6)	93396.4 (59679, 140617)	16.7 (11.6, 23.8)	–3.6% (–3.8%, –3.5%)	–64.6% (–67.9%, –61.9%)
High-income North America	378383.7 (286525.9, 476114.7)	105.8 (81.4, 131.7)	248784.4 (174969.8, 335812.4)	37.1 (27.1, 49)	–3.8% (–4.1%, –3.6%)	–64.9% (–68%, –61.9%)
North Africa and Middle East	213915.8 (172149.4, 259172.8)	140.5 (107.6, 178.2)	401216.8 (309136.4, 506065.5)	103.3 (75.3, 135.6)	–1.2% (–1.2%, –1.1%)	–26.5% (–35.2%, –18.6%)
Oceania	2442.4 (1870.2, 3199)	84.5 (63.6, 112.6)	6332.9 (4820.4, 8483.4)	91.1 (67.2, 123.5)	0.3% (0.2%, 0.3%)	7.7% (–9.6%, 30%)
South Asia	321684.1 (255937.9, 402587)	62.7 (47.5, 83.1)	778372.3 (596988.5, 982064.4)	59.9 (44.5, 77.7)	–0.2% (–0.3%, –0.1%)	–4.4% (–19.4%, 9.4%)
Southeast Asia	124397.4 (97636.4, 158909.1)	56.2 (42.1, 75.2)	299162.7 (229075.4, 378090.2)	55.2 (40.2, 73)	0% (–0.1%, 0.1%)	–1.8% (–14%, 9.4%)
Southern Latin America	32278.4 (24533.7, 41118.5)	78 (58, 101.2)	31494.7 (23237.3, 41310.5)	37.2 (27.8, 48.5)	–2.5% (–2.7%, –2.3%)	–52.2% (–54.8%, –49.5%)
Southern Sub-Saharan Africa	9602.4 (7222.3, 12604.2)	38.5 (27.9, 52.9)	20005.3 (14891.5, 26258.8)	41.6 (29.5, 57.5)	0.4% (–0.1%, 0.8%)	7.9% (–2.5%, 18.6%)
Tropical Latin America	69531.3 (56053, 85327.8)	87.7 (67.1, 113.7)	101999.6 (80130.1, 129224.8)	43.3 (33.5, 56)	–2.3% (–2.4%, –2.2%)	–50.6% (–53.2%, –48%)
Western Europe	529815.2 (393312.7, 689930.1)	91.8 (69.2, 118.8)	348488 (235099.8, 486254.3)	32.2 (23, 43.4)	–4% (–4.2%, –3.7%)	–64.9% (–67.5%, –62.3%)
Western Sub-Saharan Africa	33521.2 (23253.2, 46745.4)	46.5 (30.9, 67.1)	66767.8 (47389.9, 88975.1)	43 (29.1, 59.4)	–0.3% (–0.4%, –0.2%)	–7.5% (–30%, 10.9%)

*ASR, age-standardized rate; UI, uncertainty interval; EAPC, estimated annual percentage change; CI, confidence interval; LDL-C, low-density lipoprotein-cholesterol.*

**TABLE 3:** Trends of DALYs and corresponding ASR attributable to high LDL-C in 1990 and 2019.

Characteristics	DALY in 1990, *n* (95%UI)	Age-standardized DALY rate per 100,000 in 1990, *n* (95%UI)	DALY in 2019, *n* (95%UI)	Age-standardized DALY rate per 100,000 in 2019, *n* (95%UI)	EAPC of DALY from 1990 to 2019,% (95%CI)	EAPC of age-standardized DALY rates from 1990 to 2019, % (95% CI)
Overall	69718898.1 (58516399.6, 83278990.5)	1779.9 (1465.1, 2154.3)	98618020.8 (80335172.4, 118987709.3)	1207.2 (975.1, 1461.1)	–1.5% (–1.5%, –1.4%)	–32.2% (–36.7%, –27.8%)
**Sex**						
Female	28291096.8 (23140593.7, 35000010)	1368.2 (1103.4, 1703.6)	39004193.7 (30595475, 48738967.5)	898.3 (706, 1120.4)	–1.6% (–1.7%, –1.6%)	–32.4% (–39.8%, –29%)
Male	41427801.2 (35215234.8, 48297678.1)	2207.7 (1825.4, 2639.9)	59613827 (49348638.5, 71183124.8)	1528.7 (1250.3, 1833.4)	–1.4% (–1.4%, –1.3%)	–30.8% (–36.3%, –25.2%)
**Socio-demographic index**						
High SDI	18098188.5 (14880515, 21624697.5)	1777.7 (1475.5, 2109.5)	11836269.6 (9371712.3, 14751584)	673.6 (551.6, 813.6)	–3.5% (–3.8%, –3.3%)	–62.1% (–64.1%, –59.9%)
High-middle SDI	23350467.3 (19585604.3, 27876658.3)	2249 (1860.7, 2742.3)	27375113.4 (22092050.3, 33387173.7)	1372.1 (1107.4, 1671.7)	–2.1% (–2.4%, –1.9%)	–39% (–43.1%, –35.1%)
Middle SDI	15653910.6 (13178411.2, 18667615)	1451.8 (1183.6, 1785)	32890593.9 (26876976.9, 39383673.9)	1318 (1060.4, 1606.7)	–0.2% (–0.3%, –0.2%)	–9.2% (–17.9%, –1.1%)
Low-middle SDI	9372874.4 (7696133.5, 11270657.4)	1426 (1150.3, 1759.6)	19679526.6 (16047461.3, 23815769.8)	1367.5 (1088.8, 1676.5)	–0.1% (–0.1%, 0%)	–4.1% (–15%, 6.6%)
Low SDI	3206519.4 (2550203.6, 4002083.7)	1231.4 (959, 1564.7)	6777758.8 (5396659.4, 8336559.5)	1166.2 (909, 1456)	–0.2% (–0.3%, –0.2%)	–5.3% (–16.7%, 6.2%)
**Geographic regions**						
Andean Latin America	214986.6 (176154.8, 259842)	990.4 (791.3, 1226.2)	338055.1 (257785.8, 436204.5)	592.6 (448.6, 768.5)	–1.7% (–2%, –1.5%)	–40.2% (–52.1%, –26.1%)
Australasia	410350.7 (336783.2, 490764.5)	1802.7 (1486.7, 2147.1)	263625.2 (203847.3, 330399.4)	550 (441.6, 669.4)	–4.3% (–4.6%, –4%)	–69.5% (–71.4%, –67.3%)
Caribbean	455640.5 (374637.1, 544269.6)	1740 (1415.5, 2093.8)	655675.4 (510901.3, 817921.3)	1268.5 (989, 1582.9)	–1.1% (–1.3%, –0.9%)	–27.1% (–37.6%, –16.1%)
Central Asia	1402715.2 (1168262.6, 1660512.6)	2958.8 (2409.9, 3553.8)	2285623.4 (1861650.7, 2772349.5)	3067.4 (2409.3, 3821.8)	–0.4% (–0.8%, 0.1%)	3.7% (–5.6%, 14.6%)
Central Europe	4650950 (3904057.2, 5496381.6)	3275.1 (2728.9, 3895.7)	3168229 (2387448.3, 4062823.7)	1549.6 (1199.8, 1947.4)	–3% (–3.2%, –2.9%)	–52.7% (–58.7%, –46.9%)
Central Latin America	1087162.1 (916899.4, 1277380)	1235.7 (1012.7, 1482.5)	2216093.8 (1761184.4, 2717539.2)	924.1 (727, 1141.8)	–1.1% (–1.3%, –0.9%)	–25.2% (–34.6%, –13.8%)
Central Sub-Saharan Africa	273853.1 (203048.3, 363785)	1122.5 (814.7, 1507.9)	567789.2 (400014.4, 774693.9)	976.6 (675.2, 1350.1)	–0.6% (–0.6%, –0.5%)	–13% (–31.4%, 9.9%)
East Asia	9264760.1 (7479533.2, 11506939.6)	1050.7 (831.8, 1349.4)	20511633.7 (15776519.1, 25975823.2)	1048.1 (801.9, 1338.9)	0.4% (0.2%, 0.5%)	–0.2% (–16.2%, 18.8%)
Eastern Europe	9878553.9 (8238415.9, 11819198.9)	3674 (3038.6, 4433)	10286201.8 (8116438, 12600339.5)	3079.1 (2456.4, 3744.3)	–1.3% (–1.9%, –0.7%)	–16.2% (–24.4%, –6.7%)
Eastern Sub-Saharan Africa	595033.3 (454677.4, 775496.6)	736.3 (546.3, 976.2)	1280317.2 (940318.7, 1665083.3)	718 (511.8, 955.1)	–0.2% (–0.2%, –0.1%)	–2.5% (–25.1%, 19.6%)
High-income Asia Pacific	1674595.9 (1352932.6, 2098587.9)	865.6 (689.5, 1111.9)	1431401.3 (1041808.3, 1969403.1)	343.2 (273.8, 434.2)	–3.2% (–3.3%, –3.1%)	–60.4% (–62.4%, –57.8%)
High-income North America	7085690.9 (5855628.9, 8332418.2)	2087.8 (1762.9, 2419.9)	4542008.8 (3601870, 5575724.4)	780.3 (636.3, 938.8)	–3.5% (–3.8%, –3.3%)	–62.6% (–65.2%, –60.3%)
North Africa and Middle East	5917147.9 (4939964.8, 6959645.8)	3201.1 (2628.8, 3822.8)	10449397.8 (8403498.4, 12688546.4)	2235.4 (1767.8, 2767.8)	–1.4% (–1.5%, –1.4%)	–30.2% (–39%, –20.9%)
Oceania	81860.4 (63537.2, 106052.7)	2273.2 (1758.2, 2950.4)	209075.7 (160857.2, 272633)	2396.1 (1837.2, 3185.8)	0.2% (0.2%, 0.3%)	5.4% (–13%, 29.5%)
South Asia	10035748.5 (8235497.6, 12179541.2)	1541.5 (1234, 1911.5)	22064652.2 (17543323.4, 27071516)	1466.4 (1154.4, 1816.8)	–0.1% (–0.2%, 0%)	–4.9% (–20%, 9.7%)
Southeast Asia	3558981.1 (2942496.1, 4332432.8)	1282.3 (1029.8, 1606.5)	7994791.1 (6469368.9, 9703182.6)	1249.2 (996.3, 1543.5)	0% (–0.1%, 0.1%)	–2.6% (–14.4%, 9.7%)
Southern Latin America	678354 (561360.3, 812908.7)	1510.5 (1236.6, 1819.5)	598356.2 (490744.3, 726766.9)	733.4 (606.9, 885.5)	–2.5% (–2.6%, –2.3%)	–51.5% (–54%, –48.9%)
Southern Sub-Saharan Africa	266949.9 (215194.6, 331269)	898.5 (705, 1143.4)	500743.9 (399020.4, 616114.6)	865 (666.5, 1092.1)	0% (–0.5%, 0.4%)	–3.7% (–13.4%, 5.8%)
Tropical Latin America	1849692.2 (1572583.1, 2174450)	1921.8 (1599.2, 2306.4)	2422997 (2040965.2, 2853889.2)	982.6 (819.5, 1163.9)	–2.2% (–2.3%, –2.1%)	–48.9% (–51.4%, –46.3%)
Western Europe	9471373.2 (7685248.2, 11548884.2)	1697.3 (1408.6, 2037.3)	5083993.8 (3863904.5, 6500930.5)	572.5 (462.4, 695.2)	–4.1% (–4.3%, –3.8%)	–66.3% (–67.9%, –64.7%)
Western Sub-Saharan Africa	864498.5 (629642.6, 1178057.5)	965.5 (688, 1330.3)	1747359.1 (1302306.3, 2260966.3)	875.8 (637, 1151)	–0.4% (–0.5%, –0.3%)	–9.3% (–30.1%, 10%)

*DALY, disability-adjusted life year; ASR, age-standardized rate; UI, uncertainty interval; EAPC, estimated annual percentage change; CI, confidence interval; LDL-C, low-density lipoprotein-cholesterol.*

Among the GBD level 2 risk factors, high LDL-C ranked 8th and 9th in age-standardized DALY rate in 1990 and 2019 in all SDI regions. Similarly, the high LDL-C attributable age-standardized death rate was listed 6th in 1990 and 7th in 2019. For high SDI regions, the ranks showed decreasing trends in both age-standardized death and DALY rates. For high-middle to low SDI regions, high LDL-C attributable age-standardized death and DALY rate ranks did not slide down significantly over the 30 years (Supplementary Tables 2, 3 and Supplementary Figure 1).

Age-specific high LDL-C attributable death and DALY rates were increased with aging. Death associated with high LDL-C was highest for both sexes in the age group 80–84 years. The death numbers showed two peaks in the 60–64 and 80–84 years age group for men. The DALY peaks in the 55–59 age group in men, with two peaks among women (60–64 and 80–84 years old age groups). Men’s death and DALY numbers were higher than women before 80 years old, and women surpassed men in deaths and DALYs when age groups > 80 years. The analysis showed age-standardized death and DALY rate EAPCs’ decreasing trends for men (death −1.56, 95% CI −1.61 to −1.51; DALY −1.36, 95% CI −1.40 to −1.31) and women (death −1.86, 95% CI −1.93 to −1.79; DALY −1.61, 95% CI −1.68 to −1.55; Supplementary Figures 2a–l).

At the regional geographic level, the highest age-standardized SEV rates attributable to high LDL-C in 2019 were Australasia (48.6 per 100,000 people, 95% UI 45.6–51.5 per 100,000 people), followed by Eastern Europe (47.3 per 100,000 people, 95% UI 44.4–50.2 per 100,000 people) and Western Europe (45.6 per 100,000 people, 95% UI 42.9–48.5 per 100,000 people). The age-standardized SEV rates were decreased most in high-income North America (−50.7%, 95% UI −54.7 to −46.7%), followed by Western Europe (−16%, 95% UI −17.8 to −14.2%), and Central Europe (−9%, 95% UI −10.7 to −7.2%; Table 1). The highest number of DALYs attributable to high LDL-C in 2019 were occurred in South Asia (22.1 million, 95% UI 17.5–27.1 million), followed by East Asia (DALY 20.5 million, 95% UI 15.8–26.0 million), and North Africa and Middle East (DALY 10.5 million, 95% UI 8.4–12.7 million; Table 3). The three geographic regions with the highest death numbers in 2019 were East Asia (0.9 million, 95% UI 0.7–1.3 million), South Asia (0.8 million, 95% UI 0.6–1.0 million), and Eastern Europe (0.5 million, 95% UI 0.4–0.7 million; Table 2). The age-standardized death and DALY rates were decreased most in Australasia (death −67.3%, 95% UI −70.4 to −64.5%; DALY −69.5%, 95% UI −71.4 to −67.3%), followed by Western Europe (death −64.9%, 95% UI −67.5 to −62.3%; DALY −66.3%, 95% UI −67.9 to −64.7%), and High-income North America (death −64.9%, 95% UI −68.0 to −61.9%; DALY −62.7%, 95% UI −65.2 to −60.3%) from 1990 to 2019 (Tables 2, 3). At the national level, the top three countries/territories with different scales were listed (Supplementary Tables 4–6).

### The Burden of High Low-Density Lipoprotein-Cholesterol Attributable to Ischemic Stroke and Ischemic Heart Disease

In 2019, IHD and ischemic stroke were the major diseases attributable to high LDL-C, accounting for 86.1 and 13.9% of high LDL-C attributed death numbers. A similar pattern was observed in DALYs. High LDL-C-related age-standardized death and DALY rates in 2019 were found for IHD (death 48.4, 95% UI 35.5–63.0 per 100,000 people; DALY 1036.9, 95% UI 843.8–1245.0 per 100,000 people) and ischemic stroke (death 8.7, 95% UI 3.2–18.2 per 100,000 people; DALY 170.2, 95% UI 92.8–294.7 per 100,000 people). The proportions of age-standardized death and DALY rates for ischemic stroke and IHD attributed to high LDL-C were consistent with the general LDL-C-related disease burden (Supplementary Tables 7, 8).

### Relationship Between Social Demographic Index and High Low-Density Lipoprotein-Cholesterol Attributable Disease Burden

As reported in Tables 2, 3, high LDL-C-related death and DALY numbers were increased in all SDI levels, except in high SDI regions. Furthermore, age-standardized death and DALY rates were decreased at all SDI levels. However, age-standardized SEV rates were only decreased in high and high-middle SDI regions, with increasing trends in low and middle SDI regions. From 1990 to 2019, the highest number of deaths and DALYs were seen in the high-middle SDI quintile (death 1.1 million, 95% UI 0.8–1.3 million; DALY 23.4 million, 95% UI 19.6–27.9 million) in 1990 and (1.4 million, 95% UI 1.0–1.8 million; DALY: 27.4 million, 95% UI 22.1–33.4 million) in 2019. The age-standardized death rate was decreased most by 64.9% (95% UI −67.1 to −62.8%) in the high SDI regions from 93.9 per 100,000 people (95% UI 70.9–119.7 per 100,000 people) in 1990 to 32.9 per 100,000 people (95% UI 24.0–43.5 per 100,000 people) in 2019. The age-standardized SEV and DALY rates were decreased significantly in the high SDI group (Supplementary Figures 3a–c). From 21 GBD geographic regions, a similar correlation between age-standardized death and DALY rates and SDI was observed from 1990 to 2019 (Supplementary Figures 4, 5). Of 204 countries and territories in 2019, similar trends were demonstrated to correlate age-standardized death and DALY rates between SDIs for both sexes (Supplementary Figures 6, 7).

**FIGURE 2:**
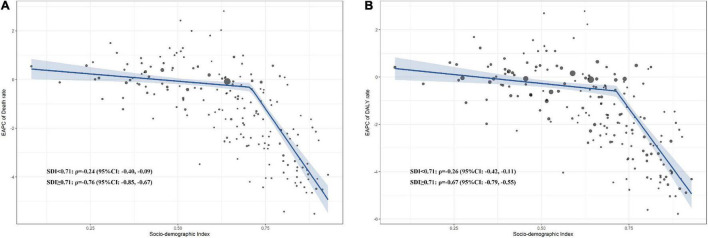
Correlation between EAPC of high LDL-C attributable age-standardized death and DALY rate in 2019. **(A)** Correlation between EAPC of high LDL-C attributable death and SDI; **(B)** Correlation between EAPC of high LDL-C attributable DALY and SDI. DALY, disability-adjusted life year; SDI, social-demographic index; EAPC, estimated annual percentage change; LDL-C, low-density lipoprotein-cholesterol.

The spline linear regression and Pearson correlation demonstrated that the age-standardized death EAPC was negatively correlated with SDI when SDI surpassed 0.71 (ρ − 0.76, 95% CI − 0.85 to − 0.67, *p* < 0.001; Figure 2A). Similarly, when SDI surpassed 0.71, the age-standardized DALY rates were negatively associated with SDI (ρ − 0.67, 95% CI − 0.79 to − 0.55, *p* < 0.001; Figure 2B). In the sensitivity analysis, the correlation trends were consistent in 1990, 2000, and 2010 for deaths and DALYs (Supplementary Figures 8–13).

## Discussion

High LDL-C remains a major challenge facing healthcare systems around the world. The global burden attributable to high LDL-C was increased by over 40% in the last 30 years, while the age-standardized burden was decreased. This inconsistent trend of absolute and age-standardized burden reflects the successful control of premature death and disability and the global aging process. From a global perspective, the general lipid-lowering interventions might have contributed to reducing the global burden (15). However, regions of different SDI quintiles show discrepancies in the high-LDL-C-related disease burden. Meanwhile, the proportion of age-standardized death and DALY rates of ischemic stroke and IHD attributable to high LDL-C was consistent for 30 years. From the above dimensions, this study helps to fill the gap in understanding about high LDL-C-related global burden and trends at the global, regional, and national levels by age, sex, and SDI.

We noticed that inflection points split the SDI levels into low to middle and middle to high regarding the association between SDI and high LDL-C-related age-standardized death and DALY rates. For high and high-middle SDI regions, the trends of age-standardized death and DALY rates have consistently decreased since 1994. Meanwhile, the age-standardized SEV rates of high and high-middle SDI regions showed significant decreasing trends since 1990. The middle, low-middle, and low SDI regions did not reach the peak of high LDL-C attributable disease burden. In comparison, the age-standardized SEV rates of those regions showed increasing trends since 1990. For high SDI regions, some countries/territories, such as Denmark, Israel, and the South Korea, saw the most significant decreases, over 70%, in age-standardized death and DALY rates. Those countries/territories shared similar experiences in robust primary care infrastructures, low medicine payment, high national medical insurance coverage, high-quality healthcare services, high-risk patient screening and education, and rich information infrastructure (16–19). For high-middle SDI regions, since the peak in 1994, the high LDL-C attributable disease burdens decreased gradually and stably. By analyzing the high LDL-C attributable disease burden trends, low SDI regions’ age-standardized deaths and DALYs have surpassed high SDI regions since 2003 and 1999, respectively. The trend shows that actions targeting lowering LDL-C are highly active. In addition, those regions demonstrated the most significant decrease in age-standardized DALY rates from 1990 to 2019, which might be driven by a considerable amount of age-standardized death rates, an indication of an aging population. According to the United Nations report, from 1990 to 2019, people aged over 65 years old were increased from 6 to 9% (20). These changes are consistent with the marked increase in statin use between the late 20th century and early 21st century (21–23). Further lowering LDL-C levels may require increased access to different lipid-lowering agents, such as ezetimibe and PCSK9 inhibitors (24–28).

For low to middle SDI regions, the LDL-C attributable disease burden still does not reach the peak point according to the trend. However, some countries/territories, including Uzbekistan, Philippines, Tajikistan, and Lesotho, deviated from the global trends and increased by over 50% in terms of age-standardized death and DALY rates. Though uncertainties remain in explaining increased deaths, a study in Uzbekistan suggests limited primary healthcare facilities, limited cardiovascular risk stratification among healthcare staff, and high medicine payment (29). Furthermore, a Philippines study suggests that limited data on national disease burden and a shortage of cost-effectiveness estimates for better prioritizing healthcare may also be playing a role (30). For low to middle SDI countries, health policies play a crucial role in prioritizing service coverage, educating healthcare staff about risk stratification and management, and implementing essential lipid-lowering therapy, healthy lifestyle modification, and healthcare monitoring systems (31–34). According to the WHO Global Atlas on Cardiovascular Disease Prevention and Control, trends toward urbanization, industrialization, and globalization have prolonged life expectancy in developing countries/territories. However, they have also exposed people to high LDL-C risk factors over time (35). With rises in SDI levels, low-income nations may see their major disease burden transition from maternal and child health to non-communicable chronic diseases. Soon, a high LDL-C disease burden could emerge gradually among low- to middle-income nations (36). There should be strategic timing of prevention and interventions to address LDL-C before the soaring disease burden for those regions. Disparities have existed in accessing primary care within different populations and countries, and the delay in lipid management could result in heavier complications and death. For low to middle SDI countries, stakeholders should be aware of the emerging high LDL-C-related disease burden. The policymakers could draw on the experience of high SDI regions and their practical actions targeting lowering high LDL-C. However, the local culture, ethnicity, and epidemiologic conditions determine the final policy for their people.

The present study summarizes and analyzes the global burden attributable to high LDL-C, filling the gap in understanding global burden, and trends at global, regional, and national levels by age, sex, and SDI. However, this study has some limitations. First, the GBD data used in this study represent many countries/territories and varying levels of quality. Therefore, some results may deviate from the actual situation. More regional epidemiological surveys could reduce this measurement error. In addition, the LDL-C attributable disease burden lacked data for many countries and territories. Therefore, the estimates could not represent all included countries and territories. Second, this ecological study could not be applied in specific situations and could only analyze general trends. Third, the linear regression for EAPC could not fit the countries’ development rates in curves. Last, SDI represents social development with mixed income, fertility, and education indexes. Therefore, it could not fully represent them with simplification. For chronic non-communicable diseases, education and income play crucial roles.

This study shows that high LDL-C remains a major challenge for healthcare systems around the world. The absolute number of high LDL-C attributable deaths and DALYs showed increasing trends from 1990 to 2019. However, age-standardized SEV, death, and DALY rates showed a decreasing trend due to the global aging process. Successful healthcare policies and drugs targeting high LDL-C can reduce the burden of high LDL-C, including ischemic stroke and IHD. The high LDL-C attributable disease burden is negatively associated with the SDI levels in moderate to high SDI regions. Recently, new challenges have increased. For higher SDI regions, new and different drugs could further reduce the LDL-C level. For lower SDI regions, attention should be drawn to initiating policies and increasing access to drugs and other interventions to reduce LDL-C levels within the population.

## Data Availability Statement

Publicly available datasets were analyzed in this study. This data can be found here: https://ghdx.healthdata.org.

## Author Contributions

HD, QS, and SL designed the study. HD and QS collected and analyzed the data, prepared the results, and wrote the manuscript. PS, X-FP, XY, LC, YH, GZ, and BS contributed to the interpretation of the results and revised the manuscript. All authors contributed to the article and approved the submitted version.

## Conflict of Interest

The authors declare that the research was conducted in the absence of any commercial or financial relationships that could be construed as a potential conflict of interest.

## Publisher’s Note

All claims expressed in this article are solely those of the authors and do not necessarily represent those of their affiliated organizations, or those of the publisher, the editors and the reviewers. Any product that may be evaluated in this article, or claim that may be made by its manufacturer, is not guaranteed or endorsed by the publisher.

## Footnotes

1
http://ghdx.healthdata.org/gbd-results-tool

## References

[1] United Nations. *17 Goals to Transform Our World.* New York, NY: United Nations (2021).

[2] Reddy KS. Global burden of disease study 2015 provides GPS for global health 2030. *Lancet.* (2016) 388:1448–9. 10.1016/S0140-6736(16)31743-3 27733278

[3] Virani SS Alonso A Benjamin EJ Bittencourt MS Callaway CW Carson AP Heart disease and stroke statistics-2020 update: a report from the American heart association. *Circulation.* (2020) 141:e139–596. 10.1161/CIR.0000000000000757 31992061

[4] Nelson RH. Hyperlipidemia as a risk factor for cardiovascular disease. *Prim Care.* (2013) 40:195–211. 23402469 10.1016/j.pop.2012.11.003 PMC3572442

[5] Zeng L Ye Z Li Y Zhou Y Shi Q Hu T Low-density lipoprotein cholesterol and novel lipid parameters in predicting clinical outcomes in Chinese statin-naïve patients after coronary stent implantation. *Front Cardiovasc Med.* (2021) 8:638663. 10.3389/fcvm.2021.638663 33796571 PMC8007761

[6] Wenger N Boden W Carabello B Carney R Cerqueira M Criqul M. *Cardiovascular Disability: Updating the Social Security Listings.* Washington, DC: National Academy of Sciences (2010).

[7] Prospective Studies Collaboration. Blood cholesterol and vascular mortality by age, sex, and blood pressure: a meta-analysis of individual data from 61 prospective studies with 55 000 vascular deaths. *Lancet.* (2007) 370:1829–39. 10.1016/S0140-6736(07)61778-4 18061058

[8] Feigin VL Nichols E Alam T Bannick MS Beghi E Blake N Global, regional, and national burden of neurological disorders, 1990–2016: a systematic analysis for the global burden of disease study 2016. *Lancet Neurol.* (2019) 18:459–80. 10.1016/S1474-4422(18)30499-X 30879893 PMC6459001

[9] Butowski P Winder A. Usual care dietary practice, achievement and implications for medication in the management of hypercholesterolaemia. Data from the UK lipid clinics programme. *Eur Heart J.* (1998) 19:1328–33. 10.1053/euhj.1998.1044 9792257

[10] Schedlbauer A Schroeder K Peters TJ Fahey T. Interventions to improve adherence to lipid lowering medication. *Cochrane Database Syst Rev.* (2004) 12:CD004371. 10.1002/14651858.CD004371.pub2 15495105 PMC4163627

[11] Global Burden of Disease Collaborative Network. *Global Burden of Disease Study 2019 (GBD 2019) Results.* Seattle, WA: Global Burden of Disease Collaborative Network (2020).

[12] Collaborators Group. Global burden of 87 risk factors in 204 countries and territories, 1990–2019: a systematic analysis for the global burden of disease study 2019. *Lancet.* (2020) 396:1223–49. 33069327 10.1016/S0140-6736(20)30752-2 PMC7566194

[13] Stevens GA Alkema L Black RE Boerma JT Collins GS Ezzati M Guidelines for accurate and transparent health estimates reporting: the GATHER statement. *PLoS Med.* (2016) 13:e1002056. 10.1016/S0140-6736(16)30388-9 PMC4924581 27351744

[14] Murray CJ Aravkin AY Zheng P Abbafati C Abbas KM Abbasi-Kangevari M Global burden of 87 risk factors in 204 countries and territories, 1990–2019: a systematic analysis for the global burden of disease study 2019. *Lancet.* (2020) 396:1223–49. 10.1016/S0140-6736(20)30752-2 33069327 PMC7566194

[15] Pencina MJ Pencina KM Lloyd-Jones D Catapano AL Thanassoulis G Sniderman AD. The expected 30-year benefits of early versus delayed primary prevention of cardiovascular disease by lipid lowering. *Circulation.* (2020) 142:827–37. 10.1161/CIRCULATIONAHA.120.045851 32700572

[16] Olejaz M Juul Nielsen A Rudkjobing A Okkels Birk H Krasnik A Hernandez-Quevedo C. Denmark health system review. *Health Syst Transit.* (2012) 14:i–xxii. 22575801

[17] Forde I Nader C Socha-Dietrich K Oderkirk J Colombo F. *Primary Care Review of Denmark.* Paris: Organisation for Economic Co-operation Development (2016). p. 6–16.

[18] Clarfield AM Manor O Nun GB Shvarts S Azzam ZS Afek A Health and health care in Israel: an introduction. *Lancet.* (2017) 389:2503–13. 10.1016/S0140-6736(17)30636-0 28495109

[19] OECD. *OECD Health Care Quality Review: Korea.* Paris: Organization for Economic Cooperation and Development Publishing (2012).

[20] World Health Organization. *Ageing.* Geneva: World Health Organization (2020).

[21] Riahi S Fonager K Toft E Hvilsted-Rasmussen L Bendsen J Paaske Johnsen S Use of lipid-lowering drugs during 1991–98 in Northern Jutland, Denmark. *Br J Clin Pharmacol.* (2001) 52:307–11. 10.1046/j.0306-5251.2001.01439.x 11560563 PMC2014543

[22] Walley T Folino-Gallo P Stephens P Van Ganse E. Trends in prescribing and utilization of statins and other lipid lowering drugs across Europe 1997-2003. *Br J Clin Pharmacol.* (2005) 60:543–51. 10.1111/j.1365-2125.2005.02478.x 16236045 PMC1884951

[23] Gu Q Kit BK. Prescription cholesterol-lowering medication use in adults aged 40 and over: United States, 2003–2012. *NCHS Data Brief.* (2014) 177:1–8. 25536410

[24] Du H Li X Su N Li L Hao X Gao H Proprotein convertase subtilisin/kexin 9 inhibitors in reducing cardiovascular outcomes: a systematic review and meta-analysis. *Heart.* (2019) 105:1149–59. 10.1136/heartjnl-2019-314763 30842207

[25] Hao Q Aertgeerts B Guyatt G Bekkering GE Vandvik PO Khan SU PCSK9 inhibitors and ezetimibe for the reduction of cardiovascular events: a clinical practice guideline with risk-stratified recommendations. *BMJ.* (2022) 377:e069066. 10.1136/bmj-2021-069066 35508320

[26] Khan SU Yedlapati SH Lone AN Hao Q Guyatt G Delvaux N PCSK9 inhibitors and ezetimibe with or without statin therapy for cardiovascular risk reduction: a systematic review and network meta-analysis. *BMJ.* (2022) 377:e069116. 10.1136/bmj-2021-069116 35508321

[27] Wang Y Zhan S Du H Li J Khan SU Aertgeerts B Safety of ezetimibe in lipid-lowering treatment: systematic review and meta-analysis of randomised controlled trials and cohort studies. *BMJ Med.* (2022) 1:e000134. 10.1136/bmjmed-2022-000134 PMC10012858 36936552

[28] Li J Du H Wang Y Aertgeerts B Guyatt G Hao Q Safety of proprotein convertase subtilisin/kexin 9 inhibitors: a systematic review and meta-analysis. *Heart.* (2022). 10.1136/heartjnl-2021-320556 35508401

[29] Farrington J Kontsevaya A Small R Ermakova Y Kulikov A Gamgabeli L. *Prevention and Control of Noncommunicable Diseases in Uzbekistan.* Geneva: World Health Organization (2018).

[30] Wong JQ Uy J Haw NJL Valdes JX Bayani DBS Bautista CAP Priority setting for health service coverage decisions supported by public spending: experience from the Philippines. *Health Syst Reform.* (2018) 4:19–29.

[31] Azizi F Ghanbarian A Momenan AA Hadaegh F Mirmiran P Hedayati M Prevention of non-communicable disease in a population in nutrition transition: Tehran lipid and glucose study phase II. *Trials.* (2009) 10:5. 10.1186/1745-6215-10-5 19166627 PMC2656492

[32] Sdringola S Nakagawa K Nakagawa Y Yusuf SW Boccalandro F Mullani N Combined intense lifestyle and pharmacologic lipid treatment further reduce coronary events and myocardial perfusion abnormalities compared with usual-care cholesterol-lowering drugs in coronary artery disease. *J Am Coll Cardiol.* (2003) 41:263–72. 10.1016/s0735-1097(02)02693-1 12535820

[33] The Lancet. The Astana declaration: the future of primary health care? *Lancet.* (2018) 392:1369. 10.1016/S0140-6736(18)32478-4 30343840

[34] World Health Organization. *Hearts: Technical Package for Cardiovascular Disease Management in Primary Health Care.* Geneva: World Health Organization (2020).

[35] Mendis S Puska P Norrving B. *Global Atlas on Cardiovascular Disease Prevention and Control.* Geneva: World Health Organization (2011).

[36] Schröders J Wall S Hakimi M Dewi FST Weinehall L Nichter M How is Indonesia coping with its epidemic of chronic noncommunicable diseases? A systematic review with meta-analysis. *PLoS One.* (2017) 12:e0179186. 10.1371/journal.pone.0179186 28632767 PMC5478110

