# Supplementary material for: Global Burden Attributable to High Low-Density Lipoprotein-Cholesterol From 1990 to 2019
Source: Front Cardiovasc Med. 2022 Jun 9;9:903126. doi: 10.3389/fcvm.2022.903126 (PMC9218272; doi:10.3389/fcvm.2022.903126)
Supplement: Supplementary file 2 [file Data_Sheet_2.docx]

**Supplementary Figure 1 Ranks of age standardized rate of DALY and death attributable to high LDL-C of globe and 5 SDI levels from 1990 to 2019.**


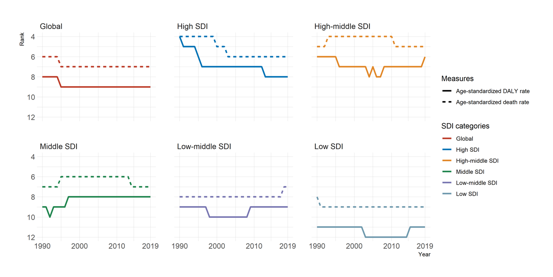


Abbreviations: DALY: disability adjusted life year; SDI: social-demographic index.

**Supplementary Figure 2 Age specific number and rate of high LDL-C attributable DALY and death in males and females of globe and 5 SDI levels.**

1. Age specific number and rate of high LDL-C attributable DALY in males and females of globe


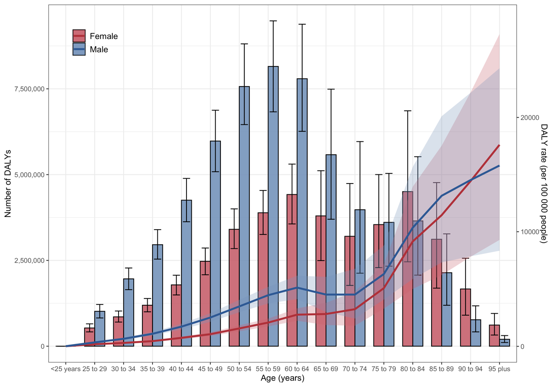


1. Age specific number and rate of high LDL-C attributable DALY in males and females of low-SDI regions;


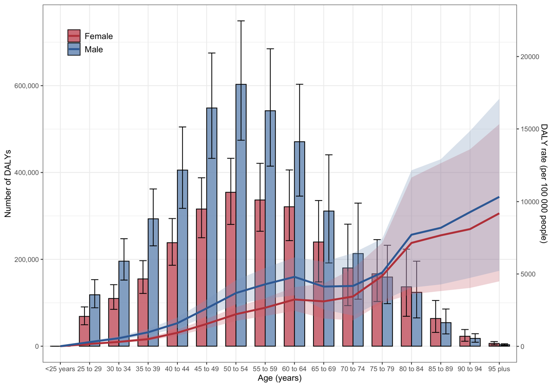


1. Age specific number and rate of high LDL-C attributable DALY in males and females of low-middle-SDI regions;


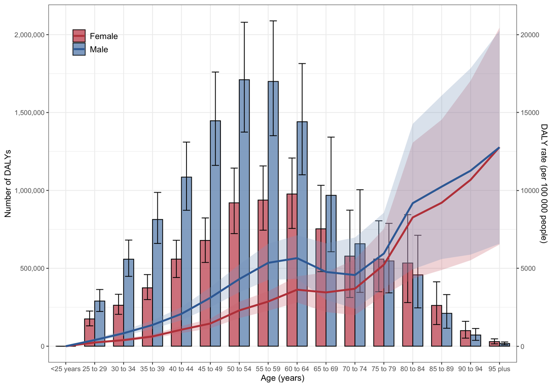


1. Age specific number and rate of high LDL-C attributable DALY in males and females of middle-SDI regions;


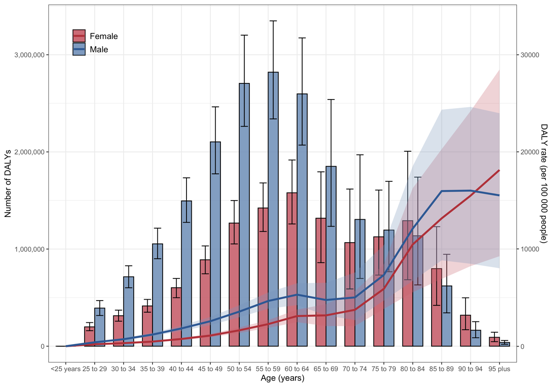


1. Age specific number and rate of high LDL-C attributable DALY in males and females of high-middle-SDI regions;


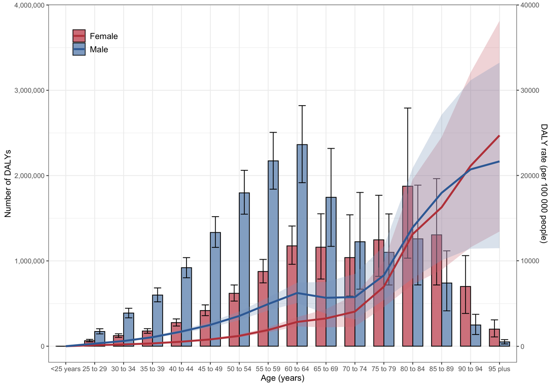


(f)Age specific number and rate of high LDL-C attributable DALY in males and females of high-SDI regions;


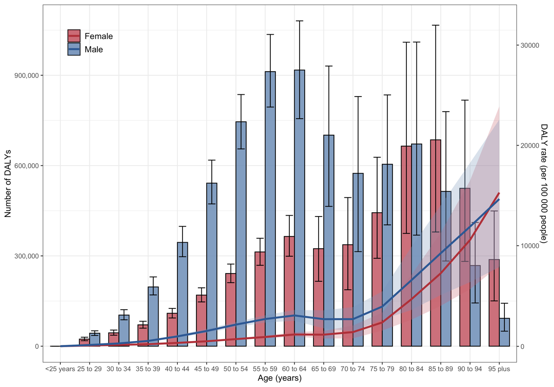


1. Age specific number and rate of high LDL-C attributable death in males and females of globe;


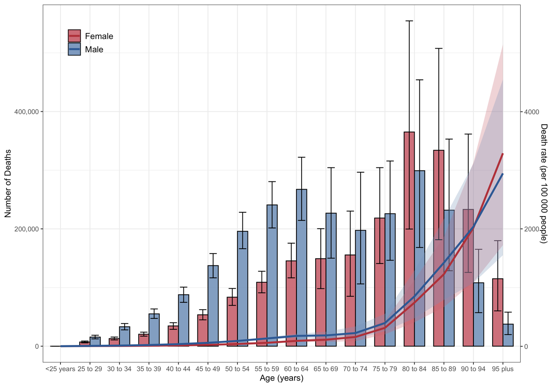


1. Age specific number and rate of high LDL-C attributable death in males and females of low-SDI regions;


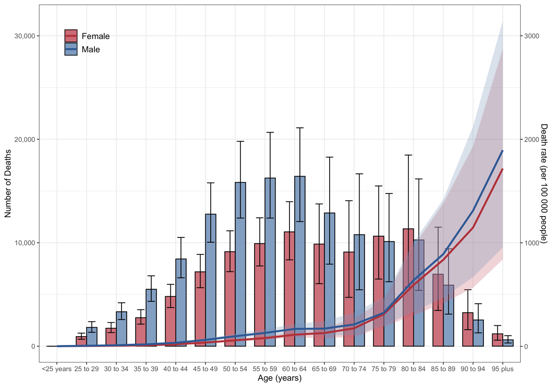


1. Age specific number and rate of high LDL-C attributable death in males and females of low-middle-SDI regions;


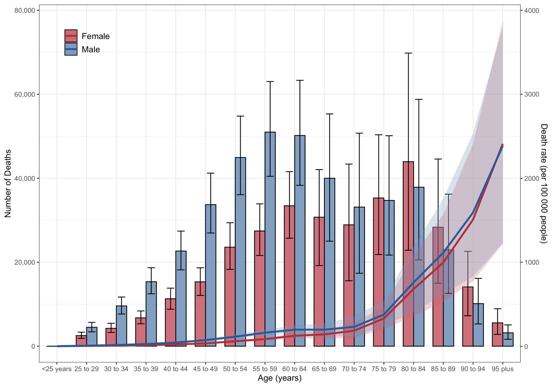


1. Age specific number and rate of high LDL-C attributable death in males and females of middle-SDI regions;


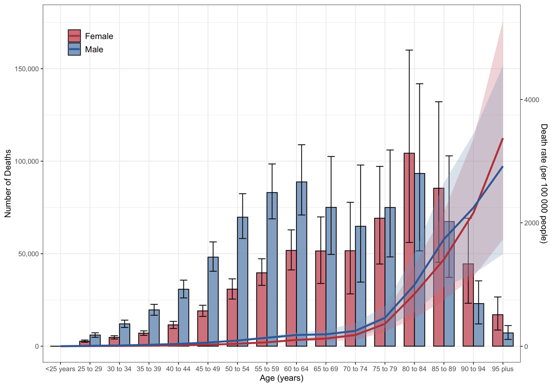


1. Age specific number and rate of high LDL-C attributable death in males and females of high-middle-SDI regions;


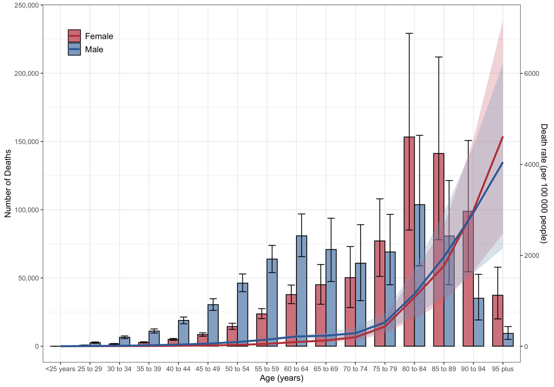


1. Age specific number and rate of high LDL-C attributable death in males and females of high-SDI regions.


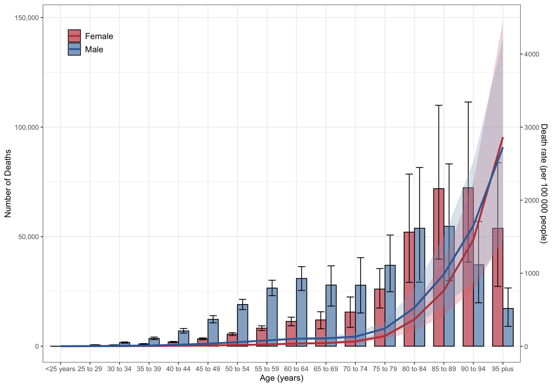


Abbreviations: DALY: disability adjusted life year; SDI: social-demographic index.

**Supplementary Figure 3 Changes in high LDL-C attributable age-standardized DALY and death rates trends of 5 SDI levels over 30 years.**

1. Changes in age-standardized SEV rates trends of 5 SDI levels over 30 years.


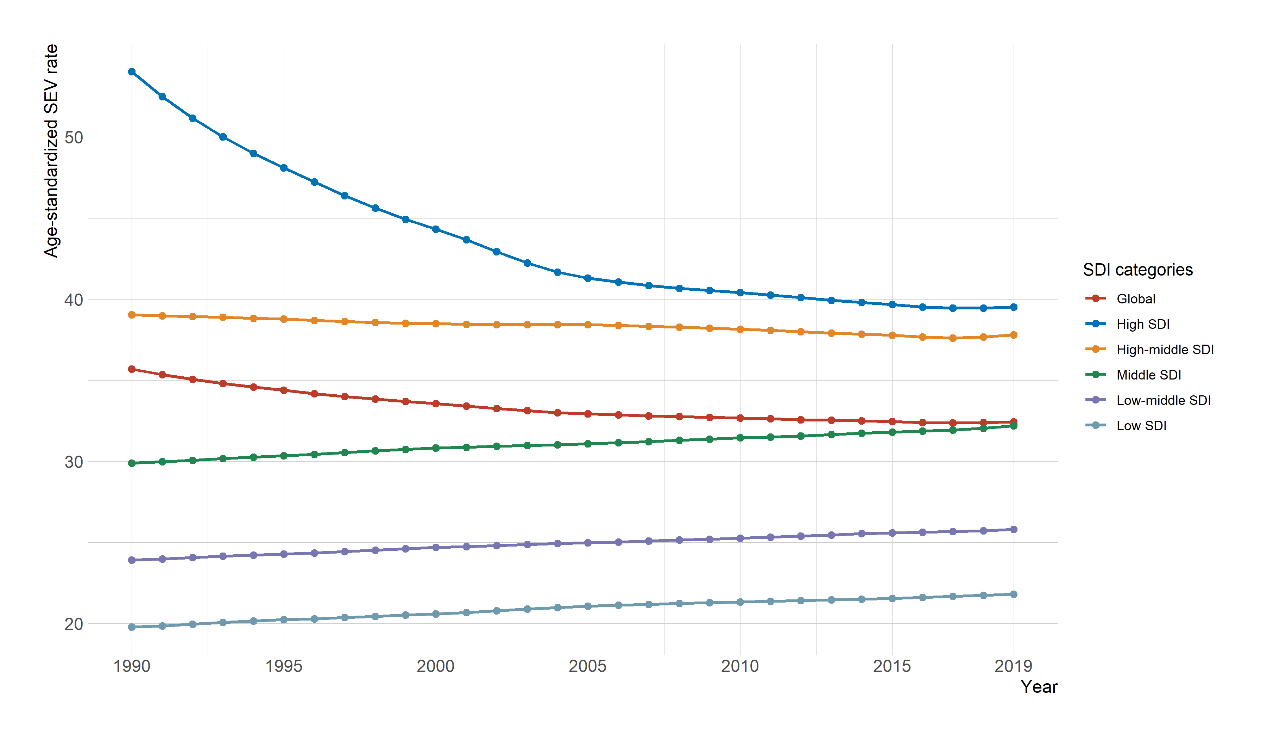


1. Changes in age-standardized DALY rates trends of 5 SDI levels over 30 years;


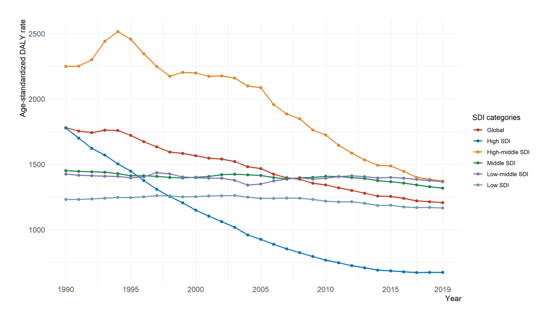


1. Changes in age-standardized death rates trends of 5 SDI levels over 30 years.


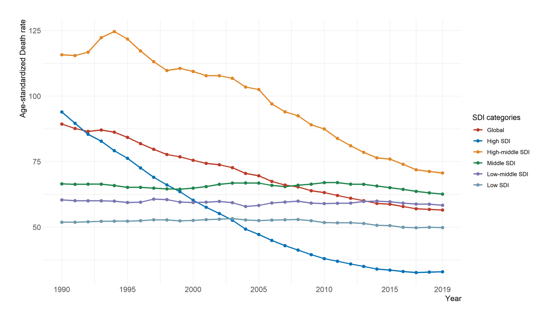


Abbreviations: DALY: disability adjusted life year; SDI: social-demographic index; SEV, summary exposure value.

**Supplementary Figure 4 Age-standardized DALY rates attributable to high LDL-C across 21 GBD regions for both sexes 1990-2019**


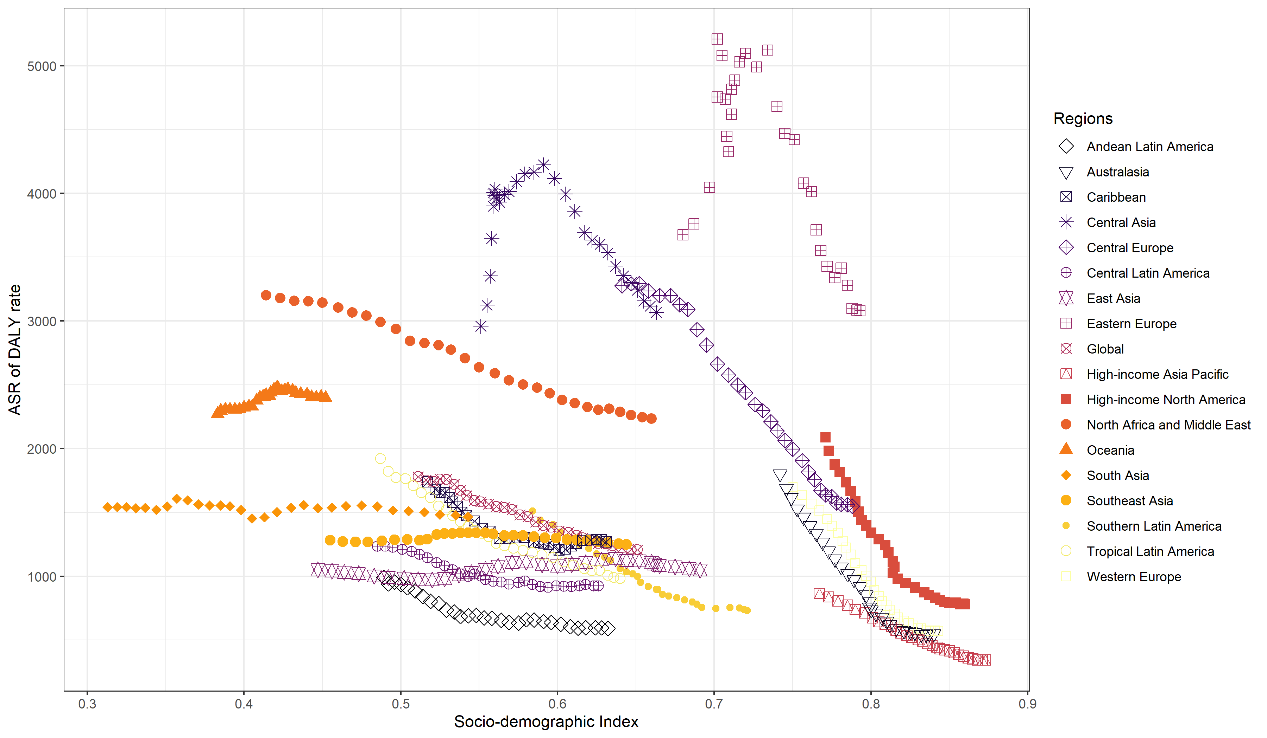


Figure legends: DALY: disability adjusted life year; ASR: age-standardized rate.

**Supplementary Figure 5 Age-standardized Death rates attributable to high LDL-C across 21 GBD regions for both sexes 1990-2019**


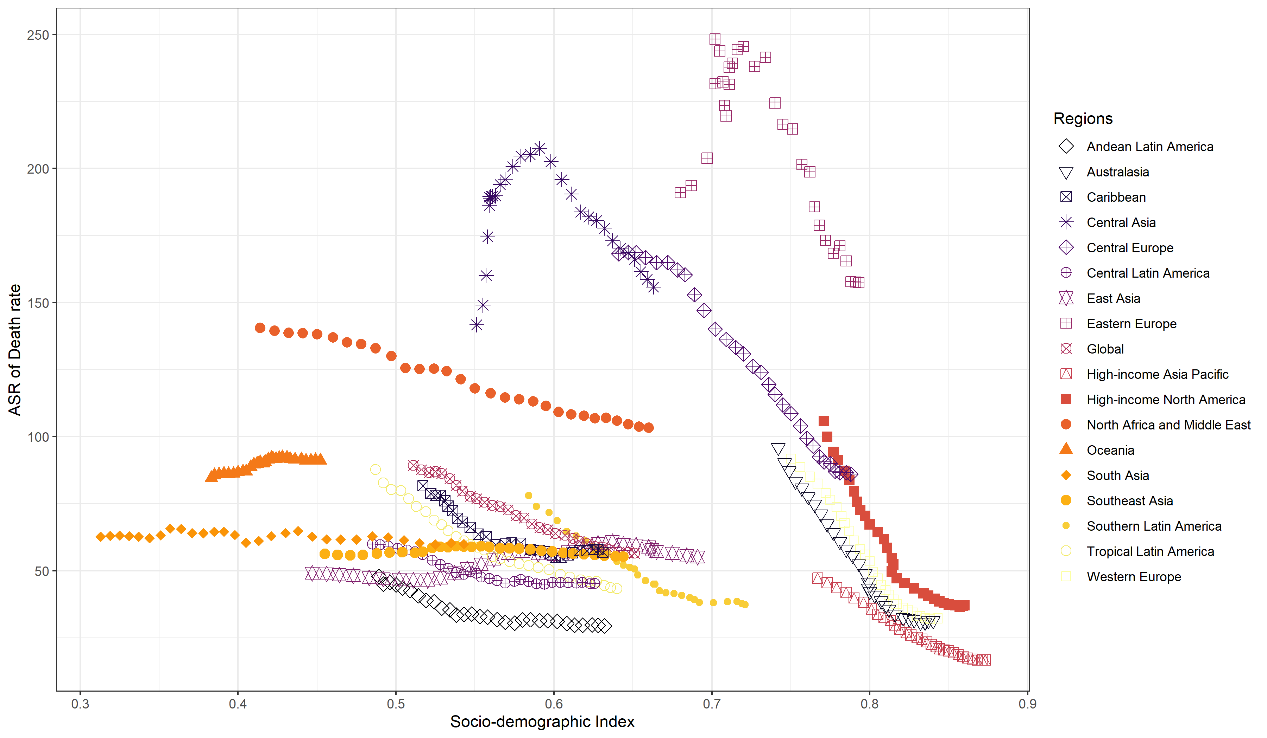


Figure legends: ASR: age-standardized rate.

**Supplementary Figure 6 Age-standardized DALY rates attributable to high LDL-C across 204 countries and territories for both sexes 1990-2019**


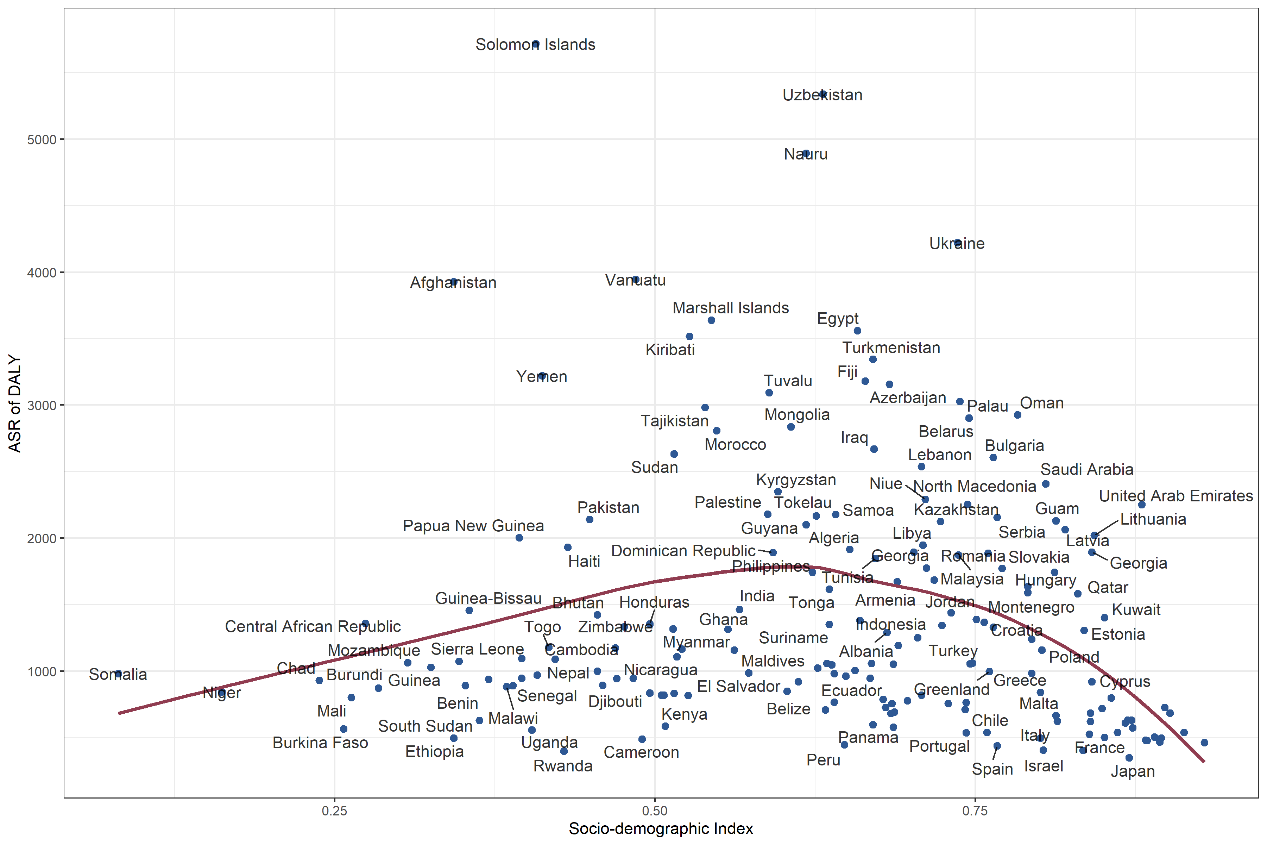


Figure legends: DALY: disability adjusted life year; ASR: age-standardized rate.

**Supplementary Figure 7 Age-standardized Death rates attributable to high LDL-C across 204 countries and territories for both sexes 1990-2019**


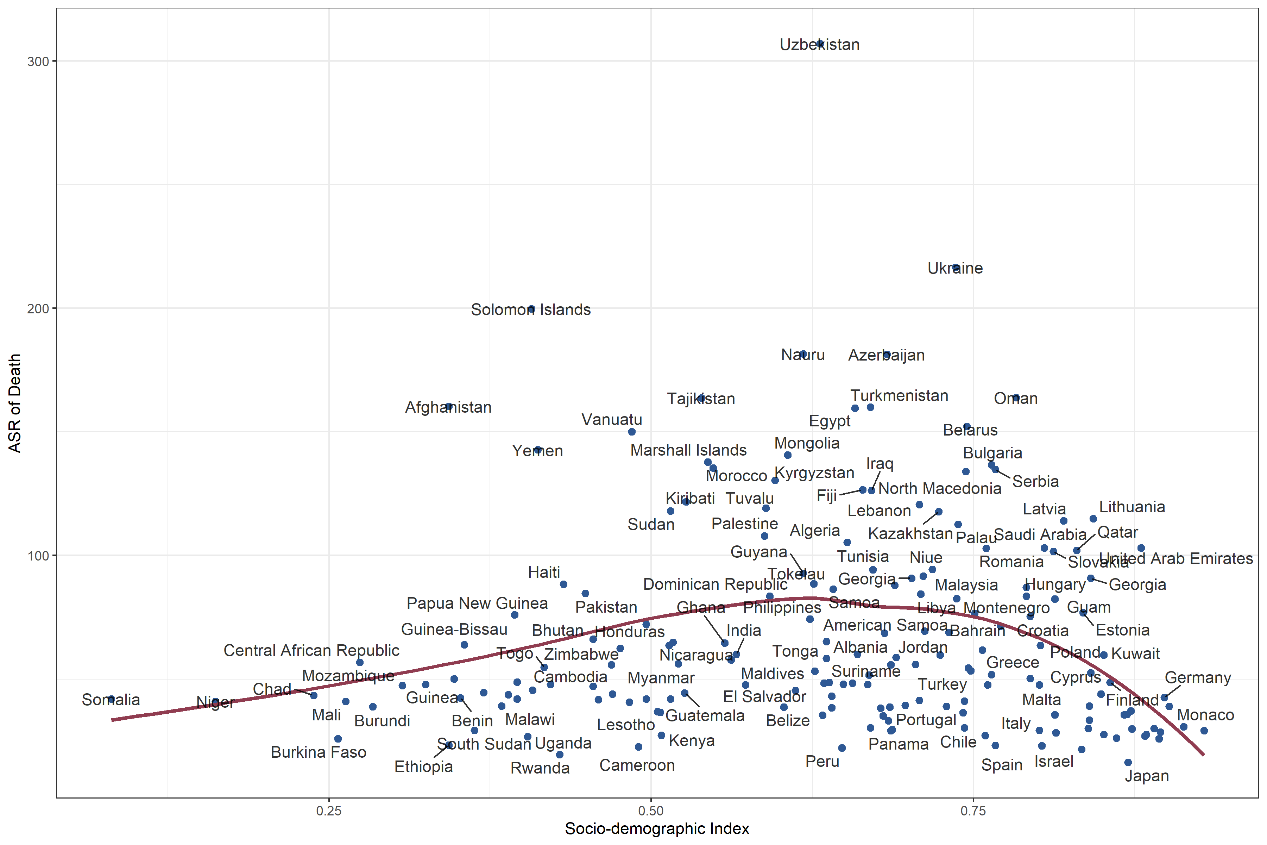


Figure legends: ASR: age-standardized rate.

**Supplementary Figure 8 Correlation between EAPC of high LDL-C attributable age-standardized death rate in 1990.**


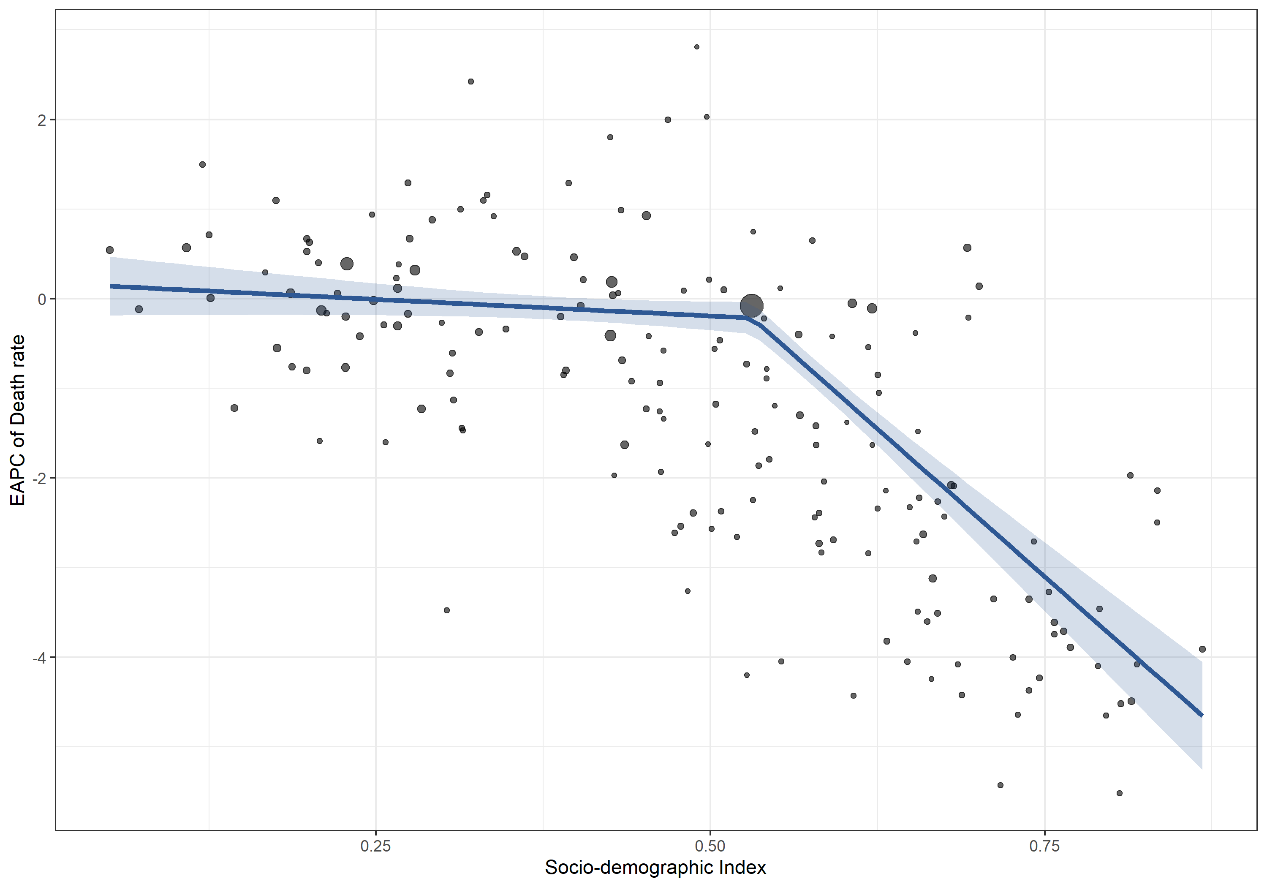


Figure legends: SDI: social-demographic index; EAPC: estimated annual percentage change.

**Supplementary Figure 9 Correlation between EAPC of high LDL-C attributable age-standardized DALY rate in 1990.**


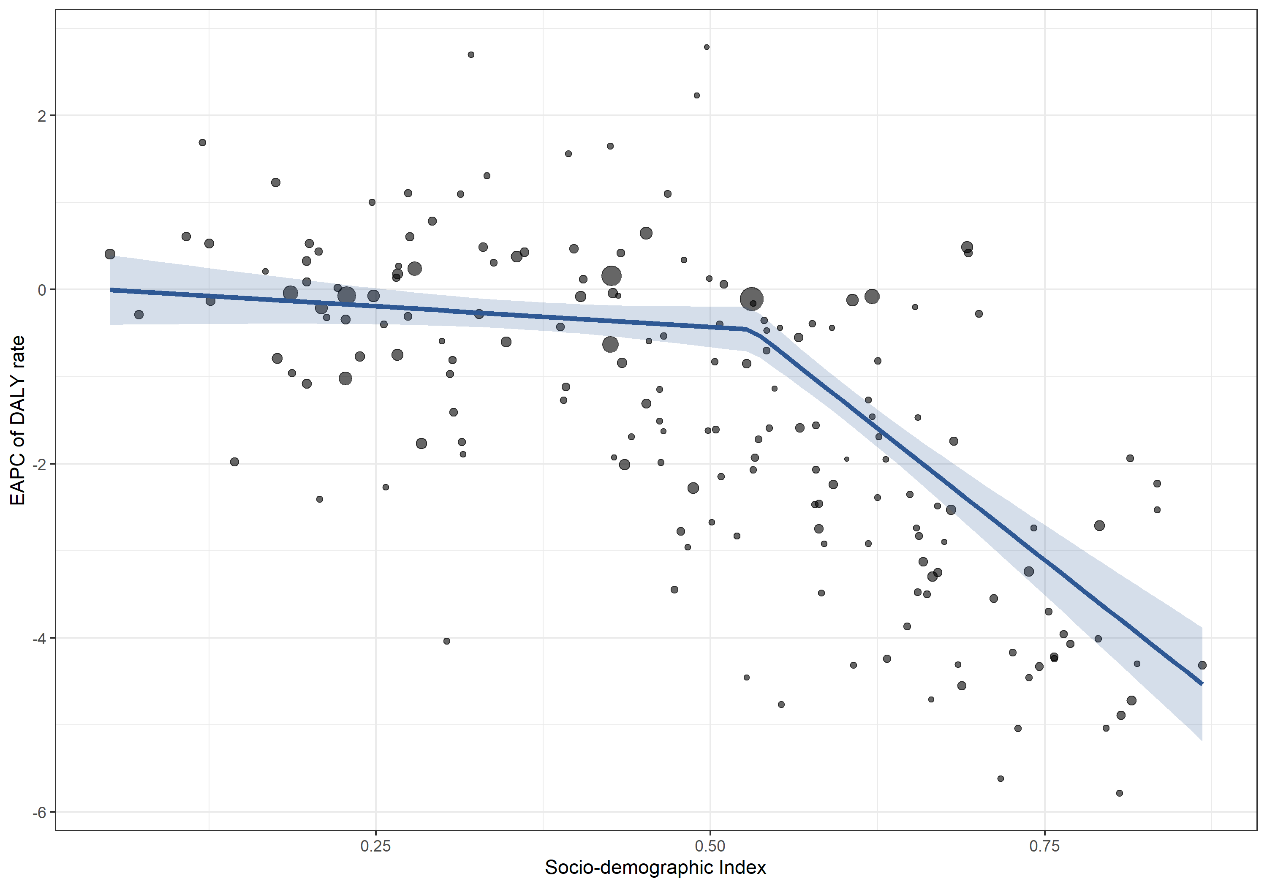


Figure legends: DALY: disability adjusted life year; SDI: social-demographic index; EAPC: estimated annual percentage change

**Supplementary Figure 10 Correlation between EAPC of high LDL-C attributable age-standardized death rate in 2000.**


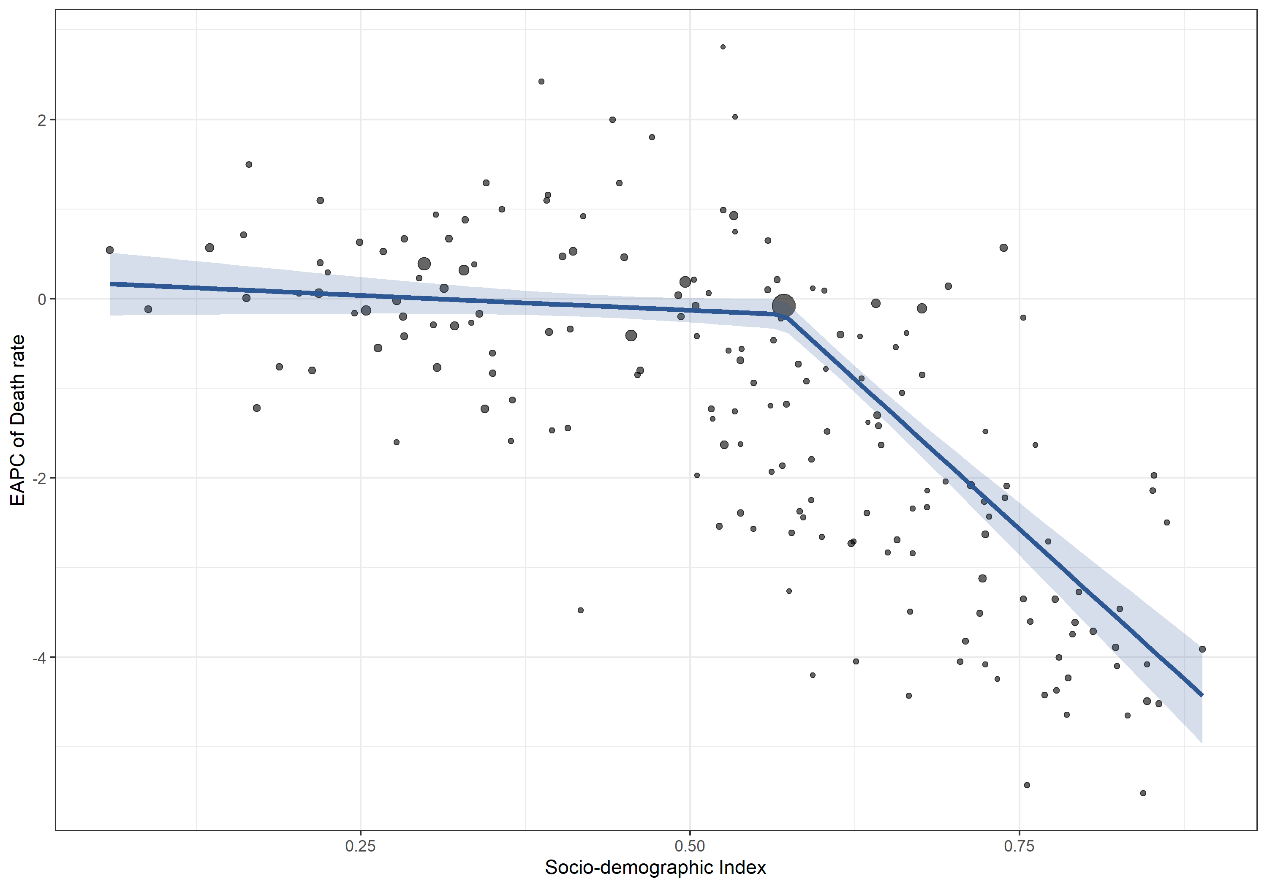


Figure legends: SDI: social-demographic index; EAPC: estimated annual percentage change

**Supplementary Figure 11 Correlation between EAPC of high LDL-C attributable age-standardized DALY rate in 2000.**


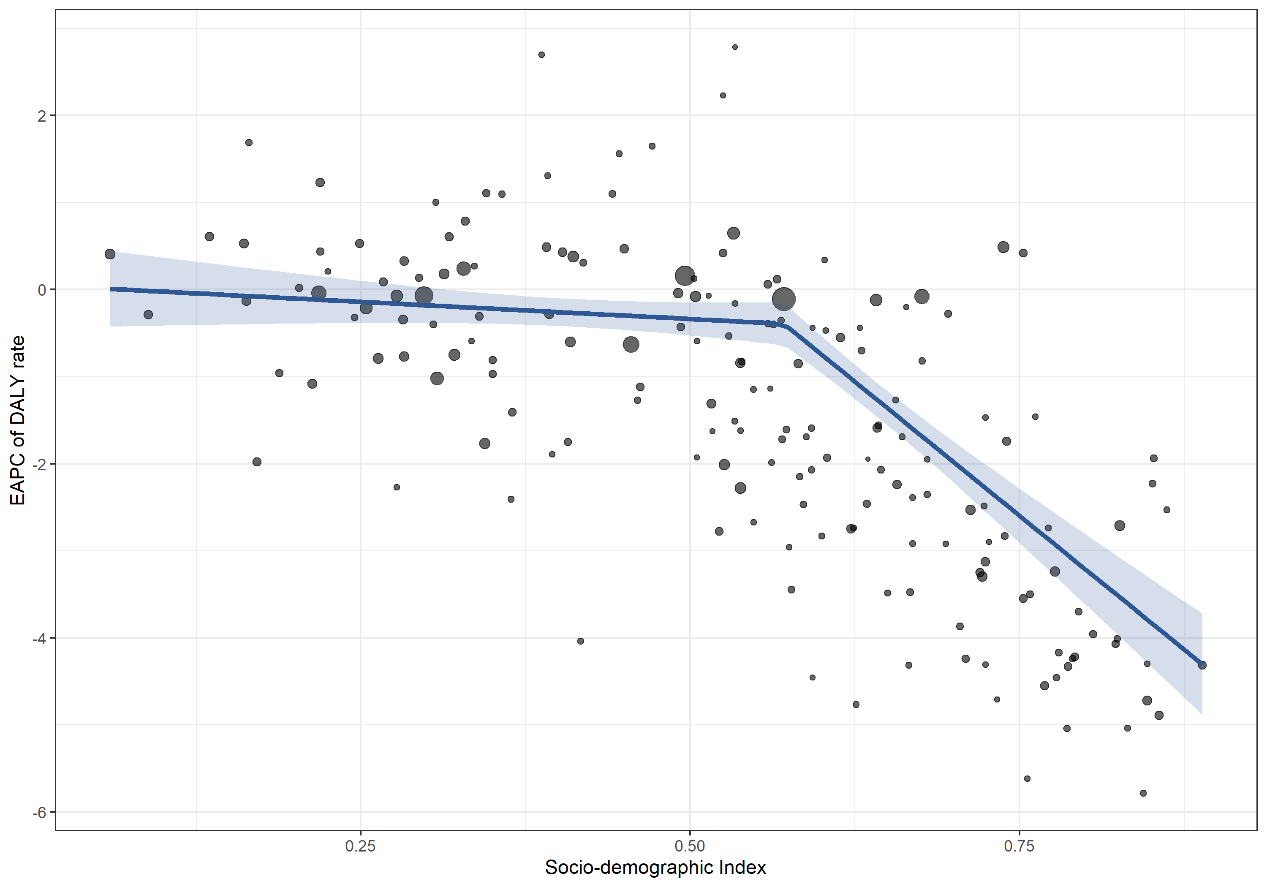


Figure legends: DALY: disability adjusted life year; SDI: social-demographic index; EAPC: estimated annual percentage change

**Supplementary Figure 12 Correlation between EAPC of high LDL-C attributable age-standardized death rate in 2010.**


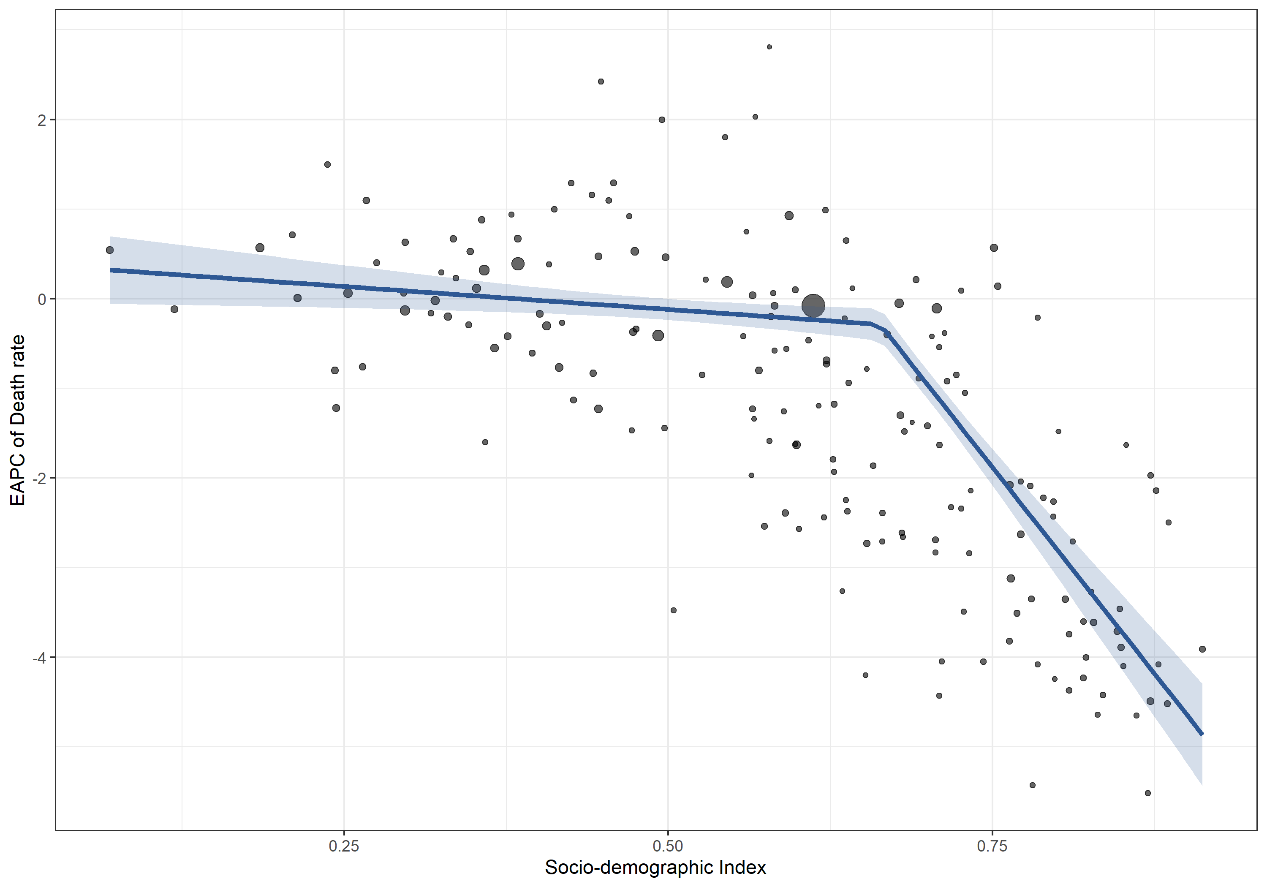


Figure legends: SDI: social-demographic index; EAPC: estimated annual percentage change

**Supplementary Figure 13 Correlation between EAPC of high LDL-C attributable age-standardized DALY rate in 2010.**


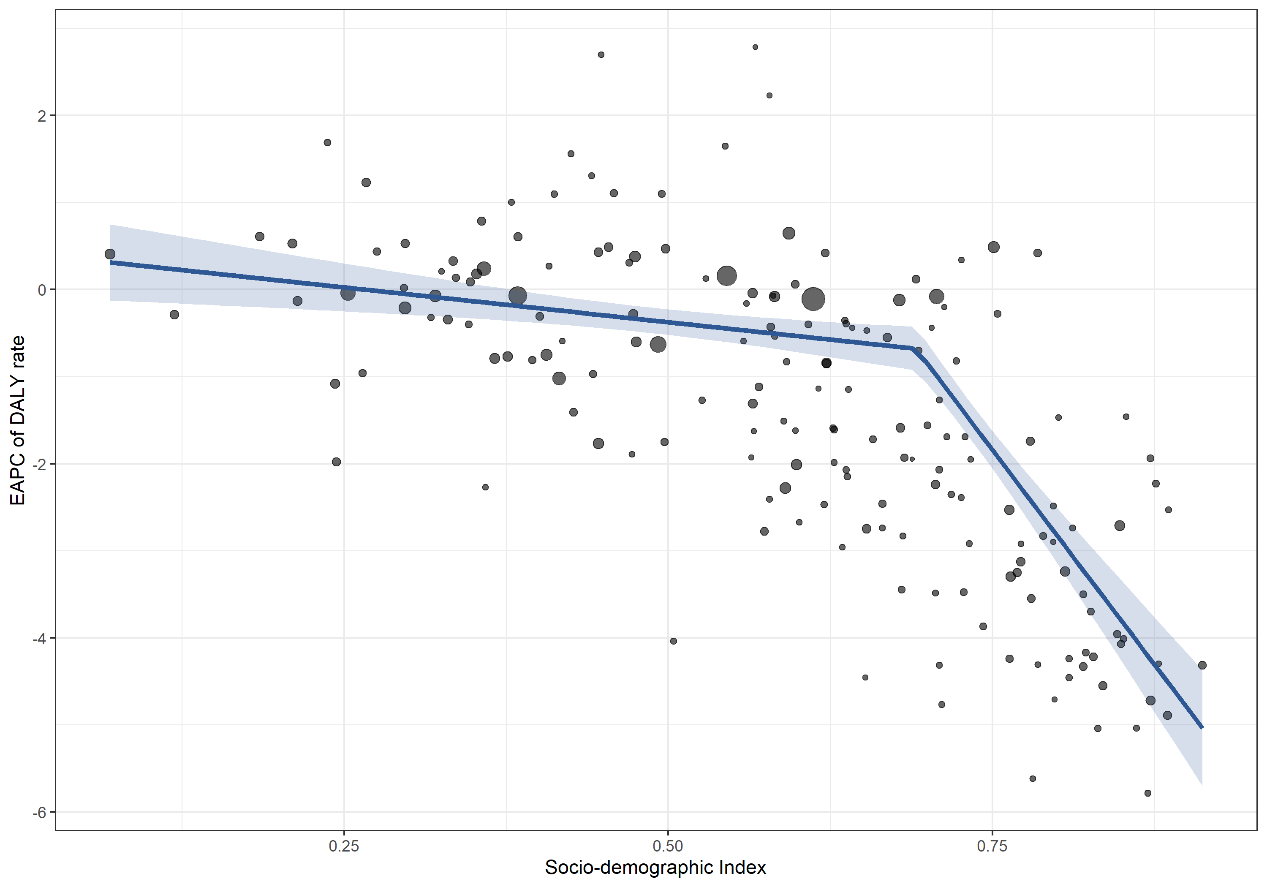


Figure legends: DALY: disability adjusted life year; SDI: social-demographic index; EAPC: estimated annual percentage change
