# Supplementary material for: Global Burden Attributable to High Low-Density Lipoprotein-Cholesterol From 1990 to 2019
Source: Front Cardiovasc Med. 2022 Jun 9;9:903126. doi: 10.3389/fcvm.2022.903126 (PMC9218272; doi:10.3389/fcvm.2022.903126)
Supplement: Supplementary file 3 [file Data_Sheet_3.docx]

# Supplementary table document

# Supplementary Table 1 Definitions and approaches to reach the major metrics of disease burden

| **Definitions and approaches** | |
| --- | --- |
| Disability-adjusted life of years (DALY) | DALY means the total number of years lost due to premature death and the number of years of disabled life. One DALY equals full health one year lost. [1] |
| Social-demographic index (SDI) | SDI is a new composite indicator for rating the development spectrum from 0 to 1, calculated through income per capita, fertility rates, and average educational attainment. [2] The SDI is applied in categorized countries and territories into five levels (high, high-middle, middle, low-middle, and low SDI). |
| Age stratification | We stratified age into those categories including age under 24, 25-29, 30-34, 35-39, 40-44, 45-49, 50-54, 55-59, 60-64, 65-69, 70-74, 75-79, 80- 84, 85- 89, 90-94, 95 plus years |
| Estimated annual percentage changes (EAPC) | The EAPC quantify the trend of age-standardized death rate (ASDR), and age-standardized DALY rate. The mathematical formulae of EAPC have applied the regression model in fitting age-standardized data from 1990 to 2019 into y = α + βx + ϵ, where y = ln (rate) and x = calendar year. Moreover, the EAPC was calculated from the 100×(exp(β) – 1), with its 95% confidence interval. [3] |
| Age-standardized data | The age-standardized data could exclude the effect of ages. |
| Summary exposure values (SEVs) | The SEV summarizes populational exposure distribution to a risk factor, which includes the extent and severity of risk exposures. The SEV is a risk-weighted prevalence of an exposure. The SEV compares the distribution of excess risk times exposure level to a population where everyone is at maximum risk. For a given risk factor r, the SEV was calculated for multiple related outcomes c and then was averaged across the outcomes. [4]  ${SEV}_{rc}=\frac{\int_{x=l}^{u} P\left( x \right)RR\left( x \right)dx-1}{{RR}_{max}-1}$  ${SEV}_{r}=\frac{1}{N(c)}\sum_{c} {SEV}_{rc}$ |

**Reference**:

1. Sassi FJHp, planning. Calculating QALYs, comparing QALY and DALY calculations. 2006;21(5):402-8.

2. IHME. Socio-demographic Index (SDI) 2021 [cited 2021 February 16]. Available from: <http://www.healthdata.org/taxonomy/glossary/socio-demographic-index-sdi>.

3. Liu Q, He H, Yang J, Feng X, Zhao F, Lyu JJJopr. Changes in the global burden of depression from 1990 to 2017: Findings from the Global Burden of Disease study. 2020;126:134-40.

4. IHME. Terms Defined Summary exposure value (SEV) 2021 [cited 2021 October 7]. Available from: <http://www.healthdata.org/terms-defined/s>.

# Supplementary Table 2 Age-standardized DALY attributable top 10 grade 2 level risk factors in different SDI regions of 1990 and 2019

| **Ranks** | **1990 global** | **1990 high SDI** | **1990 high-middle SDI** | **1990 middle SDI** | **1990 low-middle SDI** | **1990 low SDI** | **2019 global** | **2019 high SDI** | **2019 high-middle SDI** | **2019 middle SDI** | **2019 low-middle SDI** | **2019 low SDI** |
| --- | --- | --- | --- | --- | --- | --- | --- | --- | --- | --- | --- | --- |
| **1** | Child and maternal malnutrition | Tobacco | Tobacco | Child and maternal malnutrition | Child and maternal malnutrition | Child and maternal malnutrition | Child and maternal malnutrition | Tobacco | Tobacco | High systolic blood pressure | Child and maternal malnutrition | Child and maternal malnutrition |
| **2** | Air pollution | High systolic blood pressure | High systolic blood pressure | Air pollution | Air pollution | Unsafe water, sanitation, and handwashing | High systolic blood pressure | High body-mass index | High systolic blood pressure | Tobacco | Air pollution | Air pollution |
| **3** | Tobacco | Dietary risks | Dietary risks | Tobacco | Unsafe water, sanitation, and handwashing | Air pollution | Air pollution | High fasting plasma glucose | Dietary risks | Dietary risks | High systolic blood pressure | Unsafe water, sanitation, and handwashing |
| **4** | High systolic blood pressure | High LDL cholesterol | Child and maternal malnutrition | High systolic blood pressure | Tobacco | High systolic blood pressure | Tobacco | High systolic blood pressure | High body-mass index | Air pollution | Tobacco | High systolic blood pressure |
| **5** | Unsafe water, sanitation, and handwashing | High body-mass index | Air pollution | Dietary risks | High systolic blood pressure | Tobacco | Dietary risks | Dietary risks | High fasting plasma glucose | High fasting plasma glucose | High fasting plasma glucose | High fasting plasma glucose |
| **6** | Dietary risks | High fasting plasma glucose | High LDL cholesterol | High fasting plasma glucose | Dietary risks | Dietary risks | High fasting plasma glucose | Alcohol use | High LDL cholesterol | Child and maternal malnutrition | Dietary risks | Dietary risks |
| **7** | High fasting plasma glucose | Alcohol use | High body-mass index | Unsafe water, sanitation, and handwashing | High fasting plasma glucose | Unsafe sex | High body-mass index | Drug use | Air pollution | High body-mass index | High body-mass index | Tobacco |
| **8** | High LDL cholesterol | Child and maternal malnutrition | High fasting plasma glucose | Occupational risks | Occupational risks | High fasting plasma glucose | Unsafe water, sanitation, and handwashing | High LDL cholesterol | Alcohol use | High LDL cholesterol | Unsafe water, sanitation, and handwashing | High body-mass index |
| **9** | High body-mass index | Air pollution | Alcohol use | High LDL cholesterol | High LDL cholesterol | Alcohol use | High LDL cholesterol | Occupational risks | Child and maternal malnutrition | Kidney dysfunction | High LDL cholesterol | Unsafe sex |
| **10** | Alcohol use | Occupational risks | Occupational risks | High body-mass index | Alcohol use | Occupational risks | Alcohol use | Child and maternal malnutrition | Kidney dysfunction | Alcohol use | Alcohol use | Alcohol use |

Abbreviations: DALY, disability adjusted life year; SDI: social-demographic index.

# Supplementary Table 3 Age-standardized death attributable top 10 grade 2 level risk factors in different SDI regions of 1990 and 2019

| Ranks | **1990 global** | **1990 high SDI** | **1990 high-middle SDI** | **1990 middle SDI** | **1990 low-middle SDI** | **1990 low SDI** | **2019 global** | **2019 high SDI** | **2019 high-middle SDI** | **2019 middle SDI** | **2019 low-middle SDI** | **2019 low SDI** |
| --- | --- | --- | --- | --- | --- | --- | --- | --- | --- | --- | --- | --- |
| 1 | High systolic blood pressure | High systolic blood pressure | High systolic blood pressure | Air pollution | Air pollution | Air pollution | High systolic blood pressure | High systolic blood pressure | High systolic blood pressure | High systolic blood pressure | High systolic blood pressure | Air pollution |
| 2 | Tobacco | Tobacco | Dietary risks | High systolic blood pressure | Tobacco | Unsafe water, sanitation, and handwashing | Tobacco | Tobacco | Tobacco | Tobacco | Air pollution | High systolic blood pressure |
| 3 | Air pollution | Dietary risks | Tobacco | Tobacco | High systolic blood pressure | Child and maternal malnutrition | Dietary risks | Dietary risks | Dietary risks | Dietary risks | Tobacco | Dietary risks |
| 4 | Dietary risks | High LDL cholesterol | Air pollution | Dietary risks | Unsafe water, sanitation, and handwashing | High systolic blood pressure | Air pollution | High fasting plasma glucose | High fasting plasma glucose | Air pollution | Dietary risks | High fasting plasma glucose |
| 5 | Child and maternal malnutrition | High fasting plasma glucose | High LDL cholesterol | High fasting plasma glucose | Child and maternal malnutrition | Tobacco | High fasting plasma glucose | High LDL cholesterol | High LDL cholesterol | High fasting plasma glucose | High fasting plasma glucose | Unsafe water, sanitation, and handwashing |
| 6 | High LDL cholesterol | High body-mass index | High fasting plasma glucose | Child and maternal malnutrition | Dietary risks | Dietary risks | High body-mass index | High body-mass index | High body-mass index | High body-mass index | High body-mass index | Tobacco |
| 7 | High fasting plasma glucose | Air pollution | High body-mass index | High LDL cholesterol | High fasting plasma glucose | High fasting plasma glucose | High LDL cholesterol | Air pollution | Air pollution | High LDL cholesterol | High LDL cholesterol | Child and maternal malnutrition |
| 8 | Unsafe water, sanitation, and handwashing | Alcohol use | Non-optimal temperature | High body-mass index | High LDL cholesterol | High LDL cholesterol | Child and maternal malnutrition | Kidney dysfunction | Kidney dysfunction | Kidney dysfunction | Child and maternal malnutrition | High body-mass index |
| 9 | High body-mass index | Kidney dysfunction | Alcohol use | Kidney dysfunction | Kidney dysfunction | Alcohol use | Kidney dysfunction | Alcohol use | Alcohol use | Non-optimal temperature | Unsafe water, sanitation, and handwashing | High LDL cholesterol |
| 10 | Kidney dysfunction | Non-optimal temperature | Kidney dysfunction | Non-optimal temperature | Occupational risks | Unsafe sex | Alcohol use | Non-optimal temperature | Non-optimal temperature | Alcohol use | Kidney dysfunction | Kidney dysfunction |

Abbreviation: SDI: social-demographic index.

# Supplementary Table 4 Deaths and DALYs attributable to high LDL-C of all countries and territories

| **Country** | **1990 Death No. (95%UI)** | **1990 age-standardized Death rate per 100,000 No. (95%UI)** | **2019 Death No. (95%UI)** | **2019 age-standardized Death rate per 100,000 No. (95%UI)** | **1990-2019 EAPC % (95%CI)** | **percentage change in age-standardized death rates, 1990-2019** | **1990 DALY No. (95%UI)** | **1990 age-standardized DALY rate per 100,000 No. (95%UI)** | **2019 DALY No. (95%UI)** | **2019 age-standardized DALY rate per 100,000 No. (95%UI)** | **1990-2019 EAPC % (95%CI)** | **percentage change in age-standardized DALY rates, 1990-2019** |
| --- | --- | --- | --- | --- | --- | --- | --- | --- | --- | --- | --- | --- |
| **Afghanistan** | 12704.8 (9330.8, 16528.8) | 192.1 (140.1, 250.5) | 19746 (14548, 25964.2) | 160.2 (116.1, 211.5) | -0.8% (-0.9%, -0.6%) | -16.6% (-36.7%, 4.1%) | 368120.2 (271553.9, 485460.2) | 4947.8 (3651.4, 6468.1) | 638947.8 (473406, 867979.8) | 3927.4 (2902.5, 5166) | -1% (-1.1%, -0.8%) | -20.6% (-41.1%, 3%) |
| **Albania** | 1445.8 (1114.5, 1783.3) | 81 (60, 103.5) | 2790.6 (1861.2, 3949.2) | 68.6 (45.2, 97.1) | -0.2% (-0.4%, 0%) | -15.3% (-34.6%, 7.5%) | 32726.2 (27549.8, 38232.8) | 1573 (1280.4, 1883.7) | 51859 (37031.5, 71058.1) | 1290.5 (922, 1760.4) | -0.4% (-0.6%, -0.1%) | -18% (-36.8%, 6.1%) |
| **Algeria** | 14227.5 (10384.6, 18837.5) | 165.9 (113.9, 228.4) | 27534 (18664.4, 38068.3) | 105.3 (67.1, 149) | -1.6% (-1.7%, -1.5%) | -36.5% (-49.3%, -22.5%) | 388053.5 (295265.5, 499620.4) | 3265.4 (2429.3, 4293.6) | 641373.1 (467684, 846275.9) | 1914 (1362.6, 2580.8) | -2% (-2.1%, -1.9%) | -41.4% (-54.7%, -25.5%) |
| **American Samoa** | 15 (12.3, 18.3) | 70.2 (54.2, 89.2) | 31.4 (23.9, 39.5) | 69.5 (51.8, 89.2) | 0% (-0.1%, 0%) | -0.9% (-17.8%, 18.3%) | 486.9 (405.7, 574.1) | 1806.2 (1482.3, 2180) | 901.9 (698.6, 1129.5) | 1775.5 (1369.1, 2237.3) | -0.1% (-0.2%, 0%) | -1.7% (-20.3%, 19.5%) |
| **Andorra** | 18.7 (13.3, 26.8) | 45.5 (30.9, 65.6) | 41.1 (26, 59.2) | 25.9 (16.8, 37) | -2.1% (-2.3%, -1.9%) | -43.1% (-59%, -25.8%) | 429.4 (317.3, 580) | 829.3 (611.5, 1130.6) | 672.2 (475.6, 904.5) | 467.7 (331.5, 627.9) | -2.2% (-2.4%, -2%) | -43.6% (-60.3%, -24.4%) |
| **Angola** | 1584.9 (1133.3, 2151) | 46.8 (32, 66.3) | 3963.9 (2781.9, 5457.8) | 44 (28.6, 62.8) | -0.4% (-0.5%, -0.3%) | -6% (-31.5%, 26.1%) | 48928.5 (35416.2, 65978.9) | 1105.2 (792.6, 1493) | 115912.5 (81987.5, 155901) | 943.7 (663.9, 1292.7) | -0.8% (-0.9%, -0.7%) | -14.6% (-37.8%, 17.9%) |
| **Antigua and Barbuda** | 33.4 (24.2, 44.7) | 60.9 (45, 79.9) | 36.6 (26.4, 48.4) | 41.1 (28.7, 56) | -1.6% (-1.8%, -1.4%) | -32.4% (-42.6%, -23.7%) | 661 (527.8, 824) | 1279.9 (1038.8, 1573.1) | 762.6 (588.1, 961.7) | 763.2 (580.1, 969.6) | -2% (-2.2%, -1.8%) | -40.4% (-49.6%, -31.6%) |
| **Argentina** | 23841.6 (18176.1, 30295.7) | 81.9 (61.1, 106) | 22649.2 (16748.2, 29551.5) | 41.4 (30.9, 53.6) | -2.3% (-2.6%, -2.1%) | -49.4% (-52.4%, -46.5%) | 508686.2 (421570.8, 607799) | 1625.5 (1337.1, 1950.8) | 430443 (353853.8, 522031.6) | 817.7 (680.6, 986.1) | -2.4% (-2.6%, -2.2%) | -49.7% (-52.6%, -46.8%) |
| **Armenia** | 2741.1 (2043.7, 3544.1) | 121.2 (85.4, 162.5) | 3455 (2340.6, 4722.4) | 88 (59.3, 121.2) | -1.8% (-2.1%, -1.6%) | -27.4% (-37.6%, -16.8%) | 61841.6 (49847.5, 75565.5) | 2315.9 (1812.7, 2903.8) | 67153.7 (50091.8, 86306.1) | 1670.8 (1249.8, 2151.1) | -1.7% (-1.9%, -1.5%) | -27.9% (-38.8%, -16%) |
| **Australia** | 17428.5 (13104.9, 22198.4) | 94.9 (70.4, 122.1) | 14012.8 (9489.6, 19216.5) | 30 (21.1, 40.1) | -4.3% (-4.5%, -4%) | -68.4% (-71.5%, -65.6%) | 333408.4 (272183.8, 399792.4) | 1757.9 (1443.6, 2100.7) | 212392.7 (163122.5, 265910.4) | 525.2 (420.9, 639) | -4.3% (-4.6%, -4.1%) | -70.1% (-72.1%, -67.8%) |
| **Austria** | 11870.2 (8712.9, 15757.4) | 99 (73.6, 129.8) | 9409.1 (6279.5, 12872.4) | 44 (30.6, 58.5) | -3.3% (-3.5%, -3.1%) | -55.6% (-61.2%, -50.8%) | 207451.2 (166345, 255247.1) | 1806 (1482.3, 2185.5) | 130457 (98299.1, 166438.8) | 716.9 (572.8, 880.9) | -3.7% (-3.9%, -3.5%) | -60.3% (-63.5%, -57.3%) |
| **Azerbaijan** | 6773.3 (5302, 8353.1) | 142.5 (108.1, 180.1) | 11917.6 (8762.9, 15278.1) | 181.4 (122.4, 248.6) | 0.6% (0.4%, 0.8%) | 27.3% (5%, 50%) | 165536.3 (137826.1, 193778) | 3162.5 (2563.2, 3773) | 280505 (218453.2, 351332.2) | 3156.6 (2355.3, 4027.8) | -0.4% (-0.6%, -0.2%) | -0.2% (-16.6%, 18.5%) |
| **Bahamas** | 92.4 (73.3, 116.1) | 65.2 (49.8, 83.9) | 163.9 (120.1, 216.8) | 44.6 (31.9, 59.9) | -1.5% (-1.7%, -1.3%) | -31.5% (-43.9%, -17%) | 2439.8 (2028.8, 2932) | 1477.1 (1213.5, 1807) | 4218.4 (3213.7, 5408.1) | 1022 (776.7, 1319.5) | -1.4% (-1.6%, -1.2%) | -30.8% (-44.1%, -14.1%) |
| **Bahrain** | 318.8 (262.4, 378.4) | 222.4 (168.5, 280.9) | 496.6 (366.8, 629.6) | 76.6 (50.6, 106.1) | -4.1% (-4.3%, -3.8%) | -65.6% (-73%, -56.9%) | 9805.8 (8223.8, 11465.5) | 4582.3 (3715, 5486.8) | 15402.8 (11913.5, 19278) | 1389 (1031, 1802.7) | -4.8% (-5%, -4.5%) | -69.7% (-76.2%, -61.9%) |
| **Bangladesh** | 18303.4 (13395.8, 24316.2) | 41.7 (29, 57.9) | 48742.8 (32361.9, 70383.3) | 40.6 (26.3, 60.3) | 0.3% (0%, 0.7%) | -2.5% (-24.4%, 23.4%) | 546625.4 (416018.4, 692630.2) | 1026.1 (760.1, 1325) | 1285810 (898240.5, 1746949.4) | 946.4 (656, 1304.2) | 0.2% (0%, 0.5%) | -7.8% (-30.2%, 20.2%) |
| **Barbados** | 185.2 (131, 254.3) | 63.5 (46.2, 85.1) | 176.3 (122.2, 245.9) | 36.4 (25.2, 50.9) | -2.2% (-2.5%, -2%) | -42.6% (-50.9%, -34.4%) | 3379.2 (2642.2, 4255.5) | 1229 (992.4, 1509) | 3352.8 (2522.2, 4412.9) | 709.7 (537.5, 926.8) | -2.2% (-2.5%, -2%) | -42.2% (-51.3%, -32.6%) |
| **Belarus** | 19471.7 (14432.5, 24977.9) | 159.4 (117.2, 206.8) | 24804.2 (16566.8, 34611.2) | 152.1 (102.6, 210.7) | -0.5% (-0.8%, -0.1%) | -4.6% (-22.9%, 17.1%) | 388877.6 (314869.1, 467225.8) | 3075.5 (2481.9, 3699.9) | 454146 (326885.4, 607721.5) | 2902.1 (2105.9, 3855.6) | -0.6% (-1.1%, -0.1%) | -5.6% (-25.3%, 17.7%) |
| **Belgium** | 12993.7 (9637.5, 16845.6) | 84.9 (63.8, 109.2) | 7562 (5070.7, 10558.6) | 27.6 (19.7, 37) | -4.2% (-4.4%, -4%) | -67.5% (-70%, -64.9%) | 234670.1 (190029, 285937.6) | 1589.3 (1313.6, 1901.7) | 112781.5 (85612.5, 144969.1) | 501.7 (400.6, 617.7) | -4.3% (-4.5%, -4.1%) | -68.4% (-70.4%, -66.4%) |
| **Belize** | 52 (38.7, 67.9) | 58.2 (42.8, 77) | 100.5 (75.9, 129.5) | 38.7 (28.4, 51.2) | -2% (-2.4%, -1.5%) | -33.5% (-41.9%, -23.5%) | 1129.5 (900.4, 1395) | 1198.9 (952.5, 1483.7) | 2525.6 (2039.1, 3120) | 848.5 (669.3, 1054.3) | -1.8% (-2.3%, -1.4%) | -29.2% (-39%, -17.8%) |
| **Benin** | 788.6 (559.6, 1080.5) | 44.5 (30.5, 62.5) | 1761.1 (1209.6, 2460.5) | 42.4 (28.4, 60.5) | -0.1% (-0.2%, -0.1%) | -4.7% (-22.5%, 19.4%) | 19235.9 (14282, 25382) | 943.7 (690.1, 1266) | 46388.6 (32655.9, 63395.7) | 890.4 (621.6, 1226.5) | -0.2% (-0.3%, -0.1%) | -5.7% (-25.6%, 21.2%) |
| **Bermuda** | 61.4 (46.8, 77.3) | 107.6 (79.7, 139.1) | 49.1 (33, 68) | 35.6 (24.4, 48.8) | -3.9% (-4.3%, -3.5%) | -66.9% (-72.2%, -60.6%) | 1317.9 (1075.5, 1581) | 2123.9 (1723.2, 2559) | 829.8 (616.4, 1094.5) | 665.3 (501, 862) | -4.1% (-4.5%, -3.7%) | -68.7% (-73.6%, -62.7%) |
| **Bhutan** | 132.1 (90.5, 181.1) | 59.3 (40.5, 82.9) | 335.6 (236.6, 451.5) | 66.1 (45.8, 89.4) | 0.4% (0.4%, 0.4%) | 11.5% (-19.2%, 50.9%) | 4117 (2809.5, 5606.9) | 1438.3 (999.6, 1958.8) | 8380.2 (6018.6, 10956.5) | 1423.4 (1027.8, 1881.9) | -0.1% (-0.1%, 0%) | -1% (-29.2%, 42.7%) |
| **Bolivia (Plurinational State of)** | 1599.3 (1102.6, 2225.4) | 59 (40.2, 83.9) | 3393.8 (2224.6, 4927.6) | 45.8 (29.1, 67.4) | -0.9% (-1.2%, -0.7%) | -22.5% (-39.9%, -0.7%) | 40255.8 (27621.8, 55400.1) | 1213.4 (838.4, 1661.3) | 74813.2 (49848.8, 105684.8) | 861.1 (575.3, 1230.9) | -1.2% (-1.5%, -1%) | -29% (-46.5%, -7.6%) |
| **Bosnia and Herzegovina** | 4334.3 (3439.6, 5411.2) | 131.3 (98, 172.4) | 5101.9 (3408.7, 7256) | 94.4 (62.7, 134.8) | -1.5% (-1.7%, -1.3%) | -28.1% (-42.5%, -13.4%) | 104453.1 (88488.6, 124169.7) | 2579.8 (2105.4, 3157.2) | 93917.4 (67682.1, 127224.6) | 1683.4 (1217.8, 2278.1) | -1.9% (-2.1%, -1.8%) | -34.8% (-47.7%, -20.4%) |
| **Botswana** | 205 (135.5, 295.5) | 43.5 (27.4, 65.2) | 559.2 (352.6, 815.1) | 48.4 (30, 72.5) | 0% (-0.3%, 0.4%) | 11.1% (-17.8%, 50.8%) | 5693.1 (3836.1, 8023.5) | 964.1 (654.1, 1383.5) | 15976.1 (10228.5, 22978.7) | 1057.5 (674.8, 1531.7) | -0.1% (-0.6%, 0.4%) | 9.7% (-22.1%, 52%) |
| **Brazil** | 68327.1 (55096.6, 83767.8) | 88.6 (67.8, 114.8) | 99375.2 (78038.6, 126142.7) | 43.1 (33.4, 55.9) | -2.4% (-2.5%, -2.2%) | -51.3% (-53.8%, -48.6%) | 1821799.5 (1548456.3, 2139062.9) | 1940.1 (1614.4, 2322.9) | 2363140.8 (1985655.3, 2781317.9) | 981.3 (817.1, 1162.4) | -2.3% (-2.4%, -2.2%) | -49.4% (-52%, -46.8%) |
| **Brunei Darussalam** | 81.5 (65.2, 100.2) | 104 (77.5, 136.3) | 149.3 (120.5, 180.7) | 68.3 (49.6, 88.7) | -1.4% (-1.5%, -1.2%) | -34.3% (-43.5%, -22.8%) | 2488.5 (2056.5, 2983.2) | 2163.4 (1727.5, 2688.5) | 4432.7 (3692.3, 5285.3) | 1340.7 (1066.8, 1649.3) | -1.6% (-1.8%, -1.4%) | -38% (-46.6%, -27.4%) |
| **Bulgaria** | 18843.6 (14449.7, 24091.7) | 197.2 (146.1, 258.7) | 19141.2 (13094.4, 26843) | 136.7 (94.4, 189.5) | -2.1% (-2.5%, -1.8%) | -30.7% (-43%, -16%) | 398313.9 (326918.6, 482934.8) | 3565 (2904.1, 4354) | 340337.7 (246331.2, 456535) | 2606 (1933.2, 3424.1) | -1.9% (-2.3%, -1.6%) | -26.9% (-40.9%, -10.2%) |
| **Burkina Faso** | 806.1 (516, 1203.5) | 22.2 (13.6, 33.9) | 2051.3 (1282.3, 2984.1) | 25.8 (15.7, 38.5) | 0.7% (0.6%, 0.9%) | 16.4% (-4.8%, 42.7%) | 22532 (14855.9, 32669.3) | 498.1 (323.6, 731.2) | 56752.8 (36699.6, 80737.8) | 565.6 (362.6, 812.9) | 0.5% (0.4%, 0.6%) | 13.6% (-10.3%, 43.3%) |
| **Burundi** | 949.6 (643.3, 1374.9) | 45.8 (30.1, 67.9) | 1512.2 (996, 2151.3) | 38.9 (24.4, 57.2) | -0.8% (-0.9%, -0.7%) | -14.9% (-38%, 17.2%) | 27072.4 (18704.7, 37873) | 1085.9 (744.4, 1545.2) | 45011.2 (30317.5, 62640.6) | 872.6 (584, 1234.7) | -1.1% (-1.2%, -1%) | -19.6% (-43.4%, 14.1%) |
| **Cabo Verde** | 102.1 (69.4, 140.7) | 43.5 (30, 59.2) | 217.3 (147.6, 298) | 51.1 (34.7, 69.9) | 0% (-0.4%, 0.3%) | 17.4% (1%, 35.2%) | 1965.3 (1480.2, 2494.9) | 872.9 (669.5, 1097.6) | 4314.2 (3219.3, 5537.9) | 971.8 (717.6, 1245.1) | -0.1% (-0.4%, 0.1%) | 11.3% (-4.9%, 31.5%) |
| **Cambodia** | 2311 (1770.4, 3149.2) | 58.6 (43.4, 80.5) | 5567.6 (4007.9, 7556.9) | 55.9 (38, 78.3) | -0.3% (-0.4%, -0.2%) | -4.7% (-28.2%, 16.4%) | 68247.6 (53254.2, 89005.9) | 1388.5 (1079, 1852.8) | 143509.2 (107756.2, 186259.6) | 1172.7 (859.4, 1569.3) | -0.7% (-0.8%, -0.6%) | -15.5% (-36%, 7.3%) |
| **Cameroon** | 642.7 (382.1, 1012.9) | 17.7 (10.1, 29) | 2285.1 (1360.4, 3545.5) | 22.6 (13.1, 34.8) | 1% (0.8%, 1.2%) | 27.4% (-3.3%, 72.6%) | 17157.2 (10389, 26266.2) | 369.8 (220.4, 570.5) | 64599.3 (39046, 98420.9) | 488.4 (293, 748.7) | 1.1% (0.9%, 1.4%) | 32.1% (-3.2%, 83.7%) |
| **Canada** | 25255.4 (18856.8, 32371.5) | 81.1 (60.5, 104.3) | 22409.3 (15375.8, 30562.2) | 29.9 (21.2, 39.8) | -4% (-4.3%, -3.8%) | -63.1% (-65.6%, -60.5%) | 495693.2 (404537.8, 593278.7) | 1574.2 (1293.9, 1876.2) | 373965.5 (292646, 467228) | 572.6 (462, 697) | -3.9% (-4.2%, -3.6%) | -63.6% (-65.6%, -61.7%) |
| **Central African Republic** | 597.8 (437.4, 827.2) | 57.5 (40.2, 82.2) | 1095 (748.1, 1551.5) | 56.8 (37.1, 82.6) | 0.1% (0%, 0.1%) | -1.2% (-24.4%, 28.2%) | 18441.4 (13393.3, 25663.4) | 1405.3 (1035.9, 1935.1) | 34700.3 (24146.9, 48549) | 1357.8 (929.1, 1918.9) | 0% (-0.1%, 0%) | -3.4% (-27.7%, 29.7%) |
| **Chad** | 926.2 (646.8, 1320.6) | 38.3 (25.7, 56.3) | 2065.7 (1443.8, 2825.1) | 43.4 (29.3, 62.2) | 0.6% (0.5%, 0.6%) | 13.4% (-7.5%, 41.4%) | 22585.5 (16303.3, 31091.2) | 803.8 (579.1, 1111.1) | 55820 (39810.6, 75633.4) | 930.5 (657.7, 1263.8) | 0.6% (0.5%, 0.7%) | 15.8% (-7.5%, 48.3%) |
| **Chile** | 5257.3 (3908, 6825.4) | 63.1 (45.4, 84) | 6462.2 (4744.8, 8722.4) | 27.2 (19.9, 36.8) | -2.6% (-2.8%, -2.5%) | -56.9% (-60.6%, -52.4%) | 107106.3 (86650.9, 130345.6) | 1114.8 (886.5, 1379.4) | 127343.4 (103216.6, 156365.4) | 538.4 (438.2, 659.6) | -2.2% (-2.3%, -2.1%) | -51.7% (-55%, -48.1%) |
| **China** | 317059.9 (244157.8, 413835.6) | 48.9 (35, 68.3) | 915983.1 (647993.1, 1239381.7) | 55.8 (37.6, 77.3) | 1% (0.7%, 1.2%) | 14.2% (-4.5%, 35.2%) | 8889099.2 (7139095.2, 11088753.2) | 1046.3 (826.4, 1346.2) | 19813961.9 (15205317, 25139359) | 1052.2 (800.2, 1344.7) | 0.4% (0.2%, 0.6%) | 0.6% (-15.9%, 20.4%) |
| **Colombia** | 11033.5 (8657.7, 13632.3) | 70.7 (52.6, 90.4) | 19656 (13325.4, 27944) | 35.4 (24.4, 50) | -2.5% (-2.6%, -2.3%) | -49.9% (-60.4%, -37.5%) | 275633.8 (234136.2, 323057.5) | 1489 (1225, 1784.5) | 378878.7 (277391.7, 510668.9) | 708.1 (520, 952) | -2.7% (-2.9%, -2.5%) | -52.5% (-62.3%, -39.5%) |
| **Comoros** | 89.8 (53.5, 127.6) | 48 (29.2, 69) | 203.2 (133.6, 284.2) | 47.1 (29.9, 67.7) | -0.2% (-0.3%, 0%) | -1.7% (-26.2%, 47.3%) | 2321 (1271, 3261.8) | 1041.4 (602.4, 1463.2) | 5004.6 (3408.2, 6828.5) | 999.6 (681.5, 1371.3) | -0.3% (-0.4%, -0.1%) | -4% (-31.3%, 64.8%) |
| **Congo** | 630.2 (459.3, 849.4) | 69 (47.2, 95) | 1251.7 (839.2, 1750.6) | 57.8 (36.4, 84.5) | -0.7% (-0.8%, -0.6%) | -16.2% (-35.1%, 5.8%) | 18532.6 (13758.1, 24470.4) | 1624.8 (1189.6, 2153.7) | 35433.5 (23871.9, 49545.6) | 1233.2 (839.3, 1709.7) | -1.1% (-1.3%, -1%) | -24.1% (-42.6%, -1.7%) |
| **Cook Islands** | 8 (6.2, 10.3) | 66.7 (50.9, 88.3) | 12.1 (8.9, 15.7) | 51.9 (38.3, 67.6) | -0.8% (-1%, -0.6%) | -22.2% (-39.2%, -2%) | 237 (187.5, 301.6) | 1734.6 (1374.3, 2207.1) | 307.9 (238.9, 389.2) | 1330.3 (1030.1, 1695.2) | -0.8% (-1%, -0.6%) | -23.3% (-41.1%, -0.6%) |
| **Costa Rica** | 964.4 (724.5, 1232.2) | 59 (43.4, 76.9) | 1840.8 (1294.6, 2560.6) | 35.2 (24.5, 48.9) | -2.2% (-2.5%, -1.9%) | -40.3% (-52%, -24.9%) | 20950.1 (17317.6, 25082.4) | 1166.8 (947.4, 1417.4) | 37792.3 (27816.8, 50284.2) | 724.8 (532.3, 965.1) | -2% (-2.2%, -1.7%) | -37.9% (-50.6%, -21.1%) |
| **Croatia** | 7907.1 (5924.8, 10195.2) | 143.6 (104.2, 188.9) | 6857.6 (4447.1, 9795.7) | 75.4 (50, 106) | -2.1% (-2.2%, -2%) | -47.5% (-57.3%, -36.9%) | 156732.9 (127334.2, 190557.4) | 2566.1 (2063.7, 3142.4) | 105250.3 (75027.6, 142582.6) | 1239.5 (899.5, 1649) | -2.5% (-2.6%, -2.4%) | -51.7% (-61%, -41.2%) |
| **Cuba** | 8355.6 (6097.2, 10717.2) | 86.4 (62.9, 112) | 9590.6 (6393.4, 13549.8) | 47.8 (32.1, 66.7) | -2.4% (-2.6%, -2.1%) | -44.6% (-54.2%, -34%) | 176422.8 (143135.7, 212236) | 1737.5 (1408.3, 2093.1) | 176874.9 (130525.4, 232779.3) | 946.9 (708.8, 1238.9) | -2.4% (-2.7%, -2.1%) | -45.5% (-55%, -34.6%) |
| **Cyprus** | 749.9 (582.6, 958.9) | 126.5 (92, 167.4) | 864.1 (604.2, 1163.2) | 52.6 (36, 71.7) | -3.6% (-3.8%, -3.4%) | -58.5% (-65%, -51.1%) | 15537.7 (12781.5, 18764) | 2167.6 (1774.3, 2665.5) | 16370.3 (12763.4, 20388.4) | 920 (719.4, 1144) | -3.5% (-3.7%, -3.3%) | -57.6% (-63.9%, -51%) |
| **Czechia** | 26414 (20487.7, 33412.3) | 203.4 (156.5, 259.3) | 16348.9 (10942.2, 22300.3) | 75.6 (51.1, 102.5) | -3.6% (-3.7%, -3.5%) | -62.8% (-70.5%, -55%) | 526986.7 (437078.3, 631361.9) | 3943.2 (3289.6, 4705.9) | 253452.6 (186063.2, 332959.8) | 1256 (942.7, 1632.6) | -4.1% (-4.3%, -4%) | -68.2% (-73.8%, -61.8%) |
| **Côte d'Ivoire** | 1601.5 (1179.8, 2144.9) | 49.9 (34.5, 70.4) | 4002.7 (2740, 5474.7) | 45.6 (30.2, 64) | -0.3% (-0.4%, -0.2%) | -8.8% (-25.4%, 13%) | 48641.2 (36738.3, 64884.1) | 1073 (797.4, 1435.4) | 116150.4 (80326.6, 158011.2) | 969.1 (673.5, 1316) | -0.4% (-0.6%, -0.2%) | -9.7% (-29.1%, 16%) |
| **Democratic People's Republic of Korea** | 8004.9 (5809.7, 10616.4) | 60.4 (41.1, 84.5) | 20170.2 (14794.5, 26969.5) | 69.5 (50.1, 94.2) | 0.6% (0.4%, 0.8%) | 15.2% (-7%, 47%) | 231457.8 (171082.3, 301756) | 1380.6 (1007.8, 1808.7) | 498888.6 (381374.8, 646423.1) | 1562.1 (1195.6, 2031.4) | 0.5% (0.3%, 0.7%) | 13.2% (-14.1%, 46.8%) |
| **Democratic Republic of the Congo** | 6148.4 (4239.3, 8539) | 48.2 (31.6, 70.1) | 13181 (8651.6, 18920.2) | 44.1 (27.3, 65.8) | -0.4% (-0.4%, -0.4%) | -8.5% (-29.4%, 16.1%) | 178747.9 (126429.3, 245933.6) | 1068.5 (743.5, 1474) | 366915 (249720, 515660.6) | 946.3 (630.5, 1341.1) | -0.5% (-0.6%, -0.5%) | -11.4% (-32.9%, 15.2%) |
| **Denmark** | 9864.5 (7269.6, 12748.5) | 118.3 (89.2, 150.6) | 3793.1 (2588.8, 5252.3) | 30 (21.1, 40.8) | -5.4% (-5.8%, -5.1%) | -74.6% (-77.5%, -71.8%) | 169966 (136936.2, 205277) | 2185.3 (1819.9, 2584) | 55812.4 (41955.2, 71372.7) | 502.5 (393, 623.4) | -5.7% (-6%, -5.3%) | -77% (-79%, -74.9%) |
| **Djibouti** | 37.8 (25.4, 53) | 34.4 (21.8, 51.6) | 198.5 (121.3, 289) | 41.7 (25.5, 62.1) | 0.7% (0.6%, 0.8%) | 21.1% (-10.5%, 62.9%) | 1224.8 (827.4, 1669.7) | 750.8 (509.2, 1061.8) | 6033.4 (3745.4, 8877.6) | 894.1 (559.8, 1294) | 0.6% (0.4%, 0.7%) | 19.1% (-15.1%, 66.9%) |
| **Dominica** | 40.7 (28.3, 56) | 57.2 (40.5, 77.9) | 35.3 (23.8, 49.3) | 38.9 (26.2, 54.3) | -1.4% (-1.6%, -1.2%) | -31.9% (-43.6%, -18.1%) | 762.4 (577, 994.4) | 1126.5 (874.9, 1440.3) | 671.2 (489.7, 895.5) | 753.9 (550.9, 1004.6) | -1.5% (-1.7%, -1.3%) | -33.1% (-45.6%, -18%) |
| **Dominican Republic** | 2119.4 (1665.1, 2663) | 64.2 (47.7, 83.2) | 7467.2 (5326.6, 10179.4) | 83.6 (58.9, 114.7) | 1.8% (1.4%, 2.1%) | 30.3% (1.5%, 65.3%) | 56865.6 (46840.7, 67825.8) | 1402.4 (1136.3, 1687.5) | 183199.2 (134466.8, 247121.1) | 1891.9 (1383.1, 2544) | 1.6% (1.4%, 1.9%) | 34.9% (0.8%, 74.7%) |
| **Ecuador** | 2218.4 (1702.6, 2799.4) | 46.9 (34.5, 61.1) | 5116.5 (3517.3, 7111.1) | 38.4 (25.6, 53.9) | -0.5% (-0.8%, -0.2%) | -18.1% (-33.9%, 2.4%) | 55094.4 (45942.7, 64717.9) | 965.1 (782.8, 1167) | 115854.6 (84803.7, 153207.9) | 765.5 (556.2, 1022.1) | -0.8% (-1%, -0.6%) | -20.7% (-36.9%, 1%) |
| **Egypt** | 43267.2 (34466.5, 53629.6) | 170.8 (126.7, 219.3) | 88183.2 (63245.2, 119918.5) | 159.6 (111.3, 220.2) | -0.1% (-0.2%, 0%) | -6.5% (-28.6%, 17.3%) | 1211661.5 (1003482.6, 1475836) | 3826.3 (3103.4, 4725.7) | 2451127.6 (1757350, 3305648.5) | 3560.6 (2583.8, 4814.1) | -0.1% (-0.2%, 0%) | -6.9% (-29.5%, 19.1%) |
| **El Salvador** | 1702.9 (1330.2, 2133.7) | 58.7 (44.9, 74.5) | 3057.8 (2036.5, 4319.6) | 47.7 (32.5, 66.5) | -0.8% (-1%, -0.6%) | -18.7% (-37.4%, 3.8%) | 42165 (35376.6, 48984.6) | 1332.7 (1110.8, 1566.1) | 59298.6 (42381.3, 81149.7) | 985.7 (705.3, 1347) | -1.1% (-1.4%, -0.9%) | -26% (-43.7%, -4.6%) |
| **Equatorial Guinea** | 94.8 (62.6, 134.2) | 53.8 (34.5, 78.9) | 146.9 (92.1, 224) | 38.7 (22.3, 59) | -1.5% (-1.8%, -1.3%) | -28% (-54%, 5.2%) | 2783.7 (1880, 3787.1) | 1298.4 (875.7, 1817.4) | 3803.7 (2429.2, 5663.2) | 754.8 (478, 1133.3) | -2.3% (-2.7%, -2%) | -41.9% (-61.9%, -13.4%) |
| **Eritrea** | 301.1 (201.2, 452.6) | 33.9 (21.8, 51.6) | 940.8 (655.9, 1332.5) | 42 (27.9, 61) | 0.7% (0.6%, 0.8%) | 23.9% (-11.9%, 71.1%) | 10206 (6836.2, 15141.3) | 841.4 (566.1, 1245.8) | 29678.5 (20390.8, 41949.5) | 945.8 (664.8, 1324) | 0.4% (0.2%, 0.5%) | 12.4% (-18.9%, 55.3%) |
| **Estonia** | 3988.7 (3047.3, 5051.6) | 208.1 (157, 266.3) | 2314.1 (1428.7, 3413.4) | 76.8 (49.5, 111.9) | -4.4% (-4.8%, -4%) | -63.1% (-71.1%, -50.5%) | 77702.7 (63611.6, 93569.1) | 3890.2 (3188.7, 4669.9) | 34359.3 (23625.8, 48257.5) | 1305.3 (924.3, 1789) | -4.9% (-5.3%, -4.4%) | -66.5% (-73.7%, -56.3%) |
| **Eswatini** | 87.5 (58.4, 125.9) | 35.6 (22.9, 53.2) | 208.6 (132.1, 312.8) | 44.6 (27.6, 67.8) | 1.2% (0.8%, 1.5%) | 25.1% (-9.4%, 72.2%) | 2416.3 (1675.4, 3319.8) | 776.9 (532.2, 1095.8) | 5931.6 (3767.3, 8633.1) | 971.5 (626.8, 1435.9) | 1.2% (0.8%, 1.7%) | 25.1% (-10.8%, 78.4%) |
| **Ethiopia** | 5594.5 (3782.3, 8077.1) | 30.6 (20.2, 45.4) | 8285.4 (5141.3, 12037.8) | 23.4 (13.8, 35.2) | -1.2% (-1.3%, -1.1%) | -23.6% (-55.1%, 7.3%) | 181366.9 (124577.4, 259114.8) | 785.2 (537.4, 1125.8) | 223103.1 (144703.3, 313430.6) | 494.9 (316.1, 706.5) | -1.9% (-2.1%, -1.8%) | -37% (-62.9%, -10.4%) |
| **Fiji** | 523.7 (419.2, 658.1) | 144.8 (110.6, 186.1) | 865.5 (649, 1136.8) | 126.6 (93, 165.5) | -0.7% (-0.8%, -0.6%) | -12.6% (-31.9%, 13.6%) | 17497.5 (14151.4, 21612.7) | 3843.3 (3076.1, 4806.1) | 26282.2 (20166.4, 34139.6) | 3181.7 (2419.4, 4129.1) | -0.8% (-0.9%, -0.7%) | -17.2% (-36.9%, 10.2%) |
| **Finland** | 9059.2 (6945.5, 11452) | 130 (100.4, 163.5) | 6983.9 (4580.4, 9684) | 48.6 (33.6, 65.7) | -3.5% (-3.7%, -3.4%) | -62.6% (-67.7%, -57.9%) | 175972.7 (145786.1, 208896.7) | 2556.8 (2150.5, 3010.3) | 96085.8 (71285.8, 124259.9) | 798.6 (624.8, 990.3) | -4.1% (-4.3%, -4%) | -68.8% (-71.8%, -65.6%) |
| **France** | 46622.2 (33573.6, 62832.2) | 53.8 (39.7, 71.7) | 37831.3 (24816.5, 53130.4) | 21.6 (15.5, 29) | -3.3% (-3.5%, -3.2%) | -59.7% (-63%, -56.7%) | 797316.6 (640851.6, 992493.8) | 984 (811.1, 1200.7) | 535425.7 (408344.7, 689455.4) | 403.4 (331.1, 486.8) | -3.2% (-3.3%, -3.1%) | -59% (-61.1%, -56.9%) |
| **Gabon** | 246.8 (174.7, 339.8) | 51.3 (34.9, 71.9) | 430.3 (292.4, 587.8) | 48.4 (31.8, 68.5) | -0.2% (-0.3%, -0.1%) | -5.7% (-25.2%, 16.5%) | 6419 (4658.1, 8524.3) | 1139.1 (817.6, 1529) | 11024.1 (7769.5, 14674.4) | 1004.2 (703.8, 1346.9) | -0.4% (-0.6%, -0.3%) | -11.8% (-32.5%, 13.3%) |
| **Gambia** | 140.8 (95, 196) | 50.6 (33.9, 72) | 503.5 (354.7, 690.2) | 60.7 (41.2, 84.5) | 0.7% (0.6%, 0.8%) | 20% (-6.6%, 53.4%) | 3803.9 (2659.9, 5225.7) | 1036.8 (710.5, 1432.5) | 12613.7 (9006.2, 16816.2) | 1257.2 (896.8, 1686.4) | 0.7% (0.5%, 0.8%) | 21.3% (-11.1%, 63.6%) |
| **Georgia** | 8929.6 (6737.4, 11375.7) | 170.8 (123.4, 223.9) | 5832.9 (4002.9, 8112) | 90.8 (63.6, 123.5) | -2.7% (-3%, -2.4%) | -46.9% (-55.4%, -36.5%) | 201601.1 (162509, 243994.9) | 3417.2 (2733.6, 4175.7) | 108325.4 (80097.4, 138613.5) | 1894.1 (1443.5, 2388) | -2.8% (-3.1%, -2.5%) | -44.6% (-53.6%, -33.9%) |
| **Germany** | 158084.6 (118218.4, 203888.4) | 122.7 (92.1, 156.9) | 94571.8 (63397.2, 130769.7) | 42.5 (30.1, 56.9) | -3.9% (-4.3%, -3.6%) | -65.3% (-68.2%, -62.2%) | 2776449 (2243386.8, 3352790.6) | 2238.1 (1848.4, 2657.9) | 1365147 (1031382, 1746640.7) | 725.7 (577.6, 889.8) | -4.1% (-4.5%, -3.8%) | -67.6% (-69.6%, -65.6%) |
| **Ghana** | 2971.1 (2219.6, 3894.4) | 59 (42, 81) | 8434.6 (6160.1, 11266.5) | 64.6 (45.3, 89.4) | 0.5% (0.4%, 0.6%) | 9.4% (-12.4%, 35.5%) | 83762.1 (63443.9, 108350.4) | 1257 (946.7, 1637.2) | 221509.7 (165118.6, 286444.4) | 1314.3 (981.3, 1732.7) | 0.4% (0.3%, 0.5%) | 4.6% (-18.5%, 32.7%) |
| **Greece** | 11597.9 (8310.9, 15778.8) | 81.8 (58.2, 112.9) | 14058.8 (9532, 19898.2) | 50.2 (36.7, 67.4) | -2.1% (-2.3%, -1.9%) | -38.7% (-44%, -33.3%) | 216074.3 (171020.4, 272763.4) | 1499.2 (1204.7, 1878.5) | 212791.3 (164889.5, 274441.1) | 982 (813.9, 1174) | -1.7% (-1.9%, -1.6%) | -34.5% (-39.2%, -29.9%) |
| **Greenland** | 31.4 (25.2, 39.2) | 110.1 (83.1, 145.1) | 28.4 (20.6, 37.3) | 47.7 (33.4, 65.1) | -3.5% (-3.7%, -3.2%) | -56.7% (-64.8%, -47.9%) | 887.8 (724.9, 1074.6) | 2361.4 (1900.9, 2944.3) | 699.4 (530.2, 895.1) | 994.8 (743.6, 1284) | -3.4% (-3.6%, -3.2%) | -57.9% (-66.3%, -48.4%) |
| **Grenada** | 69 (49.6, 94.5) | 89.4 (65.7, 118.7) | 50.9 (38.6, 65.9) | 51.6 (37.3, 68.9) | -1.9% (-2.2%, -1.6%) | -42.2% (-47.8%, -36.3%) | 1315.5 (1028.7, 1673.4) | 1903.3 (1526.7, 2385.8) | 1178 (946.8, 1461.8) | 1057.8 (846.8, 1337.6) | -1.9% (-2.2%, -1.6%) | -44.4% (-50.4%, -38.3%) |
| **Guam** | 55.4 (44.5, 68.1) | 90 (67.8, 118.1) | 155.3 (118.3, 200.5) | 82.4 (62.6, 106.5) | -0.2% (-0.5%, 0.1%) | -8.5% (-23.9%, 10.5%) | 1640.2 (1366.6, 1944.8) | 1961.9 (1594.3, 2409.4) | 4035.4 (3237, 5023.3) | 2129.8 (1712, 2644.8) | 0.4% (0.2%, 0.6%) | 8.6% (-10.4%, 31.3%) |
| **Guatemala** | 1841 (1422.8, 2313.3) | 62.2 (44.3, 82.9) | 4076.2 (2849.8, 5559.3) | 44.4 (29.6, 61.6) | -1.4% (-1.7%, -1.1%) | -28.6% (-42.4%, -12.3%) | 52001.1 (42339.2, 62611.2) | 1314.6 (1036.4, 1629.8) | 93120.2 (69235.7, 123472.1) | 815.2 (601.3, 1079.2) | -1.8% (-2.2%, -1.4%) | -38% (-50.6%, -21.9%) |
| **Guinea** | 1107.2 (796.1, 1547.8) | 39.2 (26.8, 56.4) | 2353.4 (1665.7, 3289.2) | 47.8 (32.6, 68.2) | 1.1% (0.9%, 1.2%) | 21.8% (-2.9%, 55%) | 27012.7 (20061.2, 36262.7) | 818.5 (603.2, 1113.1) | 59833.7 (43201.2, 79891.8) | 1029.2 (739.9, 1398.6) | 1.2% (1.1%, 1.4%) | 25.7% (-2.9%, 63.5%) |
| **Guinea-Bissau** | 203.4 (137.4, 291.3) | 57.8 (39.1, 82.7) | 400.4 (276.4, 538.1) | 63.9 (42.8, 88.3) | 0.6% (0.5%, 0.7%) | 10.4% (-17.9%, 50.7%) | 5923.1 (4128.9, 8376.5) | 1358.3 (940.5, 1914.3) | 12010.7 (8476.6, 15908.5) | 1456.2 (1018.8, 1943) | 0.5% (0.4%, 0.7%) | 7.2% (-23.2%, 51.4%) |
| **Guyana** | 464.1 (361.8, 577.2) | 132.4 (100, 170) | 527.9 (385, 707.3) | 92.9 (65.8, 126.3) | -1.2% (-1.4%, -1.1%) | -29.9% (-45%, -12.4%) | 12850.7 (10364, 15424.4) | 3102.6 (2454, 3804) | 13963.7 (10285.6, 18520.2) | 2100.2 (1550.2, 2798.6) | -1.3% (-1.4%, -1.2%) | -32.3% (-48.4%, -12.3%) |
| **Haiti** | 3101.2 (2334.7, 4086.2) | 108.5 (78, 147.1) | 5347.7 (3490.1, 7731.4) | 88.4 (56.7, 128.3) | -0.6% (-0.7%, -0.4%) | -18.6% (-36.6%, 4.7%) | 88071.1 (67924.2, 113571.6) | 2523.2 (1928.1, 3310.9) | 146021.6 (97091.4, 208542.4) | 1931.8 (1280.5, 2780.1) | -0.7% (-0.9%, -0.6%) | -23.4% (-42.7%, 2.2%) |
| **Honduras** | 1034.9 (777, 1421.8) | 54.5 (39.2, 77.8) | 3636 (2679.8, 4922.9) | 72.1 (51.3, 97.9) | 1.1% (0.9%, 1.3%) | 32.3% (9.5%, 63.2%) | 27561.7 (21611.2, 35761.8) | 1217.6 (946.7, 1607.2) | 80944.6 (61224.1, 106769.6) | 1354.3 (1025.3, 1788.6) | 0.5% (0.4%, 0.6%) | 11.2% (-10.7%, 42.1%) |
| **Hungary** | 23396.4 (18344.8, 29115.5) | 175.8 (136.8, 220.7) | 17396.1 (12034.8, 23726.1) | 87.1 (61.6, 117) | -2.6% (-2.8%, -2.5%) | -50.5% (-58.8%, -41%) | 514093.9 (434304.9, 606085.1) | 3712 (3160.1, 4350.5) | 296247.2 (221698.3, 385641) | 1634.3 (1249.2, 2107.2) | -3.1% (-3.2%, -3%) | -56% (-63.2%, -47.4%) |
| **Iceland** | 278.3 (207.1, 355.5) | 94.1 (71, 118.6) | 231.9 (157.1, 311.6) | 35.7 (25.4, 47.1) | -3.7% (-3.8%, -3.5%) | -62.1% (-66.4%, -58%) | 4985.4 (4073.5, 5986.3) | 1780 (1473, 2106.4) | 3488.8 (2730.4, 4342.4) | 630.5 (508.5, 768.2) | -3.9% (-4.1%, -3.7%) | -64.6% (-68%, -61%) |
| **India** | 266276.7 (212227, 331571.9) | 66.4 (50.5, 87.2) | 630093.2 (474415.3, 803890.3) | 60 (44.1, 78.8) | -0.4% (-0.5%, -0.3%) | -9.6% (-25%, 5.5%) | 8424185.5 (6905132.4, 10268463.6) | 1613 (1296.3, 1996.5) | 17679116.3 (13917214, 21933658.9) | 1461.2 (1138.4, 1822.4) | -0.3% (-0.4%, -0.2%) | -9.4% (-24.8%, 6.4%) |
| **Indonesia** | 43481.6 (33643.8, 56608.2) | 48.2 (35.4, 66.3) | 112172.8 (81574.8, 148130.8) | 60 (42, 82.6) | 0.9% (0.8%, 1%) | 24.6% (2.5%, 46.5%) | 1389954.8 (1117887.5, 1732030.6) | 1211.2 (948.5, 1553.6) | 3254385.7 (2480440.1, 4198586.5) | 1378.8 (1027, 1795.6) | 0.6% (0.6%, 0.7%) | 13.8% (-6%, 35.5%) |
| **Iran (Islamic Republic of)** | 30291.3 (24441.1, 37075.7) | 145.5 (109.3, 188.8) | 52529.6 (40049.8, 66946.6) | 79.9 (58.2, 105.1) | -2.6% (-2.9%, -2.3%) | -45.1% (-50.7%, -40.2%) | 848798.8 (712608.9, 999260.9) | 3106.6 (2528.4, 3789.1) | 1203791.4 (998464, 1437959) | 1574.5 (1268.6, 1922.4) | -2.9% (-3.1%, -2.6%) | -49.3% (-54.7%, -45.1%) |
| **Iraq** | 10601.5 (8132.8, 13602.6) | 146.7 (109.7, 191) | 24812.5 (17951.2, 32802.5) | 126.3 (89.6, 169.2) | -0.8% (-0.9%, -0.7%) | -13.9% (-31.9%, 3.9%) | 279016.5 (220719.5, 344617.5) | 3372.8 (2653.4, 4220.2) | 652285.6 (480501.9, 857796.9) | 2668.8 (1966.5, 3511.5) | -1.2% (-1.3%, -1%) | -20.9% (-38.8%, -0.4%) |
| **Ireland** | 4424.7 (3327, 5648.1) | 115.8 (87.4, 147.9) | 2737.6 (1866, 3743.4) | 35.6 (24.6, 48.2) | -4.6% (-4.9%, -4.3%) | -69.2% (-73%, -66.3%) | 86811 (70672.9, 105222.3) | 2235.7 (1838.6, 2680.2) | 44524.7 (34406.6, 55621.7) | 610.6 (478.7, 753.5) | -5% (-5.2%, -4.7%) | -72.7% (-75.2%, -70.5%) |
| **Israel** | 3985.4 (2983.5, 5061.2) | 89.2 (66.1, 114.9) | 2906.5 (1969.6, 4055.9) | 23 (16.1, 31.5) | -5.2% (-5.6%, -4.9%) | -74.2% (-77%, -71.5%) | 78096 (63809.7, 94047.5) | 1677.6 (1377.1, 2009.5) | 46346.9 (35472, 59096.2) | 406.4 (321.9, 507.2) | -5.4% (-5.8%, -5%) | -75.8% (-77.7%, -73.7%) |
| **Italy** | 60212.6 (43772.6, 81570.8) | 70.8 (51.5, 96.3) | 53139.6 (34337.2, 76683.8) | 29.3 (20.2, 40.5) | -3.3% (-3.5%, -3.1%) | -58.6% (-62.2%, -55.5%) | 1075293.8 (861292.7, 1340875.5) | 1266.5 (1023.8, 1556.6) | 712072.8 (520047.2, 938497.5) | 494.6 (390.8, 613.7) | -3.5% (-3.7%, -3.3%) | -61% (-63.3%, -58.9%) |
| **Jamaica** | 739.6 (509.8, 1064.6) | 41.2 (28.7, 58.7) | 1090.5 (720.5, 1591.5) | 33.1 (22, 47.9) | -0.8% (-1.2%, -0.4%) | -19.7% (-34.6%, -1.4%) | 13688.7 (10474.5, 18001.4) | 781.1 (606.6, 1009.3) | 20804.5 (15032.3, 27940.6) | 680.8 (493, 910.2) | -0.4% (-0.9%, 0%) | -12.8% (-29.5%, 8.8%) |
| **Japan** | 65669.4 (46796.9, 90971.3) | 43.9 (30.3, 62.5) | 75782 (47406.5, 114356.7) | 16.4 (11.6, 22.8) | -3.4% (-3.6%, -3.2%) | -62.6% (-66.3%, -59.4%) | 1240245.4 (981950.8, 1584905) | 764.3 (598.8, 992.5) | 1098334.9 (793981.6, 1529209.8) | 348.1 (283.5, 433.2) | -2.7% (-2.8%, -2.6%) | -54.5% (-57.4%, -51.4%) |
| **Jordan** | 1385.8 (1090.5, 1741) | 122.8 (90, 163.5) | 3663.2 (2786.9, 4765.9) | 68.9 (49.2, 95.2) | -2.7% (-3%, -2.4%) | -43.9% (-53.5%, -32%) | 39049.1 (31825.9, 47522.7) | 2664.6 (2109.5, 3337.8) | 101865.4 (81401.1, 127947.5) | 1438.7 (1109.1, 1845.9) | -2.8% (-3.1%, -2.5%) | -46% (-55.6%, -33.5%) |
| **Kazakhstan** | 16265.2 (12683.5, 20454.4) | 144.7 (108, 188.6) | 16201.6 (11828.4, 21761.1) | 117.7 (81.1, 164.1) | -1.5% (-2.2%, -0.8%) | -18.7% (-29.3%, -8.3%) | 387800.5 (323494.8, 461167.8) | 3020.1 (2455.7, 3658) | 354927.5 (275491.8, 455944.3) | 2125.4 (1609.2, 2753.3) | -2.2% (-3%, -1.4%) | -29.6% (-40%, -18.2%) |
| **Kenya** | 1355 (879.9, 1978) | 20.8 (12.8, 31.9) | 4861.6 (3190.1, 6854.1) | 27.3 (16.6, 40.5) | 1.2% (0.9%, 1.4%) | 31.3% (13.7%, 53.6%) | 36136.4 (25098.1, 49387.3) | 430.3 (292.5, 607.9) | 140060 (97070.2, 190517.8) | 585.2 (393.6, 809.3) | 1.3% (1%, 1.6%) | 36% (16.6%, 60%) |
| **Kiribati** | 52.1 (40.3, 65.2) | 132.6 (101.6, 169) | 86.8 (65.3, 113) | 121.7 (88.2, 159.7) | -0.4% (-0.4%, -0.4%) | -8.3% (-27.5%, 17.1%) | 1878.3 (1463.7, 2344.5) | 4071.4 (3169.2, 5069.3) | 3083.6 (2328.4, 3995.6) | 3517.3 (2654.7, 4552.5) | -0.6% (-0.7%, -0.6%) | -13.6% (-33.5%, 13.7%) |
| **Kuwait** | 597.3 (510.9, 695.7) | 98.7 (77.1, 123.7) | 1636.5 (1274.9, 2070) | 59.8 (44.1, 79.4) | -1.5% (-1.9%, -1.1%) | -39.4% (-49.3%, -27.6%) | 19479.6 (17225.4, 22030.9) | 2292 (1934.7, 2684.9) | 52148.8 (42311.5, 64091.2) | 1401.8 (1112.8, 1775.6) | -1.5% (-1.9%, -1.1%) | -38.8% (-49.1%, -26.5%) |
| **Kyrgyzstan** | 3317.8 (2501.9, 4274.2) | 113.7 (84.8, 148.4) | 4994.7 (3599.5, 6697.2) | 130.4 (89.3, 179.7) | 0.6% (0.2%, 1.1%) | 14.7% (-3.2%, 32.1%) | 75893.7 (61572, 91638.4) | 2465.4 (1973.5, 3011.2) | 106713.5 (84644.2, 134877.9) | 2349.5 (1795.8, 3048.2) | -0.2% (-0.7%, 0.2%) | -4.7% (-18%, 8.5%) |
| **Lao People's Democratic Republic** | 1513.5 (1103.5, 2021.9) | 84.6 (59.9, 116.9) | 2791.8 (2027.6, 3774.7) | 74.6 (51.1, 102.1) | -0.6% (-0.7%, -0.5%) | -11.8% (-32.3%, 11.7%) | 44336.8 (32984.8, 58572.1) | 2009.9 (1495.7, 2658.8) | 77194.7 (57194, 100234.6) | 1619.8 (1188.7, 2176.1) | -0.9% (-1%, -0.8%) | -19.4% (-40%, 5.4%) |
| **Latvia** | 6869.8 (5241.2, 8762.8) | 199.5 (151.3, 256.7) | 5029.7 (3425, 7137.8) | 114 (80.2, 158.6) | -2.4% (-2.7%, -2.1%) | -42.9% (-51.1%, -33.2%) | 136505 (113029.2, 163022.1) | 3901.2 (3243.1, 4654) | 79963.8 (58444.1, 106208) | 2062.1 (1553.3, 2669.6) | -2.9% (-3.3%, -2.5%) | -47.1% (-55.3%, -37.2%) |
| **Lebanon** | 3375.5 (2656.9, 4216.7) | 168.3 (125.9, 216.8) | 6120.9 (4108.7, 7913.4) | 120.6 (80.7, 156.4) | -0.9% (-1.1%, -0.6%) | -28.4% (-46.7%, -15.9%) | 87260.2 (70561.1, 106209) | 3727.7 (2991.3, 4545) | 132459.7 (94896.6, 165313.9) | 2537.5 (1828.5, 3159.2) | -1% (-1.3%, -0.7%) | -31.9% (-48%, -17.3%) |
| **Lesotho** | 181.7 (113.8, 280.8) | 22.1 (13.5, 34.8) | 386.3 (236.7, 575.9) | 36.6 (21.8, 56.5) | 2.5% (2.1%, 2.8%) | 65.7% (23.2%, 121.5%) | 4567.8 (2944.6, 6768.5) | 467.5 (298.8, 696.8) | 10863.8 (6753.6, 15707) | 817.8 (505.2, 1197.9) | 2.7% (2.3%, 3.1%) | 74.9% (26.1%, 140.6%) |
| **Liberia** | 431.6 (302.8, 598.1) | 46.9 (32.2, 66.6) | 784.4 (527.5, 1099.2) | 44.5 (28.9, 64.1) | 0% (-0.1%, 0.2%) | -5% (-26.3%, 23.5%) | 10793.2 (7783.9, 14500.6) | 987.8 (713.1, 1339.3) | 21442.6 (14697.5, 29563.9) | 936.8 (641.3, 1299) | 0% (-0.2%, 0.2%) | -5.2% (-28.7%, 26.6%) |
| **Libya** | 1449.8 (1057.9, 1965.1) | 81.3 (56.9, 113.9) | 4172.4 (2987.3, 5685) | 84.4 (58.7, 117.7) | 0.2% (0%, 0.4%) | 3.7% (-18.7%, 33.8%) | 39247.4 (29862.4, 52565.1) | 1878.2 (1414.5, 2511.5) | 115229.1 (86459, 156393.9) | 1947.1 (1444, 2623) | 0.1% (0%, 0.3%) | 3.7% (-19.7%, 36.2%) |
| **Lithuania** | 8982.2 (6889.1, 11253.8) | 204.1 (155.9, 256.7) | 7382.6 (4743.6, 10263.7) | 114.9 (77.4, 157) | -2.3% (-2.5%, -2%) | -43.7% (-52.7%, -33.7%) | 170826.4 (142322, 200203.8) | 3831.9 (3205.8, 4494.6) | 113615.6 (82128.5, 149484.8) | 2019.8 (1518, 2597.9) | -2.5% (-2.8%, -2.2%) | -47.3% (-56.4%, -36.9%) |
| **Luxembourg** | 509.3 (379.7, 666.8) | 97.4 (72.9, 128.1) | 325.9 (216.8, 458.4) | 28.6 (19.9, 39.2) | -4.5% (-4.6%, -4.3%) | -70.6% (-75.1%, -66.2%) | 9461.7 (7719, 11628.7) | 1787.8 (1473.9, 2185.8) | 5062.5 (3828.2, 6507.7) | 496.7 (383.5, 626.3) | -4.7% (-4.8%, -4.6%) | -72.2% (-75.7%, -68.6%) |
| **Madagascar** | 1983.7 (1464.9, 2635.5) | 44.3 (31, 61.6) | 4484.8 (2879.4, 6554.3) | 48.9 (30, 70.5) | 0.2% (0%, 0.4%) | 10.4% (-17.5%, 42.9%) | 57708.3 (44168.8, 73923.7) | 1022.7 (770.9, 1334) | 139850.4 (92801.1, 198223.1) | 1094.2 (720.1, 1568.7) | 0.1% (-0.1%, 0.3%) | 7% (-21.7%, 41.5%) |
| **Malawi** | 1292 (947.8, 1747.9) | 39.6 (28.1, 55.3) | 2468.9 (1712.3, 3395.1) | 39.1 (25.9, 56.4) | -0.2% (-0.4%, 0%) | -1.2% (-22.9%, 25.6%) | 38365 (29347.3, 49450.1) | 921.3 (688.4, 1226.7) | 70270 (50353.5, 92937.4) | 882.4 (628.8, 1189.8) | -0.3% (-0.6%, -0.1%) | -4.2% (-26.6%, 26%) |
| **Malaysia** | 7710.9 (6268.5, 9329.5) | 92.4 (72.4, 115) | 19815.9 (14891.4, 25790.7) | 82.5 (60.4, 108.8) | -0.9% (-1.2%, -0.6%) | -10.7% (-28.3%, 9.3%) | 204081 (173767.1, 238263.5) | 2096.7 (1749.6, 2499.4) | 519367.1 (401796.8, 659958) | 1872.2 (1433.9, 2410.5) | -0.7% (-0.9%, -0.5%) | -10.7% (-28.3%, 10.5%) |
| **Maldives** | 97.9 (80, 119.8) | 127.6 (99.8, 161.4) | 159.5 (117.2, 209) | 57.8 (39.7, 78.8) | -3.4% (-3.7%, -3.2%) | -54.7% (-64%, -45%) | 2928.1 (2442.3, 3555.3) | 2939 (2409.5, 3579.8) | 4082.6 (3289.4, 5020.5) | 1156.4 (891.9, 1473.3) | -3.9% (-4.2%, -3.7%) | -60.6% (-68.3%, -52.4%) |
| **Mali** | 1339.9 (948.4, 1856.9) | 42.1 (28.4, 60.7) | 2825 (1945.8, 3967.7) | 41 (26.8, 59.5) | 0% (-0.1%, 0.1%) | -2.7% (-22.5%, 18.9%) | 34657.9 (25747.8, 46145.2) | 845.1 (612.9, 1158.6) | 70114.4 (49610.9, 95490.4) | 801.2 (567.9, 1111.6) | -0.1% (-0.2%, 0%) | -5.2% (-25.1%, 19.7%) |
| **Malta** | 463.3 (350.7, 583.4) | 117.4 (87.6, 149.2) | 470.8 (323, 631.1) | 47.7 (33.6, 62.8) | -3.1% (-3.2%, -3%) | -59.3% (-64%, -54.8%) | 9053 (7418, 10835.8) | 2165 (1774.1, 2593.5) | 7324.2 (5595, 9247.2) | 840.9 (665, 1032.9) | -3.3% (-3.4%, -3.1%) | -61.2% (-65.2%, -56.8%) |
| **Marshall Islands** | 19.2 (14.4, 24.2) | 124.7 (92.8, 161.4) | 45.5 (32.3, 62.4) | 137.8 (97.9, 188.1) | 0.4% (0.3%, 0.6%) | 10.5% (-11.6%, 38%) | 622.4 (477.2, 772.6) | 3262.5 (2491.3, 4060.3) | 1570.2 (1116.7, 2160.2) | 3639.6 (2613.1, 4931.8) | 0.4% (0.3%, 0.6%) | 11.6% (-13.2%, 42.3%) |
| **Mauritania** | 502.2 (369.9, 686.9) | 58.7 (41.6, 82.9) | 750.2 (518.5, 1036.1) | 42 (28.5, 58.6) | -1.1% (-1.2%, -1%) | -28.4% (-43.2%, -12.1%) | 12875.2 (9752.4, 16889.4) | 1271.3 (968, 1685.5) | 17585.1 (12251.2, 24167.2) | 834 (581, 1146.4) | -1.4% (-1.5%, -1.2%) | -34.4% (-50.1%, -15.2%) |
| **Mauritius** | 993.7 (819.1, 1181) | 147.1 (117, 181.5) | 904.1 (657.5, 1198.9) | 56 (40.3, 74.9) | -4.2% (-4.6%, -3.8%) | -61.9% (-68.8%, -54.4%) | 27578.4 (23814.4, 31735.1) | 3504 (2975.7, 4082.8) | 21312.5 (16568.7, 27219.2) | 1250.2 (967.4, 1591.7) | -4.5% (-4.9%, -4%) | -64.3% (-70.6%, -57.1%) |
| **Mexico** | 19737.4 (15328.4, 24650) | 54.5 (40, 70.8) | 52139.3 (38470.5, 66751.7) | 48 (34.6, 62.8) | -0.4% (-0.7%, -0.2%) | -11.9% (-23.1%, 1.4%) | 471889.9 (396663.5, 555851.2) | 1068 (869.7, 1286.4) | 1140364 (916556.7, 1395811.4) | 960.6 (767.6, 1181.5) | -0.4% (-0.6%, -0.2%) | -10.1% (-21.6%, 4.3%) |
| **Micronesia (Federated States of)** | 58.4 (42, 79.1) | 130.7 (94.3, 178) | 94.5 (62.4, 128.5) | 138.5 (93.3, 189.6) | 0.2% (0.1%, 0.2%) | 5.9% (-23.9%, 41.8%) | 1962.6 (1416.2, 2699.3) | 3650.1 (2636.1, 4933.1) | 3097.1 (1971.9, 4278.4) | 3692.2 (2424.2, 5027) | 0% (0%, 0%) | 1.1% (-34.3%, 40.3%) |
| **Monaco** | 61.9 (41.2, 86.9) | 77.8 (54.1, 106.5) | 45.2 (29.3, 63.9) | 38.9 (26.2, 53.4) | -2.4% (-2.7%, -2.2%) | -50% (-60.3%, -36.2%) | 946.1 (684.3, 1263.3) | 1388.6 (1043, 1812.3) | 625.6 (439.5, 836.3) | 684.9 (500.4, 878.3) | -2.5% (-2.8%, -2.2%) | -50.7% (-62.3%, -35.8%) |
| **Mongolia** | 1563.8 (1203.2, 1954.6) | 174.6 (127.5, 224.7) | 2554 (1877.7, 3393.8) | 140.6 (95.9, 192.2) | -1.4% (-1.8%, -1%) | -19.5% (-35.5%, 0.6%) | 39852 (31668.8, 48761.6) | 3731.1 (2926.7, 4596.5) | 72688.3 (54279.8, 94720) | 2836.7 (2105.8, 3733.9) | -1.7% (-2.1%, -1.3%) | -24% (-41%, -0.9%) |
| **Montenegro** | 493.8 (378.5, 615.8) | 85.7 (64.2, 108.6) | 752 (541.5, 1003) | 83.5 (59.1, 111) | 0.1% (0%, 0.2%) | -2.5% (-18.2%, 14.1%) | 11109 (9216.6, 13024) | 1775.2 (1459.4, 2108.6) | 14933.2 (11337.2, 18981.7) | 1589.6 (1211, 2015.2) | -0.3% (-0.5%, -0.1%) | -10.4% (-26%, 6.8%) |
| **Morocco** | 17201.5 (13199, 21314.8) | 143 (106.2, 183.8) | 36767.4 (25767.5, 47743.1) | 135.3 (93.5, 180.4) | -0.3% (-0.5%, -0.1%) | -5.4% (-23.1%, 10.1%) | 462532.5 (367174.5, 566924.9) | 3213.5 (2516.2, 3936.7) | 900579.2 (645745.4, 1157577.6) | 2806.2 (2010.3, 3595.9) | -0.6% (-0.7%, -0.5%) | -12.7% (-31.6%, 6.3%) |
| **Mozambique** | 1729.4 (1248.7, 2476.7) | 35.4 (24.3, 51.8) | 4415.4 (3053, 6124.6) | 47.4 (31.1, 69) | 1.5% (1.3%, 1.7%) | 34% (2.8%, 74.7%) | 48364.5 (36032.4, 66211.2) | 777.2 (566.6, 1090.9) | 126277.4 (89758.2, 170694.6) | 1062.9 (745.3, 1448.8) | 1.6% (1.4%, 1.9%) | 36.8% (2.9%, 82.7%) |
| **Myanmar** | 15435.8 (11242.4, 20702.4) | 75.4 (53.7, 105.3) | 22312.4 (16121.6, 30557.8) | 56.2 (39.3, 81.1) | -1.2% (-1.3%, -1.1%) | -25.5% (-42.7%, -5.4%) | 455882.9 (334366.2, 615251) | 1811.6 (1324.8, 2439.1) | 549990.8 (423000, 711280.6) | 1165.6 (867.9, 1527.8) | -1.8% (-1.8%, -1.7%) | -35.7% (-52%, -14.8%) |
| **Namibia** | 282 (198.9, 411.9) | 47.8 (32.3, 70.1) | 552.7 (363.7, 807.1) | 45.4 (29.2, 68.1) | -0.4% (-0.7%, -0.1%) | -5.1% (-26.7%, 20.9%) | 7129.2 (5178.5, 9934.2) | 1013.5 (729.8, 1416.8) | 13209.5 (8935.7, 18915.5) | 920.6 (618.8, 1307.7) | -0.6% (-1%, -0.3%) | -9.2% (-32.1%, 19.5%) |
| **Nauru** | 6.9 (5.2, 8.7) | 168 (126.5, 212.6) | 8.2 (6.3, 10.5) | 181.5 (136.2, 233) | 0.2% (-0.1%, 0.5%) | 8% (-10.7%, 30.5%) | 253.1 (192.5, 326.5) | 4608.9 (3497, 5751.5) | 311.9 (239, 400.8) | 4893 (3811.2, 6187.8) | 0.1% (-0.2%, 0.5%) | 6.2% (-13.8%, 31%) |
| **Nepal** | 3743.5 (2714.2, 5187.6) | 41.9 (29.2, 59.9) | 9470 (6510.6, 13067.6) | 47.9 (32.1, 67.4) | 0.5% (0.4%, 0.6%) | 14.1% (-13.9%, 46.1%) | 118179.1 (86657.9, 160570.6) | 1081.5 (791.3, 1495.1) | 250940.5 (179502.2, 336243.3) | 1088.8 (777.8, 1468) | 0.1% (-0.1%, 0.2%) | 0.7% (-24.8%, 32.6%) |
| **Netherlands** | 17367.6 (13196, 22136.9) | 87.3 (66.9, 110.5) | 9912.7 (6852.3, 13811.6) | 27 (19.2, 37) | -4.6% (-4.9%, -4.3%) | -69% (-72.5%, -65.8%) | 332071.2 (273501.7, 398032.6) | 1719.4 (1438.2, 2038.5) | 155484.6 (119127.8, 197457.6) | 478.8 (383.1, 588.2) | -5% (-5.3%, -4.7%) | -72.2% (-74.3%, -69.8%) |
| **New Zealand** | 3828.5 (2936.5, 4794.9) | 101.5 (77.8, 127.5) | 3330.6 (2299.9, 4526.1) | 39.1 (27.9, 52.2) | -3.7% (-3.9%, -3.5%) | -61.5% (-65.7%, -57.5%) | 76942.4 (63746, 91286) | 2032.6 (1698.2, 2391.1) | 51232.5 (39946.8, 64572.6) | 682.6 (550.4, 834.8) | -4.1% (-4.4%, -3.9%) | -66.4% (-69%, -63.8%) |
| **Nicaragua** | 638.1 (489.8, 809.8) | 47 (34.6, 62.3) | 2288.1 (1639.2, 3051.2) | 64.9 (44.6, 89.3) | 0.9% (0.6%, 1.2%) | 38% (15.7%, 58.6%) | 15471.2 (12779.5, 18620.1) | 960.6 (766.1, 1182.4) | 47153.8 (35582.2, 60175.4) | 1108.1 (814.6, 1429.8) | 0.3% (0.1%, 0.5%) | 15.3% (-4.8%, 35.4%) |
| **Niger** | 953.5 (657.8, 1340.6) | 43.3 (28.1, 63.4) | 2510.3 (1648.4, 3597.9) | 41 (26, 60.2) | -0.1% (-0.2%, 0%) | -5.3% (-24.6%, 19.9%) | 27045.7 (19030.5, 37167.4) | 914.1 (634.7, 1278.2) | 68233.8 (45805.2, 96655.6) | 843.6 (560.6, 1196.6) | -0.3% (-0.4%, -0.1%) | -7.7% (-27.3%, 20.3%) |
| **Nigeria** | 18338.6 (11812.7, 27367.9) | 50.5 (31.1, 76.2) | 29795.7 (19680.6, 41280.1) | 42 (26.9, 59.6) | -0.8% (-1%, -0.7%) | -16.9% (-46.5%, 11.2%) | 457717.3 (303373.7, 685364.9) | 1032.2 (676, 1533.7) | 761154.6 (531980.6, 1041290.6) | 831.9 (562.1, 1137.8) | -1% (-1.1%, -0.8%) | -19.4% (-46.5%, 10.3%) |
| **Niue** | 2.2 (1.6, 3) | 98.2 (71.4, 130.5) | 1.9 (1.4, 2.6) | 91.7 (64.8, 121.9) | -0.4% (-0.5%, -0.3%) | -6.6% (-27.3%, 18.8%) | 53.3 (40.2, 68.8) | 2537.7 (1929.4, 3265.8) | 48.5 (35.5, 63) | 2291.2 (1668.7, 3017.4) | -0.6% (-0.7%, -0.4%) | -9.7% (-32.6%, 21.4%) |
| **North Macedonia** | 2367.8 (1807.6, 3157.7) | 149.5 (107.7, 207.7) | 3274.4 (2153.9, 4791.2) | 134 (86, 203.1) | -0.6% (-0.9%, -0.3%) | -10.4% (-26.9%, 6.4%) | 54226.1 (44505.7, 66523.2) | 2955.1 (2352.9, 3737.1) | 64858.1 (45782.2, 89482.6) | 2253.9 (1557.4, 3148.2) | -1.3% (-1.6%, -1.1%) | -23.7% (-38.6%, -6.9%) |
| **Northern Mariana Islands** | 11.8 (9.1, 15.1) | 64.9 (49.1, 85.4) | 34.2 (26.6, 41.9) | 71.4 (53.2, 92.7) | 0.6% (0.5%, 0.6%) | 10.1% (-10.2%, 36%) | 448 (341.6, 572.9) | 1646.9 (1300.9, 2102.4) | 1029.6 (809.1, 1261.6) | 1771.2 (1368.3, 2189.2) | 0.5% (0.4%, 0.6%) | 7.5% (-15.9%, 35.8%) |
| **Norway** | 7195.5 (5325.9, 9342.8) | 101.9 (78.1, 129.4) | 3439.8 (2337.4, 4765.8) | 30.8 (21.7, 42) | -4.5% (-4.7%, -4.4%) | -69.8% (-73.4%, -66.6%) | 127769.6 (102195.8, 156270.9) | 2009.3 (1675.7, 2390.1) | 51321.7 (39125.8, 66029.2) | 538.2 (426.6, 672.4) | -4.8% (-5%, -4.7%) | -73.2% (-75.4%, -70.8%) |
| **Oman** | 1206.8 (879.7, 1565.6) | 215.4 (154.3, 289.1) | 1909.1 (1498.7, 2392.8) | 163.9 (115.7, 216.3) | -0.9% (-1.1%, -0.7%) | -23.9% (-38.2%, -5.6%) | 35075.2 (25978.7, 45928.5) | 4655.6 (3433, 6016.6) | 53954 (43011.6, 65573.6) | 2927.6 (2253.5, 3705.1) | -1.6% (-1.9%, -1.3%) | -37.1% (-50.4%, -19.6%) |
| **Pakistan** | 33228.3 (24158.2, 43605.6) | 61.5 (43.5, 83.1) | 89730.7 (68657.6, 116172.7) | 84.7 (62, 112.8) | 1% (0.7%, 1.3%) | 37.7% (12.5%, 70.9%) | 942641.5 (726100.2, 1174956.9) | 1523.6 (1150.2, 1920) | 2840405.2 (2199463.6, 3636922.9) | 2139.3 (1653.9, 2742.6) | 1% (0.7%, 1.4%) | 40.4% (12.9%, 76.6%) |
| **Palau** | 10.7 (8, 14.6) | 115 (83.7, 157.5) | 22.7 (16.9, 29.2) | 112.6 (81.8, 149.2) | -0.1% (-0.2%, 0%) | -2.1% (-27.1%, 30.7%) | 340.5 (252.5, 455.4) | 3083.1 (2305.4, 4156.9) | 721.2 (540.8, 938.3) | 3025.9 (2269.2, 3917.2) | -0.1% (-0.1%, 0%) | -1.8% (-27.9%, 32.9%) |
| **Palestine** | 1150.4 (831.9, 1535.5) | 146.8 (103.9, 203) | 2107.8 (1616.9, 2737.1) | 108 (77.9, 144.6) | -1.4% (-1.6%, -1.2%) | -26.4% (-41.2%, -6.5%) | 28171.2 (21279.2, 36271.4) | 3178.2 (2375.1, 4138.4) | 54298.1 (43624.3, 66808.3) | 2179.1 (1696.4, 2806.3) | -1.7% (-1.9%, -1.5%) | -31.4% (-46.1%, -11.1%) |
| **Panama** | 722.5 (535, 944.1) | 51.7 (37.4, 69) | 1259.3 (838.7, 1787.6) | 29.1 (19.5, 41) | -1.8% (-2%, -1.5%) | -43.6% (-55%, -30.4%) | 15202.9 (12333.9, 18626.6) | 1001.2 (795.6, 1242) | 24297.6 (17600.2, 32718.7) | 578.3 (418.6, 779.7) | -1.6% (-1.8%, -1.3%) | -42.2% (-54.4%, -27.4%) |
| **Papua New Guinea** | 1090.1 (734, 1596.8) | 60.9 (40.4, 89.3) | 3581 (2478.6, 5070.7) | 75.9 (52.2, 109) | 0.9% (0.8%, 1%) | 24.7% (-2.9%, 64.4%) | 36798.1 (24878.4, 53521.9) | 1648 (1116.4, 2387.2) | 121155.3 (84420.2, 171073.9) | 2001.9 (1407.3, 2810.2) | 0.8% (0.7%, 1%) | 21.5% (-7.4%, 63.4%) |
| **Paraguay** | 1204.2 (918.4, 1558.9) | 58.2 (42.8, 77.5) | 2624.3 (1837.3, 3631.9) | 48.7 (33.8, 68) | -0.6% (-0.8%, -0.3%) | -16.2% (-35.2%, 8.2%) | 27892.7 (22726.3, 33917.8) | 1216 (976.6, 1508.7) | 59856.2 (43810.3, 80052.9) | 1045.7 (761.4, 1401.3) | -0.5% (-0.8%, -0.2%) | -14% (-34.2%, 11.7%) |
| **Peru** | 5060.4 (3758.6, 6637.2) | 45.6 (32.9, 61.1) | 7375.1 (4684.9, 10711) | 22.2 (14.2, 32.3) | -2.4% (-2.8%, -2.1%) | -51.3% (-65.4%, -34.7%) | 119636.4 (94942.5, 148301.4) | 944 (738.8, 1188.2) | 147387.2 (101367.4, 203994.3) | 446 (306.3, 617.7) | -2.5% (-2.8%, -2.1%) | -52.8% (-65.9%, -35.5%) |
| **Philippines** | 10264 (7789.9, 13378.1) | 49.4 (34.3, 66.7) | 51845.6 (39297, 65040.8) | 74.3 (55.1, 95.7) | 2.2% (1.7%, 2.7%) | 50.3% (16.3%, 87.8%) | 278236.8 (225695.1, 367722.8) | 925.2 (710.2, 1191.9) | 1492151.3 (1149223.5, 1841142.4) | 1742.7 (1329, 2156.6) | 3% (2.4%, 3.7%) | 88.4% (30.2%, 138.4%) |
| **Poland** | 67602.2 (52113, 84205.8) | 168.4 (128.2, 213.9) | 46614.8 (31685, 64613.7) | 63.6 (43.9, 87.1) | -3.8% (-3.9%, -3.6%) | -62.2% (-68.1%, -56.2%) | 1462197.2 (1232818, 1712623.1) | 3442.2 (2885.8, 4047.6) | 780674.6 (584287.8, 1011334.3) | 1157 (886.2, 1480.6) | -4.1% (-4.3%, -4%) | -66.4% (-71.7%, -60.7%) |
| **Portugal** | 11314.5 (7842.5, 16508) | 92.5 (62.4, 137) | 8628.4 (5531.3, 12897.5) | 30.3 (20.8, 43.1) | -4.4% (-4.7%, -4.1%) | -67.2% (-70.3%, -63.8%) | 205302 (157655.4, 275230.2) | 1579.1 (1216.6, 2105.8) | 124881.7 (91248, 168350.3) | 534.5 (422.8, 687.7) | -4.3% (-4.6%, -4%) | -66.2% (-68.9%, -63.2%) |
| **Puerto Rico** | 2366.9 (1778.2, 2997.8) | 69.6 (51.9, 89.6) | 2208.7 (1467.7, 3137.5) | 28.3 (19.5, 39.5) | -3.4% (-3.6%, -3.2%) | -59.3% (-67.8%, -49.4%) | 50146.1 (40999.4, 60214.1) | 1417.2 (1163.1, 1693.8) | 39760.2 (28672.8, 53414.3) | 622.5 (458.9, 826.6) | -3.1% (-3.3%, -3%) | -56.1% (-65.4%, -44.9%) |
| **Qatar** | 142.4 (109.9, 180.2) | 167.8 (114.9, 223.4) | 434.6 (307.6, 577.1) | 102.1 (61.4, 148.3) | -2% (-2.3%, -1.8%) | -39.2% (-53.9%, -21.6%) | 4658.5 (3664.8, 5818.8) | 3263.8 (2441.2, 4198.3) | 15187.8 (11195.6, 19805.8) | 1580 (1069.9, 2162.7) | -3% (-3.3%, -2.7%) | -51.6% (-63.9%, -36.6%) |
| **Republic of Korea** | 14452.8 (10972.9, 19058.8) | 64.3 (44.8, 90.9) | 15522.2 (10227, 22896.3) | 19.2 (12.4, 28.7) | -4.4% (-4.6%, -4.2%) | -70.2% (-74.6%, -63.9%) | 389375.9 (311665.9, 485725) | 1264.7 (976.5, 1658.8) | 286052.5 (211208.5, 390697.5) | 336.9 (248.1, 458.9) | -4.7% (-4.9%, -4.5%) | -73.4% (-76.9%, -67.2%) |
| **Republic of Moldova** | 5889.4 (4388.1, 7490) | 170.6 (121.1, 226.9) | 6481.8 (4533.4, 8898.7) | 111.9 (78.3, 154) | -1.8% (-2.1%, -1.5%) | -34.4% (-41.4%, -26.2%) | 120274.2 (96755.6, 146489.1) | 2942.2 (2305.8, 3632.5) | 119524.3 (91996.2, 153650.5) | 2093.7 (1618.5, 2678.5) | -1.4% (-1.8%, -1.1%) | -28.8% (-37%, -19.4%) |
| **Romania** | 38503.7 (29252, 49273.3) | 169.2 (124.3, 222.8) | 39922.1 (26712, 56184.4) | 102.9 (70.9, 143.2) | -2.3% (-2.6%, -2.1%) | -39.2% (-48.5%, -29.1%) | 794512.2 (650941.3, 962268.9) | 3042.7 (2475.5, 3717.5) | 670622 (493040.9, 893577.1) | 1886.9 (1409.3, 2439.6) | -2.4% (-2.7%, -2.1%) | -38% (-47.9%, -26.6%) |
| **Russian Federation** | 299043.3 (233088.9, 384233.8) | 189.8 (144.2, 248.4) | 332216.8 (241611.8, 435259.4) | 142.4 (103.9, 186.7) | -1.6% (-2.1%, -1%) | -24.9% (-33.4%, -15.2%) | 6527143.7 (5460221, 7777373.2) | 3752.5 (3089.4, 4534.9) | 6388323.2 (4897067.2, 7947908.9) | 2805.9 (2172.8, 3474.8) | -1.7% (-2.3%, -1%) | -25.2% (-34.9%, -14%) |
| **Rwanda** | 626.5 (404.8, 947) | 25.6 (15.8, 40.3) | 928.2 (522.7, 1461.9) | 19.5 (10.3, 31.7) | -1.5% (-1.8%, -1.3%) | -23.9% (-48.4%, 0%) | 18296.8 (12052.9, 27336) | 594.4 (388.2, 896.7) | 25106.4 (15042.7, 38285.2) | 397.9 (233.6, 617.9) | -2.2% (-2.5%, -1.9%) | -33.1% (-55.2%, -9.3%) |
| **Saint Kitts and Nevis** | 40.7 (29.9, 54) | 122.9 (90.7, 164.8) | 29.7 (21.4, 40.1) | 54.6 (37.8, 76.9) | -2.8% (-3%, -2.5%) | -55.6% (-61.7%, -47.9%) | 848.6 (665.2, 1072.3) | 2528 (2022.2, 3100.4) | 697.3 (513.6, 905.7) | 1055.2 (770.4, 1395.1) | -3.3% (-3.7%, -3%) | -58.3% (-66.1%, -49.5%) |
| **Saint Lucia** | 49.1 (35, 66.9) | 66.5 (45.7, 92.9) | 61.3 (42, 87.7) | 30.3 (20.6, 44.2) | -3.1% (-3.6%, -2.7%) | -54.4% (-61%, -47.2%) | 1041 (806, 1321.6) | 1252.6 (956.5, 1605.7) | 1279.2 (959.4, 1691.4) | 596 (443.3, 794.7) | -2.8% (-3.2%, -2.4%) | -52.4% (-59.4%, -44.6%) |
| **Saint Vincent and the Grenadines** | 50.2 (36.3, 66.7) | 77.4 (54.4, 103.7) | 64.9 (46.2, 88) | 53.3 (37.2, 73.1) | -1.2% (-1.5%, -0.9%) | -31.1% (-39.5%, -22.5%) | 1074.8 (839.7, 1346.3) | 1544 (1206, 1933) | 1348.5 (1027.8, 1726.1) | 1022.8 (772.9, 1320.7) | -1.4% (-1.7%, -1.2%) | -33.8% (-42.8%, -24%) |
| **Samoa** | 71.8 (52.5, 95.9) | 88.4 (63.3, 120.4) | 122.1 (87.4, 162) | 86.4 (61.1, 115.8) | -0.1% (-0.1%, -0.1%) | -2.3% (-23.9%, 25.4%) | 2073.4 (1537.1, 2706.3) | 2240.4 (1654.9, 2948.9) | 3465.7 (2488.5, 4616) | 2176.9 (1564.4, 2886.7) | -0.1% (-0.1%, -0.1%) | -2.8% (-27.9%, 32.1%) |
| **San Marino** | 14.7 (10.3, 20.4) | 46.7 (32.7, 65.8) | 22 (12.6, 34.3) | 27.5 (16, 42.2) | -1.9% (-2.1%, -1.7%) | -41.1% (-60.4%, -18.6%) | 250.5 (191.4, 329.7) | 788.9 (609.8, 1030.8) | 310 (188.9, 465.6) | 478.1 (301.9, 709.9) | -1.8% (-2%, -1.6%) | -39.4% (-59.5%, -12.6%) |
| **Sao Tome and Principe** | 22.6 (15.6, 30.7) | 43.3 (28.8, 61) | 51.9 (36.6, 68.6) | 59.3 (39.6, 81.4) | 1.1% (1%, 1.3%) | 36.9% (12.1%, 66.6%) | 541.9 (386.8, 711.1) | 874.7 (622.5, 1152.5) | 1297 (936.8, 1683.3) | 1171.4 (844.4, 1517.1) | 1% (0.8%, 1.2%) | 33.9% (6%, 69.4%) |
| **Saudi Arabia** | 5295 (3744, 7034.8) | 99.2 (67.9, 136.9) | 17926 (13553.5, 22954.7) | 103 (73.2, 136.9) | 0.1% (-0.2%, 0.4%) | 3.8% (-19.7%, 38.4%) | 153379.3 (110051, 201160.9) | 2244.3 (1588.1, 2973.4) | 609556.3 (463174.8, 785360.8) | 2406.2 (1815.7, 3037.6) | 0.4% (0%, 0.7%) | 7.2% (-19.6%, 45.7%) |
| **Senegal** | 1266.2 (891.8, 1709.4) | 47 (32.3, 65.5) | 2767.4 (1897.2, 3768) | 43.7 (29.3, 61.5) | -0.2% (-0.3%, -0.1%) | -7% (-24.2%, 15%) | 32819.2 (24098.1, 42779.2) | 993.7 (724.2, 1308.7) | 68520.9 (48646.5, 91253.8) | 889.4 (634.7, 1188.7) | -0.3% (-0.4%, -0.2%) | -10.5% (-30.3%, 14.3%) |
| **Serbia** | 16025.1 (12239, 20898.3) | 165.5 (121.2, 222.6) | 18641.6 (12321.2, 26775) | 134.8 (89, 193.2) | -1.1% (-1.3%, -0.8%) | -18.6% (-33.3%, -3.1%) | 331276.5 (274017.6, 405572.3) | 3063.9 (2465.5, 3844.5) | 315210.2 (224778.6, 433100.4) | 2155.3 (1545.3, 2917.2) | -1.7% (-2%, -1.4%) | -29.7% (-43.8%, -14.7%) |
| **Seychelles** | 45 (35.3, 56.1) | 81.9 (64.1, 102) | 60.7 (47.3, 75.7) | 59.7 (44.8, 76.9) | -1.3% (-1.4%, -1.2%) | -27.1% (-35.1%, -18.4%) | 1070.9 (900.2, 1264) | 1924 (1618.5, 2270.1) | 1550.8 (1284.5, 1850.6) | 1344 (1090, 1619.7) | -1.6% (-1.7%, -1.4%) | -30.1% (-38.4%, -21%) |
| **Sierra Leone** | 823.2 (557, 1168) | 49.2 (32.1, 70.3) | 1553.7 (1033.9, 2178.3) | 50 (32.5, 72) | 0.4% (0.2%, 0.6%) | 1.6% (-18.4%, 30.2%) | 20124.2 (13772.4, 27976.1) | 1041.4 (719.1, 1445.4) | 41585 (28380.9, 57594.6) | 1073.9 (728.3, 1493.4) | 0.4% (0.3%, 0.6%) | 3.1% (-20.8%, 36.5%) |
| **Singapore** | 1682.2 (1367.1, 2039.5) | 86.8 (67.6, 109.4) | 1942.9 (1471.2, 2492.6) | 26.2 (19.3, 34.3) | -4.5% (-4.6%, -4.3%) | -69.8% (-72.9%, -66.8%) | 42486.1 (36264.7, 49380.1) | 1853.7 (1547.7, 2214.9) | 42581.2 (35217.4, 50956.1) | 538.7 (439.4, 649) | -4.6% (-4.7%, -4.4%) | -70.9% (-73%, -68.7%) |
| **Slovakia** | 10681.6 (8203.8, 13314.6) | 190.7 (145.9, 240.5) | 9003 (6116, 12404.3) | 101.6 (68.4, 139.4) | -2.3% (-2.5%, -2.1%) | -46.8% (-57.8%, -35.5%) | 223322.2 (185816.5, 264469.2) | 3830.7 (3203.6, 4540.5) | 154381.2 (111663.1, 205219.1) | 1743.1 (1265.5, 2316.3) | -2.9% (-3.1%, -2.7%) | -54.5% (-64.2%, -44%) |
| **Slovenia** | 2045.8 (1331.4, 2891.6) | 88.8 (57.5, 125.7) | 1673.4 (1054.9, 2519.9) | 33.5 (21.8, 48.9) | -3.9% (-4.1%, -3.7%) | -62.3% (-72.7%, -50.2%) | 41000.2 (28998.8, 55129) | 1702.3 (1205.5, 2290.1) | 26485.4 (18649.2, 36320.7) | 619.9 (451.5, 834.2) | -4.1% (-4.3%, -3.8%) | -63.6% (-73.8%, -50.9%) |
| **Solomon Islands** | 264.2 (194.2, 351.5) | 182.1 (135.8, 245.3) | 665.1 (498.6, 855.9) | 199.7 (149.4, 258.9) | 0.3% (0.3%, 0.4%) | 9.6% (-14.9%, 39.6%) | 9348.8 (6855.1, 12476.7) | 5378 (3997.6, 7134.7) | 23807.8 (17607, 30607) | 5716.5 (4323.1, 7343.3) | 0.2% (0.2%, 0.3%) | 6.3% (-19.9%, 39.7%) |
| **Somalia** | 867.9 (588.6, 1221.8) | 38.3 (25, 55.5) | 2377.9 (1523.3, 3411.2) | 42 (26.2, 63.2) | 0.5% (0.4%, 0.6%) | 9.6% (-20.2%, 46.5%) | 27822.5 (18927.8, 39008.7) | 916.3 (629.9, 1272.1) | 76350.1 (49643.4, 108408.1) | 981.3 (633.1, 1395.9) | 0.4% (0.3%, 0.5%) | 7.1% (-23.4%, 48.4%) |
| **South Africa** | 7295.7 (5471.4, 9555.1) | 37.3 (26.8, 51.2) | 14727.2 (10920.8, 19303) | 38.3 (26.8, 52.5) | 0.1% (-0.3%, 0.6%) | 2.9% (-7.8%, 13.1%) | 207908.2 (170053.6, 257463.1) | 902.3 (715.1, 1131.7) | 358967.6 (285251.8, 445140.5) | 785.9 (609.7, 996.2) | -0.4% (-0.9%, 0.1%) | -12.9% (-21.6%, -3.8%) |
| **South Sudan** | 595.2 (392.1, 855.6) | 30 (19.1, 43.8) | 934.5 (557.2, 1394.8) | 29.3 (16.9, 43.7) | 0% (-0.1%, 0.1%) | -2.1% (-28.4%, 31.4%) | 15792.1 (10742.6, 21960.8) | 646.2 (438.7, 901.7) | 25971.7 (16159.1, 38556.5) | 628.4 (385.5, 915.8) | -0.1% (-0.1%, 0%) | -2.8% (-29.8%, 34.1%) |
| **Spain** | 33970.3 (24657.1, 46457.4) | 66.4 (48, 91.4) | 27347.9 (18347.2, 38665.8) | 23.2 (16.6, 31.4) | -4% (-4.2%, -3.8%) | -65.1% (-68%, -61.6%) | 611312.8 (486599.4, 768662.8) | 1193.1 (962.4, 1484.7) | 409465.1 (312612.3, 525419.5) | 437.1 (354.2, 531.1) | -3.9% (-4.1%, -3.7%) | -63.4% (-65.7%, -60.6%) |
| **Sri Lanka** | 8459.4 (6749.9, 10417.1) | 92.6 (69.8, 121.3) | 12839.4 (8724, 18095.8) | 58.8 (38.7, 83.6) | -1.2% (-1.4%, -1.1%) | -36.5% (-52.9%, -19%) | 226652.8 (191940.8, 266376.8) | 1981.4 (1630.4, 2380.6) | 292561.5 (209431.6, 398136.2) | 1193 (854.6, 1627.9) | -1.6% (-1.8%, -1.5%) | -39.8% (-54.9%, -22.7%) |
| **Sudan** | 12030.6 (8595.3, 16187.5) | 142.2 (97.8, 192.8) | 20202.6 (13663.9, 27614) | 118 (79.8, 161.7) | -0.8% (-0.8%, -0.7%) | -17% (-32.8%, 4%) | 344610.5 (251440, 461127.2) | 3419.1 (2474.5, 4565.9) | 555250.3 (372269.3, 779424.4) | 2633.1 (1788.5, 3623.9) | -1% (-1.1%, -0.9%) | -23% (-40.4%, 2.2%) |
| **Suriname** | 221.2 (173.8, 275.3) | 87.7 (67.5, 111.8) | 339.3 (254.8, 441.1) | 58.4 (43.2, 76.9) | -1.5% (-1.9%, -1.1%) | -33.4% (-44%, -21.2%) | 5413.3 (4479.3, 6399.9) | 1983 (1620.4, 2382.1) | 8392.4 (6642.3, 10575.3) | 1351 (1058, 1702.9) | -1.5% (-1.9%, -1.1%) | -31.9% (-43.4%, -19%) |
| **Sweden** | 16943.6 (12271.9, 22270) | 105.8 (78.8, 136.6) | 9414.2 (6208.3, 13181.3) | 37.2 (25.8, 50.5) | -3.8% (-4%, -3.7%) | -64.9% (-68.4%, -61%) | 276335 (215726.8, 339494.3) | 1895.5 (1544.5, 2262.7) | 131431 (97478.2, 169876.6) | 631.3 (494.4, 783.9) | -4% (-4.2%, -3.8%) | -66.7% (-69.2%, -64%) |
| **Switzerland** | 8806 (6497.2, 11552.5) | 80.4 (60.1, 103.7) | 6418.8 (4104.9, 9077) | 29.2 (19.9, 39.9) | -3.9% (-4.1%, -3.7%) | -63.7% (-67.9%, -59%) | 146814.8 (118190.4, 179565.7) | 1432.9 (1178.5, 1723.8) | 83749.4 (61769.3, 108014) | 461.9 (361.3, 575) | -4.3% (-4.4%, -4.1%) | -67.8% (-70.5%, -64.7%) |
| **Syrian Arab Republic** | 9032.5 (6882.1, 11669) | 183.1 (135.6, 241.8) | 16712.3 (11696.2, 22871.1) | 167.5 (112.5, 231.3) | -0.7% (-0.9%, -0.5%) | -8.5% (-30.6%, 22.1%) | 251626.9 (196033.4, 314831.7) | 4243.1 (3268.5, 5375.9) | 429420 (311636.2, 584482.3) | 3423.1 (2447, 4651.2) | -1.1% (-1.3%, -0.9%) | -19.3% (-40.5%, 11.3%) |
| **Taiwan (Province of China)** | 5809 (4492.8, 7550) | 48.1 (35.1, 65.2) | 9262.4 (6256, 12959.9) | 23 (15.6, 32.1) | -2.6% (-2.8%, -2.5%) | -52.2% (-61.5%, -40.6%) | 144203 (117822.1, 176664.5) | 949.4 (758.8, 1195) | 198783.2 (148417.5, 262845.1) | 520.4 (391.3, 682.3) | -2% (-2.1%, -1.9%) | -45.2% (-54.9%, -32.2%) |
| **Tajikistan** | 2656.6 (2007.8, 3377.4) | 96 (71.2, 122.7) | 5675.2 (4148.3, 7496.2) | 163.7 (111.5, 226.3) | 2% (1.7%, 2.2%) | 70.4% (38.9%, 105.3%) | 62216.6 (50203.4, 75028.7) | 2145.9 (1713, 2619.9) | 144575.6 (109698.8, 187286.5) | 2981.6 (2170.2, 3916.5) | 1.1% (0.9%, 1.3%) | 38.9% (13.1%, 70.4%) |
| **Thailand** | 14834 (11706.1, 18604.8) | 49.7 (37, 65) | 29280.2 (19885.4, 41037.5) | 29.6 (20, 41.6) | -2.4% (-2.6%, -2.2%) | -40.3% (-55.1%, -23%) | 406922.9 (339369.6, 492120.5) | 1069.3 (866.9, 1316.3) | 688422.2 (495882.2, 927659.3) | 691 (496.7, 931.5) | -2.1% (-2.4%, -1.9%) | -35.4% (-51.4%, -16.6%) |
| **Timor-Leste** | 106.1 (75.1, 146.9) | 45.5 (30.5, 64) | 417.8 (276.1, 579.2) | 63.6 (41.2, 89.3) | 1.3% (1.1%, 1.4%) | 39.6% (7.3%, 75%) | 3211 (2326.5, 4372.4) | 988.1 (704.4, 1351.8) | 10462.4 (7019.7, 14394.4) | 1317.3 (894.6, 1800.2) | 1.1% (0.9%, 1.3%) | 33.3% (-4%, 73.7%) |
| **Togo** | 552.2 (399.9, 736.7) | 54.1 (37.5, 74.7) | 1653 (1139.8, 2277.2) | 54.8 (36.2, 77) | 0.1% (0%, 0.2%) | 1.2% (-16.5%, 26.6%) | 15275.8 (11420.8, 20164.2) | 1143.9 (845.5, 1521.3) | 47408.2 (33250.6, 64819.6) | 1178.4 (826, 1612.6) | 0.2% (0%, 0.3%) | 3% (-18.9%, 34.4%) |
| **Tokelau** | 1.1 (0.8, 1.5) | 87.8 (63, 120.8) | 1.1 (0.8, 1.5) | 88.5 (62.4, 120.6) | 0% (-0.1%, 0.2%) | 0.7% (-23.9%, 33.2%) | 27.8 (20.2, 37.3) | 2196.6 (1608.3, 2910.6) | 28.5 (20.8, 38.5) | 2165.8 (1577, 2941.3) | 0% (-0.2%, 0%) | -1.4% (-28.4%, 36.7%) |
| **Tonga** | 32.2 (24.6, 40.8) | 63.1 (46.9, 81.6) | 51.2 (37, 67) | 65.1 (47, 85.4) | 0.1% (0%, 0.3%) | 3.3% (-18.8%, 31.2%) | 929.2 (736.4, 1138.9) | 1588.3 (1246.5, 1958.7) | 1321.1 (991.7, 1704) | 1616.2 (1211.2, 2081.4) | 0% (-0.1%, 0.2%) | 1.8% (-21.7%, 31.5%) |
| **Trinidad and Tobago** | 856.9 (675.4, 1051.1) | 113.8 (87.2, 143.8) | 1098.9 (755.2, 1527.4) | 61.7 (42, 86.2) | -2.8% (-3%, -2.5%) | -45.8% (-58.2%, -30.6%) | 21439.4 (18084.9, 25313.6) | 2527.4 (2108.8, 3006.4) | 24932.6 (17991.1, 33958.9) | 1366.1 (982.2, 1854.4) | -2.8% (-3.1%, -2.5%) | -46% (-59.1%, -29.5%) |
| **Tunisia** | 4484.8 (3374.5, 5782.5) | 109.8 (78.7, 145.8) | 10679.8 (7076.8, 14968.2) | 94.3 (61.5, 134.5) | -0.7% (-0.8%, -0.6%) | -14.2% (-35.2%, 11.5%) | 111785.4 (88747.8, 138749.1) | 2225.1 (1719.3, 2815.8) | 231580.3 (159846.9, 317839.4) | 1847 (1270.4, 2541.3) | -0.9% (-1%, -0.8%) | -17% (-38.5%, 9.9%) |
| **Turkey** | 37333.5 (30168.8, 46644.5) | 109.5 (84.4, 140.2) | 44344.4 (30408.1, 62705.1) | 53.3 (35.6, 76.3) | -2.5% (-2.7%, -2.3%) | -51.3% (-62.4%, -38.2%) | 997904.7 (819685.2, 1202192.1) | 2572.6 (2097.5, 3106.1) | 942752.1 (703743.1, 1248490) | 1060.8 (782.2, 1418.2) | -3.4% (-3.6%, -3.2%) | -58.8% (-68.6%, -47.4%) |
| **Turkmenistan** | 2866.9 (2277.6, 3531.5) | 174.6 (128.5, 225.3) | 5501.2 (3989.6, 7209.4) | 159.9 (111.7, 217.8) | -1.2% (-1.6%, -0.7%) | -8.4% (-24.4%, 10.6%) | 70667.3 (58973.8, 82926.9) | 3629.4 (2965.1, 4339.5) | 136809 (102427.7, 174870) | 3343.7 (2463.8, 4337.1) | -1.2% (-1.6%, -0.7%) | -7.9% (-24.9%, 14.2%) |
| **Tuvalu** | 7.1 (5.3, 9.4) | 113.8 (84.5, 151.7) | 11.2 (8.1, 14.9) | 119.1 (84.8, 161.6) | 0.2% (0.1%, 0.2%) | 4.7% (-19.9%, 40.8%) | 219 (165.3, 285.3) | 3016.7 (2283.6, 3948.7) | 326 (236.7, 439.3) | 3093.3 (2255.6, 4138.7) | 0.2% (0.1%, 0.2%) | 2.5% (-23.6%, 42.4%) |
| **Uganda** | 1171.1 (715.3, 1730.1) | 22.9 (13.4, 35.4) | 3090 (1906.3, 4477.8) | 26.7 (15.5, 40.2) | 0.3% (0%, 0.6%) | 16.5% (-7.3%, 46.5%) | 30897 (20013.6, 44660.5) | 473.1 (299.8, 684.4) | 84621.1 (54540.4, 119666.1) | 555.4 (352.7, 796.9) | 0.2% (-0.1%, 0.6%) | 17.4% (-7.6%, 51.8%) |
| **Ukraine** | 126427 (95383.1, 161572.3) | 197.9 (146.5, 256.8) | 164333.4 (120381.5, 217141.9) | 216.5 (159.8, 284.3) | -0.4% (-0.8%, 0%) | 9.4% (-5.7%, 27%) | 2457224.2 (1998101.8, 2955668) | 3575.8 (2889.6, 4314.9) | 3096269.6 (2416819.9, 3890929.7) | 4222.5 (3338.3, 5252.2) | -0.2% (-0.6%, 0.3%) | 18.1% (0%, 39.3%) |
| **United Arab Emirates** | 550.9 (413.4, 737) | 156.4 (111.8, 214.7) | 3909.6 (2717.8, 5540.8) | 103.1 (71.4, 143.2) | -1.7% (-2.2%, -1.2%) | -34.1% (-48.3%, -16.1%) | 19967.3 (15111.7, 26620.8) | 3227.6 (2426.8, 4261.6) | 155912.8 (110914.4, 219602.4) | 2250 (1645.7, 3008.7) | -1.5% (-1.9%, -1.2%) | -30.3% (-47.5%, -8.3%) |
| **United Kingdom** | 102966.4 (77764.3, 131257) | 114.3 (87.9, 143.9) | 48027.8 (33827.2, 65551.9) | 35 (25.8, 46.3) | -4.6% (-4.9%, -4.2%) | -69.3% (-71.7%, -67.1%) | 1905139.2 (1559263.9, 2294119.1) | 2232.7 (1875.4, 2632.8) | 777929.3 (609834, 964606.2) | 662.8 (542.7, 799.6) | -4.6% (-4.9%, -4.3%) | -70.3% (-71.8%, -68.6%) |
| **United Republic of Tanzania** | 2654.8 (1857.3, 3717.7) | 31.2 (20.6, 45.4) | 8651.3 (5623, 12187.7) | 42.4 (26.8, 61.4) | 1.1% (1%, 1.2%) | 35.8% (6.2%, 72.8%) | 71042.5 (52276.8, 95325.1) | 647.6 (466.7, 890) | 219149.4 (147734.2, 298991.7) | 859.7 (572, 1184.1) | 1% (0.8%, 1.1%) | 32.7% (2.5%, 74.5%) |
| **United States of America** | 353088.3 (267439.8, 443654.8) | 108.2 (83.4, 134.4) | 226342.6 (158853.5, 304367) | 38 (27.7, 50) | -3.8% (-4.1%, -3.5%) | -64.9% (-68%, -61.8%) | 6588947.9 (5442300.5, 7741075.5) | 2141.6 (1811.3, 2476.6) | 4167271.9 (3307627, 5112982.8) | 805 (656.3, 968.3) | -3.5% (-3.7%, -3.2%) | -62.4% (-65.1%, -59.9%) |
| **United States Virgin Islands** | 64 (48.7, 81.8) | 89.2 (63.9, 117.1) | 127.7 (94.5, 164.6) | 77.9 (56.9, 100.6) | -0.2% (-0.4%, -0.1%) | -12.6% (-24.8%, 2.4%) | 1594.6 (1272.8, 1967.5) | 1813.1 (1423, 2258.6) | 2651.9 (2069.4, 3289.8) | 1560.8 (1222.4, 1957.9) | -0.3% (-0.5%, -0.1%) | -13.9% (-28.3%, 4.3%) |
| **Uruguay** | 3178.2 (2392.5, 4150.6) | 84.9 (63.6, 111) | 2381.7 (1685.2, 3284.1) | 39.4 (29.4, 52) | -2.7% (-2.8%, -2.6%) | -53.6% (-56.6%, -50.5%) | 62534.1 (50728.8, 76394.4) | 1676.5 (1374.9, 2024.6) | 40539.7 (32163.3, 50892.8) | 775.1 (636, 944) | -2.7% (-2.8%, -2.6%) | -53.8% (-56.5%, -51%) |
| **Uzbekistan** | 15124.3 (11451.1, 19153) | 146.1 (107.3, 188.8) | 37740.9 (29294.3, 47218.5) | 306.9 (209.6, 412.8) | 2.8% (2.1%, 3.4%) | 110.1% (82.5%, 137.4%) | 337306 (276956.8, 399924.2) | 2934.9 (2355.5, 3536.4) | 1013925.2 (801840.6, 1238845.1) | 5339.3 (4011.1, 6779.5) | 2.1% (1.6%, 2.8%) | 81.9% (55.2%, 109.2%) |
| **Vanuatu** | 77.2 (55, 106.4) | 124 (88.1, 170.1) | 243.9 (177.9, 329) | 150.1 (106.8, 205.7) | 0.5% (0.4%, 0.6%) | 21% (-6.6%, 61%) | 2514.3 (1767.1, 3431.5) | 3251.2 (2321.3, 4430.7) | 7706.7 (5555.9, 10450.8) | 3945.8 (2894, 5264.8) | 0.4% (0.3%, 0.6%) | 21.4% (-9.9%, 67.5%) |
| **Venezuela (Bolivarian Republic of)** | 6379.5 (4958.8, 7881.7) | 70.5 (52.9, 89.9) | 15271.7 (10547.8, 21296.8) | 53.9 (37, 75.9) | -1.2% (-1.4%, -1%) | -23.5% (-40.1%, -3.3%) | 166286.3 (138488.8, 196280.3) | 1576.6 (1284.4, 1898) | 354243.9 (253644.6, 480857.6) | 1189 (847.6, 1621.5) | -1.3% (-1.4%, -1.1%) | -24.6% (-42.1%, -2.3%) |
| **Viet Nam** | 18979.1 (13357.5, 26531) | 53.3 (36.3, 76.8) | 40602.9 (27565.2, 57220.4) | 50.2 (32.5, 72.8) | -0.2% (-0.2%, -0.1%) | -5.8% (-27.3%, 16.2%) | 445146.5 (328820.2, 596535.8) | 1111.5 (810.6, 1513) | 929326.3 (681811.4, 1242922.8) | 985.4 (708.2, 1333.7) | -0.4% (-0.5%, -0.3%) | -11.3% (-33.6%, 13.8%) |
| **Yemen** | 7124 (4964.1, 9427.7) | 161.2 (111.9, 215.6) | 16920.5 (12108.3, 23168.9) | 142.7 (100, 196.3) | -0.5% (-0.6%, -0.5%) | -11.5% (-29.1%, 15.3%) | 212964.1 (149350.3, 286385.4) | 3857.7 (2723.2, 5118.7) | 485659.2 (344352.3, 659930.9) | 3219.6 (2322.1, 4394.9) | -0.8% (-0.9%, -0.7%) | -16.5% (-36%, 13%) |
| **Zambia** | 935.1 (684.1, 1303.1) | 37.1 (25.7, 53.3) | 2112.3 (1399.8, 2999.7) | 36.9 (23, 53.8) | -0.3% (-0.6%, 0%) | -0.5% (-27.6%, 31.5%) | 27980.3 (21103.8, 37514.3) | 874.2 (642.9, 1200.7) | 62806.5 (43837.6, 86912.3) | 817.4 (554.2, 1140.1) | -0.6% (-1%, -0.3%) | -6.5% (-32.4%, 26.7%) |
| **Zimbabwe** | 1550.4 (1135, 2059.9) | 48.7 (33.7, 67.9) | 3571.3 (2485.6, 4868.5) | 62.4 (41.8, 89.8) | 1.3% (1%, 1.5%) | 28.1% (2.7%, 60.3%) | 39235.3 (29896.8, 50267.6) | 981.3 (733.3, 1283.4) | 95795.3 (68196, 130347.8) | 1332.7 (942, 1806.8) | 1.5% (1.2%, 1.8%) | 35.8% (6.6%, 71.5%) |

Abbreviations: DALY, disability adjusted life year; ASR, age-standardized rate; UI, uncertainty interval; EAPC, estimated annual percentage change.

# Supplementary Table 5 SEV attributable to high LDL-C of all countries and territories

| **Country** | **1990 age-standardized SEV rate per 100,000 people No. (95%UI)** | **2019 age-standardized SEV rate per 100,000 people No. (95%UI)** | **1990-2019 EAPC %. (95%CI)** | **percentage change in age-standardized DALY rates, 1990-2019 (95%UI)** |
| --- | --- | --- | --- | --- |
| **Afghanistan** | 31 (27.8, 34.4) | 32.9 (29.6, 36.5) | 0.2% (0.2%, 0.3%) | 6.1% (1.6%, 10.6%) |
| **Albania** | 43.9 (40.8, 47.2) | 40.5 (37.3, 43.7) | -0.3% (-0.3%, -0.2%) | -7.8% (-11.7%, -3.6%) |
| **Algeria** | 28.6 (25.3, 32.1) | 31.1 (27.8, 34.5) | 0.3% (0.3%, 0.3%) | 9% (4.6%, 13.9%) |
| **American Samoa** | 29.9 (26.7, 33.4) | 31.6 (28.4, 34.9) | 0.1% (0%, 0.2%) | 5.6% (1.6%, 10.3%) |
| **Andorra** | 52.2 (49.4, 55.2) | 50 (47, 53) | -0.2% (-0.3%, -0.2%) | -4.3% (-8.3%, 0%) |
| **Angola** | 20.7 (17.5, 24.1) | 21.8 (18.7, 25.2) | 0.1% (0.1%, 0.2%) | 5.3% (1%, 10%) |
| **Antigua and Barbuda** | 30.4 (27.2, 33.9) | 32 (28.8, 35.4) | 0.2% (0.2%, 0.2%) | 5.2% (1%, 10.1%) |
| **Argentina** | 37.3 (34, 40.8) | 41.5 (38.3, 44.7) | 0.4% (0.4%, 0.4%) | 11.3% (6.7%, 16.1%) |
| **Armenia** | 23.5 (20.4, 26.9) | 25 (21.8, 28.4) | 0.2% (0.2%, 0.2%) | 6.3% (2.2%, 10.6%) |
| **Australia** | 49.4 (46.5, 52.3) | 48.6 (45.5, 51.7) | 0% (-0.1%, 0.1%) | -1.5% (-5.5%, 2.4%) |
| **Austria** | 55.8 (52.8, 58.9) | 54.3 (51.5, 57.4) | -0.1% (-0.2%, -0.1%) | -2.7% (-6.9%, 1.7%) |
| **Azerbaijan** | 32.3 (29, 35.7) | 31 (27.9, 34.4) | -0.2% (-0.2%, -0.1%) | -4.1% (-8%, -0.1%) |
| **Bahamas** | 31.1 (27.8, 34.5) | 33.3 (30, 36.8) | 0.3% (0.3%, 0.3%) | 7.1% (2.9%, 11.7%) |
| **Bahrain** | 47 (43.9, 50.2) | 44.1 (40.7, 47.3) | -0.4% (-0.4%, -0.3%) | -6.2% (-10.4%, -1.5%) |
| **Bangladesh** | 16.9 (13.8, 20.1) | 17.5 (14.4, 20.7) | 0% (0%, 0.1%) | 3.7% (-0.7%, 8.4%) |
| **Barbados** | 33.9 (30.7, 37.2) | 35.6 (32.4, 38.9) | 0.2% (0.1%, 0.2%) | 5.1% (1%, 9.6%) |
| **Belarus** | 37.4 (34.2, 40.7) | 35 (31.8, 38.3) | -0.3% (-0.3%, -0.2%) | -6.5% (-10.3%, -2.7%) |
| **Belgium** | 56.7 (53.8, 59.5) | 40.5 (37.4, 43.7) | -1.5% (-1.7%, -1.3%) | -28.5% (-32.2%, -24.9%) |
| **Belize** | 28.3 (25.1, 31.6) | 32 (28.7, 35.5) | 0.4% (0.3%, 0.5%) | 13% (8.6%, 17.8%) |
| **Benin** | 20 (16.9, 23.4) | 22.2 (19.1, 25.6) | 0.4% (0.4%, 0.4%) | 11.1% (6.5%, 16.1%) |
| **Bermuda** | 35.4 (32, 38.7) | 36.6 (33.4, 39.9) | 0.1% (0.1%, 0.1%) | 3.5% (-0.7%, 7.7%) |
| **Bhutan** | 33.4 (30.1, 36.8) | 39 (35.8, 42.4) | 0.6% (0.6%, 0.6%) | 16.7% (11.9%, 22.6%) |
| **Bolivia (Plurinational State of)** | 28.5 (25.4, 31.7) | 31.3 (28.2, 34.6) | 0.4% (0.3%, 0.4%) | 9.8% (5.6%, 14.6%) |
| **Bosnia and Herzegovina** | 44.1 (40.8, 47.2) | 43.1 (39.9, 46.3) | -0.1% (-0.1%, 0%) | -2.1% (-6.3%, 2.3%) |
| **Botswana** | 16.4 (13.4, 19.6) | 18.3 (15.2, 21.5) | 0.4% (0.4%, 0.4%) | 11.7% (6.7%, 17.1%) |
| **Brazil** | 40.3 (37, 43.7) | 45.1 (41.9, 48.3) | 0.4% (0.4%, 0.4%) | 11.8% (6.9%, 17.2%) |
| **Brunei Darussalam** | 38.8 (35.6, 42) | 42.5 (39.3, 45.9) | 0.4% (0.3%, 0.4%) | 9.7% (5%, 14.7%) |
| **Bulgaria** | 42.3 (39.2, 45.7) | 43.4 (40.1, 46.6) | 0% (-0.1%, 0%) | 2.4% (-1.9%, 6.9%) |
| **Burkina Faso** | 9.5 (7.1, 12.1) | 9.6 (7.2, 12.2) | 0% (0%, 0%) | 1.2% (-5.1%, 7.6%) |
| **Burundi** | 18.1 (15, 21.3) | 18.3 (15.3, 21.6) | -0.1% (-0.1%, 0%) | 1.4% (-2.8%, 5.9%) |
| **Cabo Verde** | 22.6 (19.4, 26) | 24.4 (21.3, 27.7) | 0.6% (0.5%, 0.6%) | 8.1% (4.1%, 12.8%) |
| **Cambodia** | 28.3 (25, 31.7) | 28.9 (25.8, 32.2) | 0.3% (0.2%, 0.3%) | 2.2% (-1.7%, 6.6%) |
| **Cameroon** | 7.4 (5.2, 9.7) | 8.9 (6.5, 11.4) | 0.1% (0%, 0.1%) | 20.2% (12.9%, 29.6%) |
| **Canada** | 44.3 (41.2, 47.4) | 37.9 (34.6, 41.2) | 0.7% (0.6%, 0.7%) | -14.5% (-18.1%, -10.6%) |
| **Central African Republic** | 20.1 (17, 23.4) | 20.5 (17.4, 23.8) | -0.8% (-1%, -0.7%) | 1.8% (-2.3%, 5.7%) |
| **Chad** | 18.8 (15.7, 22.1) | 21.4 (18.2, 24.7) | 0.1% (0.1%, 0.1%) | 13.8% (8.5%, 19.6%) |
| **Chile** | 35.3 (32, 38.7) | 39.7 (36.6, 43.2) | 0.5% (0.5%, 0.6%) | 12.5% (8%, 17.8%) |
| **China** | 28.8 (25.7, 32.2) | 31.9 (28.8, 35.2) | 0.4% (0.4%, 0.4%) | 10.7% (9.1%, 12.7%) |
| **Colombia** | 35.2 (31.9, 38.5) | 39.4 (36, 42.9) | 0.4% (0.4%, 0.4%) | 12% (6.9%, 17.3%) |
| **Comoros** | 25.9 (22.7, 29.3) | 28.6 (25.4, 32.1) | 0.4% (0.4%, 0.4%) | 10.6% (6.1%, 15.7%) |
| **Congo** | 22.6 (19.5, 26.1) | 24.2 (21.1, 27.7) | 0.3% (0.3%, 0.3%) | 6.8% (2.5%, 11.3%) |
| **Cook Islands** | 32.6 (29.5, 36.1) | 37.2 (34, 40.4) | 0.2% (0.2%, 0.2%) | 14% (9.3%, 19%) |
| **Costa Rica** | 34.4 (31.2, 37.7) | 41.3 (38.2, 44.6) | 0.4% (0.3%, 0.4%) | 20.1% (15.1%, 25.9%) |
| **Croatia** | 44.6 (41.5, 47.9) | 43.9 (40.7, 47.1) | 0.6% (0.6%, 0.7%) | -1.5% (-5.4%, 2.9%) |
| **Cuba** | 30.7 (27.5, 34.1) | 31 (27.7, 34.4) | 0% (-0.1%, 0%) | 1% (-3.1%, 5.2%) |
| **Cyprus** | 48.5 (45.3, 51.8) | 49.5 (46.5, 52.7) | 0% (-0.1%, 0%) | 2.1% (-2.5%, 6.8%) |
| **Czechia** | 59.3 (56.5, 62.3) | 44.4 (41.1, 47.8) | 0.1% (0.1%, 0.1%) | -25.2% (-29.4%, -21.5%) |
| **C么te d'Ivoire** | 19.6 (16.5, 22.9) | 22.8 (19.5, 26.2) | -1.2% (-1.4%, -1.1%) | 16.4% (11.3%, 22.1%) |
| **Democratic People's Republic of Korea** | 35 (31.9, 38.6) | 36.8 (33.5, 40.3) | 0.1% (0.1%, 0.2%) | 5.2% (1%, 9.4%) |
| **Democratic Republic of the Congo** | 21.4 (18.2, 24.7) | 21.6 (18.5, 25.1) | 0% (-0.1%, 0%) | 1.4% (-2.6%, 5.5%) |
| **Denmark** | 57.9 (55.2, 60.6) | 46.4 (43.4, 49.6) | -1.1% (-1.3%, -1%) | -19.8% (-23.5%, -16%) |
| **Djibouti** | 17.9 (14.9, 21.3) | 19.5 (16.5, 22.8) | 0.2% (0.1%, 0.3%) | 8.9% (4.8%, 14%) |
| **Dominica** | 24.7 (21.5, 28.2) | 27.6 (24.3, 30.9) | 0.4% (0.4%, 0.4%) | 11.6% (7.1%, 16.8%) |
| **Dominican Republic** | 32 (28.8, 35.3) | 35.3 (32, 38.7) | 0.4% (0.4%, 0.5%) | 10.4% (5.7%, 15.6%) |
| **Ecuador** | 32.4 (29.2, 35.8) | 34.7 (31.6, 38.2) | 0.2% (0.2%, 0.2%) | 7.1% (2.7%, 11.7%) |
| **Egypt** | 30.1 (26.9, 33.5) | 33.6 (30.4, 37) | 0.3% (0.2%, 0.3%) | 11.8% (7.2%, 17%) |
| **El Salvador** | 36.6 (33.4, 39.9) | 40.9 (37.7, 44.1) | 0.4% (0.4%, 0.5%) | 11.8% (7.3%, 17.4%) |
| **Equatorial Guinea** | 20.5 (17.4, 23.9) | 23.7 (20.5, 27) | 0.5% (0.5%, 0.5%) | 15.6% (10.5%, 21.2%) |
| **Eritrea** | 18.2 (15.1, 21.5) | 19.5 (16.4, 22.7) | 0.1% (0%, 0.2%) | 7.2% (2.9%, 12.2%) |
| **Estonia** | 51.6 (48.7, 54.6) | 53.3 (50.4, 56.5) | 0.1% (0.1%, 0.1%) | 3.4% (-0.9%, 8%) |
| **Eswatini** | 13.8 (11, 16.8) | 17.1 (14.2, 20.4) | 0.7% (0.7%, 0.8%) | 23.6% (17.6%, 31.6%) |
| **Ethiopia** | 13.4 (10.5, 16.3) | 14.4 (11.5, 17.4) | 0.3% (0.2%, 0.3%) | 7.6% (2.9%, 13.3%) |
| **Fiji** | 34.7 (31.5, 38.1) | 38.2 (35, 41.5) | 0.3% (0.2%, 0.3%) | 10.3% (5.5%, 15.3%) |
| **Finland** | 55.9 (53.3, 58.8) | 41.7 (38.6, 45) | -1.2% (-1.3%, -1.1%) | -25.4% (-29%, -22%) |
| **France** | 55.8 (52.9, 58.9) | 48.9 (46, 52) | -0.6% (-0.7%, -0.5%) | -12.3% (-15.7%, -8.7%) |
| **Gabon** | 21.8 (18.7, 25.1) | 25.2 (22.1, 28.6) | 0.5% (0.4%, 0.5%) | 15.7% (10.7%, 21.8%) |
| **Gambia** | 23.7 (20.6, 26.9) | 26.2 (23, 29.5) | 0.4% (0.3%, 0.4%) | 10.6% (6.3%, 15.7%) |
| **Georgia** | 26.3 (23.1, 29.6) | 28 (24.9, 31.5) | 0.2% (0.2%, 0.2%) | 6.4% (2.1%, 11%) |
| **Germany** | 59.2 (56.8, 61.6) | 47.9 (44.7, 51.1) | -1% (-1.2%, -0.8%) | -19.1% (-22.4%, -15.6%) |
| **Ghana** | 28.7 (25.6, 32.1) | 33.5 (30.4, 36.9) | 0.6% (0.5%, 0.6%) | 16.8% (12.1%, 22.1%) |
| **Greece** | 40.7 (37.6, 44) | 41 (37.7, 44.2) | 0% (-0.1%, 0%) | 0.7% (-3.7%, 5.2%) |
| **Greenland** | 39.6 (36.3, 42.9) | 35.5 (32.1, 38.8) | -0.4% (-0.5%, -0.4%) | -10.4% (-14.4%, -6.4%) |
| **Grenada** | 28.7 (25.4, 32.2) | 31.4 (28.1, 34.6) | 0.3% (0.2%, 0.3%) | 9.4% (4.8%, 14.7%) |
| **Guam** | 33.4 (30.2, 36.7) | 37 (33.8, 40.2) | 0.4% (0.4%, 0.4%) | 10.7% (6.3%, 15.9%) |
| **Guatemala** | 24.4 (21.2, 27.7) | 29.5 (26.2, 32.9) | 0.8% (0.7%, 0.8%) | 20.8% (15.5%, 26.7%) |
| **Guinea** | 20.9 (17.8, 24.4) | 23.6 (20.5, 26.9) | 0.4% (0.4%, 0.4%) | 12.5% (8%, 18.5%) |
| **Guinea-Bissau** | 19.4 (16.3, 22.6) | 22.9 (19.7, 26.3) | 0.6% (0.6%, 0.7%) | 17.9% (13%, 24.2%) |
| **Guyana** | 29.8 (26.5, 33.3) | 31.3 (28, 34.8) | 0.2% (0.1%, 0.2%) | 4.8% (0.6%, 9.4%) |
| **Haiti** | 26.7 (23.6, 30.1) | 28.2 (24.9, 31.5) | 0.2% (0.2%, 0.2%) | 5.5% (1.4%, 9.7%) |
| **Honduras** | 30.1 (26.9, 33.6) | 34.3 (31, 37.7) | 0.5% (0.5%, 0.6%) | 13.9% (9.4%, 19.3%) |
| **Hungary** | 56.8 (54, 59.7) | 42.5 (39.4, 45.8) | -1% (-1.1%, -0.9%) | -25.1% (-28.9%, -21.6%) |
| **Iceland** | 57.5 (54.8, 60.2) | 56.6 (53.7, 59.8) | -0.1% (-0.1%, 0%) | -1.6% (-5.9%, 2.7%) |
| **India** | 22.3 (19.3, 25.7) | 24 (21, 27.3) | 0.2% (0.2%, 0.3%) | 7.4% (6.1%, 9.1%) |
| **Indonesia** | 21.5 (18.4, 24.8) | 23.6 (20.6, 26.9) | 0.3% (0.3%, 0.3%) | 9.8% (7.8%, 12.2%) |
| **Iran (Islamic Republic of)** | 39.2 (36.3, 42.3) | 37.7 (34.8, 40.9) | -0.2% (-0.3%, -0.2%) | -3.8% (-5%, -2.6%) |
| **Iraq** | 32 (28.7, 35.4) | 36 (32.7, 39.5) | 0.4% (0.4%, 0.4%) | 12.4% (8%, 17.1%) |
| **Ireland** | 42.9 (39.9, 46.3) | 40.3 (37.2, 43.7) | -0.3% (-0.3%, -0.2%) | -6.1% (-10.2%, -2.2%) |
| **Israel** | 47.9 (44.8, 51.2) | 41.7 (38.5, 44.9) | -0.6% (-0.7%, -0.5%) | -13.1% (-17.1%, -9.1%) |
| **Italy** | 48 (45.4, 50.9) | 44 (40.8, 47.1) | -0.4% (-0.4%, -0.3%) | -8.5% (-11.8%, -5.2%) |
| **Jamaica** | 28.8 (25.6, 32.2) | 30.9 (27.6, 34.4) | 0.2% (0.2%, 0.2%) | 7.3% (3%, 12%) |
| **Japan** | 40.8 (37.7, 43.9) | 41.2 (38.1, 44.5) | 0% (-0.1%, 0%) | 1.1% (-2.7%, 4.7%) |
| **Jordan** | 38.4 (35.2, 41.9) | 43.7 (40.5, 47) | 0.5% (0.4%, 0.5%) | 13.8% (8.8%, 19.4%) |
| **Kazakhstan** | 31 (27.8, 34.4) | 31.1 (27.7, 34.3) | -0.1% (-0.2%, -0.1%) | 0.1% (-3.9%, 4.3%) |
| **Kenya** | 15.8 (12.8, 19) | 17.1 (14.1, 20.4) | 0.2% (0.2%, 0.3%) | 8.2% (4%, 13.5%) |
| **Kiribati** | 27.4 (24.1, 30.8) | 28.1 (25, 31.4) | 0% (-0.1%, 0.1%) | 2.5% (-1.6%, 6.7%) |
| **Kuwait** | 41.1 (37.9, 44.5) | 45.3 (42.3, 48.6) | 0.5% (0.4%, 0.5%) | 10.3% (5.7%, 15.4%) |
| **Kyrgyzstan** | 31.3 (28, 34.7) | 29.8 (26.5, 33.1) | -0.2% (-0.3%, -0.2%) | -5.1% (-9.1%, -0.8%) |
| **Lao People's Democratic Republic** | 32.3 (29.1, 35.6) | 29.5 (26.3, 33) | -0.4% (-0.5%, -0.4%) | -8.6% (-12.6%, -4.7%) |
| **Latvia** | 53.1 (50, 56.2) | 47.4 (44.3, 50.5) | -0.5% (-0.6%, -0.5%) | -10.6% (-14.6%, -6.6%) |
| **Lebanon** | 41.3 (38.1, 44.6) | 47.1 (44, 50.3) | 0.5% (0.4%, 0.6%) | 14.1% (9.1%, 19.7%) |
| **Lesotho** | 10.9 (8.4, 13.6) | 12.6 (10, 15.5) | 0.5% (0.4%, 0.5%) | 16.1% (10%, 24.2%) |
| **Liberia** | 19.9 (16.8, 23.2) | 23.7 (20.5, 27.1) | 0.8% (0.7%, 0.9%) | 19% (13.5%, 26%) |
| **Libya** | 28.6 (25.3, 32.1) | 35.7 (32.6, 39) | 0.8% (0.7%, 0.8%) | 25% (19.5%, 31.9%) |
| **Lithuania** | 64.5 (61.8, 67.1) | 43.1 (39.9, 46.5) | -1.9% (-2.2%, -1.7%) | -33.2% (-36.8%, -29.3%) |
| **Luxembourg** | 51.9 (49.1, 55) | 45.8 (42.7, 49) | -0.5% (-0.6%, -0.4%) | -11.9% (-15.7%, -7.9%) |
| **Madagascar** | 18.3 (15.2, 21.6) | 19.5 (16.4, 22.9) | 0.1% (0%, 0.2%) | 6.5% (2.2%, 11%) |
| **Malawi** | 22.3 (19.2, 25.6) | 24.6 (21.5, 28) | 0.4% (0.3%, 0.4%) | 10.2% (5.6%, 15.1%) |
| **Malaysia** | 50.1 (47.2, 53.1) | 53.9 (50.9, 56.9) | 0.2% (0.2%, 0.2%) | 7.7% (2.9%, 12.1%) |
| **Maldives** | 38.6 (35.3, 42.1) | 40.7 (37.6, 44) | 0.1% (0%, 0.2%) | 5.4% (0.8%, 10.5%) |
| **Mali** | 18.4 (15.3, 21.6) | 21.3 (18.1, 24.7) | 0.6% (0.6%, 0.7%) | 16% (10.9%, 22.3%) |
| **Malta** | 55.5 (52.6, 58.4) | 51.3 (48, 54.4) | -0.3% (-0.3%, -0.3%) | -7.5% (-11.7%, -3.3%) |
| **Marshall Islands** | 31.3 (28.1, 34.8) | 34.7 (31.4, 38) | 0.3% (0.2%, 0.3%) | 11% (5.9%, 16.4%) |
| **Mauritania** | 23.2 (20.1, 26.7) | 26 (22.9, 29.4) | 0.4% (0.3%, 0.4%) | 11.8% (7.5%, 17.2%) |
| **Mauritius** | 43.2 (40, 46.5) | 43.1 (40, 46.3) | -0.1% (-0.1%, 0%) | -0.4% (-4.7%, 4.3%) |
| **Mexico** | 36.9 (33.7, 40.1) | 41.2 (38.1, 44.3) | 0.4% (0.3%, 0.4%) | 11.7% (7.3%, 16.9%) |
| **Micronesia (Federated States of)** | 31.5 (28.3, 35) | 33 (29.8, 36.4) | 0% (-0.1%, 0.1%) | 4.7% (0.7%, 8.9%) |
| **Monaco** | 56.5 (53.7, 59.3) | 54.1 (51.1, 57.4) | -0.2% (-0.2%, -0.2%) | -4.3% (-8.6%, -0.2%) |
| **Mongolia** | 35.3 (32.1, 38.7) | 38.9 (35.7, 42.2) | 0.4% (0.3%, 0.4%) | 10.2% (5.6%, 15.3%) |
| **Montenegro** | 48.1 (45.1, 51.3) | 44.6 (41.5, 47.8) | -0.3% (-0.4%, -0.3%) | -7.4% (-11.6%, -3.1%) |
| **Morocco** | 34.5 (31.3, 38) | 37 (33.9, 40.5) | 0.2% (0.2%, 0.3%) | 7.2% (3.1%, 11.9%) |
| **Mozambique** | 23.4 (20.2, 26.6) | 25.1 (21.9, 28.6) | 0.2% (0.2%, 0.3%) | 7.4% (3.3%, 12%) |
| **Myanmar** | 28 (24.8, 31.4) | 29.4 (26.3, 32.8) | 0.2% (0.2%, 0.2%) | 4.9% (0.9%, 9.3%) |
| **Namibia** | 19.6 (16.4, 22.9) | 20.4 (17.3, 23.8) | 0.1% (0%, 0.1%) | 4.4% (0.3%, 8.8%) |
| **Nauru** | 33.1 (29.8, 36.4) | 35.5 (32.3, 39.1) | 0.2% (0.1%, 0.2%) | 7.5% (3.1%, 12.2%) |
| **Nepal** | 20.1 (17, 23.5) | 23.4 (20.2, 26.7) | 0.6% (0.5%, 0.6%) | 16.4% (12.1%, 21.6%) |
| **Netherlands** | 58.8 (55.8, 61.9) | 48.9 (45.8, 52.2) | -0.6% (-0.8%, -0.4%) | -16.9% (-21.1%, -12.9%) |
| **New Zealand** | 53 (50.1, 56) | 48.7 (45.4, 51.9) | -0.4% (-0.4%, -0.3%) | -8.2% (-12.5%, -4.2%) |
| **Nicaragua** | 31 (27.9, 34.4) | 36.2 (32.9, 39.7) | 0.6% (0.5%, 0.6%) | 16.7% (12.1%, 22%) |
| **Niger** | 18.5 (15.4, 21.7) | 20.1 (17, 23.4) | 0.4% (0.3%, 0.4%) | 9.1% (4.8%, 14%) |
| **Nigeria** | 24.9 (21.8, 28.3) | 25.7 (22.6, 29.1) | 0% (0%, 0.1%) | 2.9% (-1.1%, 7.7%) |
| **Niue** | 27.7 (24.5, 31.2) | 29.9 (26.6, 33.3) | 0.2% (0.2%, 0.3%) | 7.7% (3.5%, 12.7%) |
| **North Macedonia** | 45 (41.9, 48.2) | 43 (39.7, 46.2) | -0.2% (-0.2%, -0.2%) | -4.5% (-8.8%, -0.1%) |
| **Northern Mariana Islands** | 33.7 (30.6, 37) | 35.8 (32.7, 39.3) | 0.1% (0%, 0.2%) | 6.3% (1.8%, 11.1%) |
| **Norway** | 54.1 (51.1, 57.2) | 52.6 (49.5, 55.7) | -0.2% (-0.2%, -0.1%) | -2.9% (-7%, 1.4%) |
| **Oman** | 36.1 (33, 39.5) | 46 (42.8, 49.3) | 0.8% (0.8%, 0.9%) | 27.3% (20.9%, 34.9%) |
| **Pakistan** | 24.3 (21.1, 27.7) | 27.8 (24.6, 31.2) | 0.5% (0.5%, 0.5%) | 14.5% (9.6%, 20%) |
| **Palau** | 32.1 (28.9, 35.5) | 35.5 (32.3, 39) | 0.3% (0.2%, 0.3%) | 10.4% (5.7%, 15.6%) |
| **Palestine** | 38.2 (35, 41.6) | 39.6 (36.3, 42.9) | -0.1% (-0.2%, 0.1%) | 3.7% (-0.6%, 8.4%) |
| **Panama** | 32.9 (29.7, 36.4) | 37.8 (34.6, 41.1) | 0.5% (0.5%, 0.5%) | 14.8% (10.1%, 20.2%) |
| **Papua New Guinea** | 27.7 (24.5, 31.1) | 28.6 (25.5, 32.1) | 0% (0%, 0.1%) | 3.3% (-0.6%, 7.5%) |
| **Paraguay** | 39 (35.7, 42.4) | 43.1 (39.9, 46.4) | 0.3% (0.3%, 0.4%) | 10.4% (5.8%, 15.2%) |
| **Peru** | 31.3 (28, 34.8) | 35 (31.9, 38.4) | 0.5% (0.4%, 0.5%) | 11.9% (7.4%, 17.4%) |
| **Philippines** | 35.1 (31.9, 38.6) | 37 (33.7, 40.2) | 0.1% (0.1%, 0.2%) | 5.4% (0.9%, 10%) |
| **Poland** | 45.1 (42.1, 48.1) | 39.1 (36, 42.4) | -0.6% (-0.7%, -0.5%) | -13.3% (-16.7%, -9.7%) |
| **Portugal** | 49.3 (46.2, 52.3) | 49.9 (46.7, 53.3) | 0% (0%, 0%) | 1.3% (-2.8%, 5.8%) |
| **Puerto Rico** | 35.1 (31.8, 38.8) | 38.4 (35.1, 41.7) | 0.3% (0.3%, 0.3%) | 9.2% (4.6%, 14.4%) |
| **Qatar** | 32.9 (29.7, 36.3) | 33.3 (30.1, 36.7) | -0.1% (-0.2%, 0%) | 1.3% (-3.2%, 6.4%) |
| **Republic of Korea** | 23.9 (20.7, 27.3) | 35.5 (32.3, 38.8) | 0.6% (0.4%, 0.9%) | 48.6% (40.1%, 59.2%) |
| **Republic of Moldova** | 31.9 (28.6, 35.3) | 31.2 (27.8, 34.5) | -0.1% (-0.2%, 0%) | -2.2% (-6.3%, 1.6%) |
| **Romania** | 47 (44, 50.2) | 48.9 (45.7, 52.1) | 0.1% (0.1%, 0.1%) | 4.1% (-0.4%, 8.6%) |
| **Russian Federation** | 47 (44.2, 50) | 49.3 (46.3, 52.3) | 0.2% (0.2%, 0.2%) | 4.9% (0.8%, 9.4%) |
| **Rwanda** | 9.7 (7.3, 12.4) | 10.3 (7.8, 13) | 0.2% (0.2%, 0.2%) | 5.6% (-0.3%, 12.7%) |
| **Saint Kitts and Nevis** | 31 (27.7, 34.4) | 32.9 (29.7, 36.3) | 0.2% (0.2%, 0.2%) | 6.3% (2.2%, 11.3%) |
| **Saint Lucia** | 23 (19.9, 26.2) | 25.3 (22.3, 28.6) | 0.3% (0.3%, 0.3%) | 10.2% (5.8%, 15%) |
| **Saint Vincent and the Grenadines** | 23.2 (20.1, 26.6) | 25.4 (22.3, 28.9) | 0.3% (0.3%, 0.3%) | 9.5% (5.2%, 15%) |
| **Samoa** | 24.5 (21.2, 27.8) | 24.2 (21.2, 27.5) | -0.1% (-0.1%, -0.1%) | -1% (-4.7%, 3%) |
| **San Marino** | 54.3 (51.4, 57.5) | 51.5 (48.5, 54.6) | -0.2% (-0.2%, -0.2%) | -5.2% (-9.4%, -0.4%) |
| **Sao Tome and Principe** | 20.6 (17.6, 24) | 24.4 (21.2, 27.7) | 0.7% (0.6%, 0.7%) | 18.3% (13.1%, 24.4%) |
| **Saudi Arabia** | 24 (20.9, 27.4) | 35.3 (32.1, 38.7) | 1.3% (1.2%, 1.4%) | 47.1% (38.5%, 57.7%) |
| **Senegal** | 21.4 (18.3, 24.8) | 22.9 (19.7, 26.3) | 0.2% (0.2%, 0.2%) | 6.9% (2.5%, 11.9%) |
| **Serbia** | 54.3 (51.4, 57.2) | 57.3 (54.3, 60.4) | 0.1% (0.1%, 0.2%) | 5.6% (1.3%, 10.1%) |
| **Seychelles** | 42.2 (39.1, 45.2) | 44.9 (41.7, 48.2) | 0.2% (0.1%, 0.3%) | 6.5% (1.9%, 11.3%) |
| **Sierra Leone** | 19.2 (16.1, 22.6) | 22 (18.8, 25.3) | 0.5% (0.5%, 0.6%) | 14.4% (9.7%, 19.9%) |
| **Singapore** | 50.2 (47.4, 53.2) | 42.9 (39.9, 46.3) | -0.8% (-0.9%, -0.7%) | -14.6% (-18.3%, -10.7%) |
| **Slovakia** | 49.4 (46.3, 52.6) | 47.4 (44.2, 50.6) | -0.3% (-0.3%, -0.2%) | -4.2% (-8.5%, 0.2%) |
| **Slovenia** | 50.4 (47.4, 53.6) | 48.5 (45.5, 51.6) | -0.2% (-0.2%, -0.2%) | -3.9% (-8.1%, 0.4%) |
| **Solomon Islands** | 26.2 (22.9, 29.4) | 28.2 (25, 31.7) | 0.2% (0.1%, 0.3%) | 7.8% (3.5%, 12.3%) |
| **Somalia** | 16.8 (13.8, 20) | 17 (14, 20.2) | -0.1% (-0.2%, 0%) | 0.9% (-3.5%, 5.1%) |
| **South Africa** | 24.8 (21.6, 28.1) | 27.3 (24.2, 30.6) | 0.4% (0.3%, 0.4%) | 10% (5.8%, 15.2%) |
| **South Sudan** | 17.8 (14.8, 21.1) | 19.4 (16.3, 22.7) | 0.2% (0.1%, 0.3%) | 8.7% (4.4%, 14%) |
| **Spain** | 48.1 (45.1, 51.2) | 40.2 (37.1, 43.4) | -1% (-1.2%, -0.9%) | -16.5% (-20.3%, -12.8%) |
| **Sri Lanka** | 41.5 (38.4, 44.7) | 44.2 (40.9, 47.5) | 0.2% (0.2%, 0.2%) | 6.4% (1.9%, 11%) |
| **Sudan** | 22.7 (19.4, 26.1) | 27.1 (23.9, 30.6) | 0.6% (0.6%, 0.7%) | 19.3% (14.2%, 25.5%) |
| **Suriname** | 34.8 (31.5, 38.1) | 39.2 (36.1, 42.6) | 0.5% (0.5%, 0.5%) | 12.7% (8.5%, 17.8%) |
| **Sweden** | 58.9 (56.2, 61.6) | 45.4 (42.2, 48.6) | -1% (-1.2%, -0.9%) | -22.8% (-26.7%, -19.4%) |
| **Switzerland** | 60.8 (58.1, 63.4) | 49 (45.9, 52) | -0.9% (-1%, -0.8%) | -19.3% (-23%, -15.4%) |
| **Syrian Arab Republic** | 35.2 (31.9, 38.7) | 38.3 (35, 41.6) | 0.2% (0.2%, 0.3%) | 8.6% (4%, 13.7%) |
| **Taiwan (Province of China)** | 42.9 (39.9, 46.1) | 49.3 (46.3, 52.4) | 0.5% (0.2%, 0.7%) | 14.8% (9.9%, 20.3%) |
| **Tajikistan** | 28.9 (25.6, 32.2) | 28.3 (25.2, 31.8) | -0.1% (-0.1%, -0.1%) | -1.9% (-5.7%, 1.8%) |
| **Thailand** | 43.9 (40.7, 47.2) | 45.6 (42.4, 49) | 0% (0%, 0.1%) | 3.9% (-0.2%, 8.7%) |
| **Timor-Leste** | 24.3 (21, 27.6) | 23.8 (20.6, 27.2) | -0.1% (-0.1%, 0%) | -1.9% (-5.8%, 2%) |
| **Togo** | 23.1 (19.9, 26.4) | 26 (22.9, 29.5) | 0.4% (0.4%, 0.4%) | 12.5% (8%, 18.1%) |
| **Tokelau** | 29.7 (26.6, 33.2) | 33.3 (30.2, 36.6) | 0.4% (0.3%, 0.4%) | 12.1% (7.4%, 17.3%) |
| **Tonga** | 34.3 (31, 37.8) | 38.4 (35.3, 41.8) | 0.3% (0.2%, 0.4%) | 11.9% (7.3%, 17.4%) |
| **Trinidad and Tobago** | 35.9 (32.6, 39.2) | 39.3 (36.1, 42.7) | 0.4% (0.3%, 0.4%) | 9.6% (5.2%, 14.3%) |
| **Tunisia** | 34.6 (31.3, 37.9) | 37.8 (34.6, 41.2) | 0.3% (0.3%, 0.3%) | 9.5% (4.9%, 14.4%) |
| **Turkey** | 36.1 (32.9, 39.4) | 30.9 (27.8, 34.2) | -0.4% (-0.6%, -0.1%) | -14.4% (-18%, -11%) |
| **Turkmenistan** | 31.8 (28.7, 35) | 33.2 (29.9, 36.5) | 0.2% (0.1%, 0.2%) | 4.2% (0.1%, 8.7%) |
| **Tuvalu** | 29.1 (25.8, 32.4) | 31.8 (28.7, 35.2) | 0.2% (0.2%, 0.3%) | 9.5% (5.2%, 14.7%) |
| **Uganda** | 13.7 (10.9, 16.8) | 15.3 (12.5, 18.2) | 0.4% (0.4%, 0.5%) | 11.6% (6.2%, 18.2%) |
| **Ukraine** | 52.8 (49.7, 55.9) | 44.8 (41.8, 48) | -0.7% (-0.7%, -0.6%) | -15% (-18.9%, -11.1%) |
| **United Arab Emirates** | 40.8 (37.5, 44.2) | 49.8 (46.7, 53.1) | 0.8% (0.7%, 0.8%) | 22.1% (15.8%, 29.6%) |
| **United Kingdom** | 56.2 (53.8, 58.6) | 43.5 (40.7, 46.5) | -1% (-1.1%, -0.9%) | -22.6% (-24.9%, -20.3%) |
| **United Republic of Tanzania** | 20.3 (17.1, 23.6) | 28.8 (25.6, 32) | 1.1% (0.9%, 1.2%) | 41.6% (33.6%, 52.4%) |
| **United States of America** | 67 (64.8, 69.2) | 31.2 (28.1, 34.5) | -2.8% (-3.2%, -2.5%) | -53.4% (-57.5%, -49.3%) |
| **United States Virgin Islands** | 33 (29.9, 36.4) | 36.1 (33, 39.4) | 0.3% (0.3%, 0.3%) | 9.3% (4.8%, 14.2%) |
| **Uruguay** | 42.5 (39.2, 45.8) | 47 (43.8, 50.3) | 0.4% (0.3%, 0.4%) | 10.5% (6.3%, 15.1%) |
| **Uzbekistan** | 34.4 (31.1, 37.8) | 37.6 (34.5, 41) | 0.3% (0.2%, 0.3%) | 9.4% (5%, 14.2%) |
| **Vanuatu** | 30.9 (27.7, 34.3) | 34.6 (31.3, 37.8) | 0.4% (0.4%, 0.4%) | 11.7% (7.3%, 16.7%) |
| **Venezuela (Bolivarian Republic of)** | 27.7 (24.6, 31.1) | 29.5 (26.2, 32.9) | 0.2% (0.2%, 0.2%) | 6.4% (1.9%, 10.7%) |
| **Viet Nam** | 29.8 (26.6, 33.3) | 29 (25.9, 32.5) | -0.2% (-0.2%, -0.1%) | -2.6% (-6.6%, 1.6%) |
| **Yemen** | 30.3 (27.1, 33.7) | 33.9 (30.7, 37.3) | 0.4% (0.4%, 0.5%) | 11.6% (7.1%, 16.7%) |
| **Zambia** | 18.7 (15.7, 22.2) | 20.4 (17.3, 23.7) | 0.2% (0.1%, 0.3%) | 9% (4.5%, 14%) |
| **Zimbabwe** | 20.8 (17.7, 24.1) | 21.9 (18.8, 25.2) | 0.1% (0%, 0.1%) | 5.5% (1.5%, 10.2%) |

Abbreviations: ASR, age-standardized rate; UI, uncertainty interval; EAPC, estimated annual percentage change; SEV, summary exposure value.

**Supplementary Table 6 Top 3 countries/territories of death and DALY in different scales (absolute number, changes in age-standardized rates from 1990 to 2019, changes in EAPC of age-standardized rates from 1990 to 2019).**

| **Ranks** | **1** | **2** | **3** |
| --- | --- | --- | --- |
| **Death number in 2019 (number, 95% UI)** | China (915983.1, 95% UI 647993.1, 1239381.7) | India (630093.2, 95% UI 474415.3, 803890.3) | Russian Federation (332216.9, 95% UI 241611.8, 435259.4) |
| **DALY number in 2019 (number, 95% UI)** | China (19813961.9, 95% UI 15205317.0,25139359.0) | India (17679116.3, 95% UI, 13917214.0, 21933658.9) | Russian Federation (6388323.2, 95% UI 4897067.2, 7947908.9) |
| **Increase in age-standardized death rate from 1990 to 2019 (%, 95% UI)** | Uzbekistan (110.1%, 95% UI 82.5%, 137.4%) | Tajikistan (70.4%, 95% UI 38.9%, 105.3%) | Lesotho (65.7%, 95% UI 23.2%, 121.5%) |
| **Increase in age-standardized DALY rate from 1990 to 2019 (%, 95% UI)** | Philippines (88.4%, 95% UI 30.2%, 138.4%) | Uzbekistan (81.9%, 95% UI 55.2%, 109.2%) | Lesotho (74.9%, 95% UI 26.1%,140.6%) |
| **Increase in EAPC of age-standardized death rate from 1990 to 2019 (%, 95% CI)** | Uzbekistan (2.8%, 95% CI 2.1%, 3.4%) | Lesotho (2.5%, 95% CI 2.1%, 2.8%) | Philippines (2.2%, 95% CI 1.7%, 2.7%) |
| **Increase in EAPC of age-standardized DALY rate from 1990 to 2019 (%, 95% CI)** | Philippines (3.0%, 95% CI 2.4%, 3.7%) | Lesotho (2.7%, 95% CI 2.3%, 3.1%) | Uzbekistan (2.2%, 95% CI 1.6%, 2.8%) |
| **Decrease in age-standardized death rate from 1990 to 2019 (%, 95% UI)** | Denmark (-74.6%, 95% UI -77.5%, -71.8%) | Israel (-74.2%, 95% UI -77.0%, -71.5%) | Luxembourg (-70.6%, 95% UI -75.1%, -66.2%) |
| **Decrease in age-standardized DALY rate from 1990 to 2019 (%, 95% UI)** | Denmark (-77.0%, 95% UI -79.1%, -74.9%) | Israel (-75.8%, 95% UI -77.7%, -73.7%) | Republic of Korea (–73.4%, 95% UI -76.9%, -67.2%) |
| **Decrease in EAPC of age-standardized death rate from 1990 to 2019 (%, 95% CI)** | Denmark (-5.4%, 95% CI -5.8%, -5.1%) | Israel (-5.2%, 95% CI -5.6%, -4.9%) | Netherlands (-4.6%, 95% CI -4.9%, -4.3%) |
| **Decrease in EAPC of age-standardized DALY rate from 1990 to 2019 (%, 95% CI)** | Denmark (-5.7%, 95% CI -6.0%, -5.3%) | Israel (-5.4%, 95% CI -5.8%, -5.0%) | Netherlands (-5.0%, 95% CI -5.3%, -4.7%) |

Abbreviations: DALY, disability adjusted life year; UI, uncertainty interval; EAPC, estimated annual percentage change.

# Supplementary Table 7 High LDL-C attributable IHD death and DALY in different geographic region

| **Characteristics** | **1990 Death No. (95%UI)** | **1990 age-standardized Death rate per 100,000 No. (95%UI)** | **2019 Death No. (95%UI)** | **2019 age-standardized Death rate per 100,000 No. (95%UI)** | **1990 DALY No. (95%UI)** | **1990 age-standardized DALY rate per 100,000 No. (95%UI)** | **2019 DALY No. (95%UI)** | **2019 age-standardized DALY rate per 100,000 No. (95%UI)** |
| --- | --- | --- | --- | --- | --- | --- | --- | --- |
| **Overall** | 2588048.9 (2017002.8, 3185565.4) | 76 (56.7, 97.2) | 3784333.2 (2826645.1, 4824700) | 48.4 (35.5, 63) | 60433790.2 (50462005.4, 70729727.5) | 1530.7 (1263.9, 1819.6) | 84916393.7 (69572553.4, 101382483.1) | 1036.9 (843.8, 1245) |
| **Sex** | | | | | | | | |
| **Female** | 1190459.3 (878390.7, 1529698) | 63.1 (45.2, 82.6) | 1708932.1 (1182566.2, 2257842.8) | 39 (27.1, 51.5) | 23331944 (19018848.1, 28041483.3) | 1125.7 (904.7, 1377.2) | 32109848.2 (25174177.4, 39554942.6) | 739.6 (580.8, 910.2) |
| **Male** | 1397589.6 (1127757.5, 1678645.7) | 90.4 (68.6, 112.7) | 2075401.2 (1616596.6, 2576373.9) | 58.7 (44.2, 74.5) | 37101846.2 (31655230.8, 42781041.5) | 1955.6 (1621.6, 2299.2) | 52806545.5 (43570004, 62730862.3) | 1347.2 (1103, 1610.9) |
| **Socio-demographic index** | | | | | | | | |
| **High SDI** | 97952.1 (74979.2, 125439.3) | 65.2 (49, 83.5) | 1164826.1 (896797.9, 1479269.4) | 64 (47.9, 82.3) | 13494227.2 (11411467.9, 15931370.1) | 1236 (1006.9, 1481.7) | 10184485.3 (7968781.7, 12498619.2) | 584 (478.4, 695.5) |
| **High-middle SDI** | 849060.1 (633160.6, 1070940.5) | 59.5 (45.1, 75.5) | 210272.4 (161377.8, 267112.4) | 51 (36.8, 66.4) | 15999090.2 (13117419.8, 18857382.8) | 1574.8 (1302.9, 1839.9) | 28092038.6 (23053024, 33715982.7) | 1118.6 (903.4, 1355.9) |
| **Middle SDI** | 487037.6 (388529, 593160.8) | 52.7 (38.6, 69.1) | 644255 (495220.4, 810304.5) | 60.5 (44.8, 77.6) | 8485977.5 (6998959.7, 10199479.5) | 1270.3 (1027, 1557.1) | 6109229.1 (4829457.8, 7559915.3) | 1040.1 (811.4, 1304.7) |
| **Low-middle SDI** | 282721.7 (225113.8, 348827.6) | 110.5 (85, 135.6) | 1160901.9 (844417, 1497306.3) | 71.3 (52.9, 90.9) | 19497749.5 (16339365.1, 22871682.7) | 1868.4 (1544.8, 2219.2) | 17649413.8 (14224429, 21392306.6) | 1214.8 (964.2, 1495.9) |
| **Low SDI** | 869891 (672551.1, 1069410.5) | 114.4 (87.3, 143.5) | 601931.8 (410907.4, 815844.4) | 38.5 (28.5, 49.1) | 2923658 (2323275.5, 3629760.3) | 1108.3 (855.7, 1406.1) | 22829341.7 (18408904.2, 27442591.9) | 1145.1 (922.7, 1377.3) |
| **Geographic regions** | | | | | | | | |
| **Andean Latin America** | 7936.9 (6019.1, 10281.5) | 42.4 (30.8, 56.7) | 462920.4 (335671, 594465.2) | 134.7 (98.7, 172.2) | 1300279.3 (1062494.2, 1553445.6) | 665.1 (535.6, 802) | 15936514.4 (12452843.2, 20002040.6) | 816.4 (631.7, 1033) |
| **Australasia** | 54230.1 (41499.3, 67743) | 127.4 (94.5, 164.1) | 228143.9 (159504.7, 309545.2) | 34.4 (24.8, 45.3) | 7230614.4 (5876931.4, 8856688.2) | 810.2 (643.7, 999) | 230431.6 (177324.9, 284705.1) | 484.1 (389.7, 582.4) |
| **Caribbean** | 62820 (46318.3, 80474.2) | 35.5 (25.3, 46.8) | 27205.8 (19916.6, 34937.8) | 32.3 (23.9, 41.2) | 76835.1 (59228.5, 100599.2) | 2113.5 (1615.5, 2776.8) | 196760.6 (149869.2, 258295.5) | 2234.8 (1703.2, 2955.1) |
| **Central Asia** | 105420.3 (83643.1, 129528.1) | 46 (34.7, 59) | 757237.6 (540120.8, 1014043.7) | 44.2 (29.7, 61.3) | 3982741.4 (3311781.6, 4642654) | 2803.6 (2324.5, 3276.2) | 4352379 (3308737.4, 5458304.4) | 497.4 (401.6, 600.2) |
| **Central Europe** | 39591.7 (30662.1, 49045.2) | 53.2 (39, 68.5) | 94817.3 (68203, 124014.8) | 41.5 (29.4, 54.3) | 374752.6 (305764, 446203.2) | 1644 (1343.5, 1948.4) | 2062485.6 (1671029.8, 2502475.6) | 2766.9 (2153.1, 3416.5) |
| **Central Latin America** | 28524.9 (21378.4, 35978.5) | 68.6 (50.2, 88.5) | 71136.4 (45310.6, 100510.8) | 13.2 (9.4, 17.7) | 3021939.4 (2514365.2, 3627867.5) | 1066.9 (864.5, 1299) | 1055516.8 (789778.7, 1353922.3) | 260.2 (212.6, 313.6) |
| **Central Sub-Saharan Africa** | 385022.9 (296531.4, 478279.9) | 156 (115.8, 198.1) | 25973.6 (18684.2, 34009.1) | 49.9 (35.9, 65.3) | 243467.6 (180334, 324667.9) | 979.9 (715.1, 1320.6) | 298766.2 (229855, 385696.2) | 523 (399.3, 678) |
| **East Asia** | 17679.8 (13442.5, 22381.5) | 73.2 (54.2, 94.2) | 15044.4 (10083.1, 20406.6) | 27.6 (19.3, 36.5) | 8229579.5 (6676564.6, 9779589.1) | 1484.8 (1227.4, 1742) | 6660614.1 (5400826.7, 7999063.6) | 1024.1 (819.8, 1244.3) |
| **Eastern Europe** | 19162.7 (14376.9, 23941) | 86 (64.3, 108.2) | 295254.9 (194342, 404661.4) | 27.8 (19.5, 36.9) | 412744.3 (336400.7, 493467.7) | 1573.8 (1268.8, 1889.1) | 4100410.4 (3247570.4, 5030593.1) | 706.2 (575, 848) |
| **Eastern Sub-Saharan Africa** | 260116 (203197.2, 325909.8) | 38 (27.6, 51.1) | 39096.8 (26773.3, 52707) | 27.6 (17.6, 39.2) | 1571089.2 (1354925.1, 1799518.9) | 1608.1 (1345, 1872.5) | 1501226.2 (1111360.6, 1957120.5) | 750.9 (544.1, 991.2) |
| **High-income Asia Pacific** | 349340.4 (261537.9, 435622.6) | 97.9 (74.7, 120.6) | 17717.6 (12003.9, 24890.6) | 38.6 (24.5, 56.7) | 745206.3 (538768.3, 1030297.4) | 829.4 (588.5, 1153) | 9194541.4 (7426050.6, 11301281.2) | 1960.8 (1547.6, 2440.1) |
| **High-income North America** | 17797.4 (12961.7, 23597.3) | 27.5 (18.9, 38) | 14062.5 (9685, 19157.2) | 25.9 (17.5, 35.8) | 602810.9 (493390.7, 716789.9) | 1339.1 (1090.1, 1600.3) | 582868.9 (455903.5, 730187.4) | 1126.9 (881.7, 1411.9) |
| **North Africa and Middle East** | 449770.4 (330592.2, 572298.6) | 78.2 (58.4, 98.6) | 155660.6 (104952.2, 208532.1) | 71.9 (49.1, 95.4) | 192318.4 (156619.5, 232308.1) | 882.8 (707.2, 1092.8) | 2035568.8 (1630878.9, 2518143.6) | 847.2 (671.5, 1056.3) |
| **Oceania** | 188145.8 (144130.1, 230785.6) | 143.4 (107.3, 181.6) | 85508.4 (65030.2, 108548.8) | 141.3 (100.4, 187.3) | 6510577.2 (5368205.6, 7635363.8) | 1924.9 (1615.2, 2233) | 2106720.6 (1778602.5, 2430616.8) | 849.3 (712.5, 984.9) |
| **South Asia** | 58036.7 (47062.6, 69777.4) | 71.4 (54.7, 89) | 247909 (192546, 310157.6) | 44.6 (32.7, 57.7) | 1252517.5 (1037257.8, 1479457.6) | 2643.1 (2155.5, 3170.5) | 500195 (343243.1, 689140.8) | 844.2 (580, 1179.9) |
| **Southeast Asia** | 197136.4 (157544, 239228.6) | 127.7 (96.2, 161.6) | 16562.1 (12249.4, 21096.9) | 33.4 (23.7, 44.1) | 976671.4 (822596.9, 1139122.9) | 1105.2 (900.5, 1318.1) | 522137.1 (428187.2, 620500.2) | 641.9 (531, 758.7) |
| **Southern Latin America** | 2315.2 (1759, 3052.3) | 78.4 (57.6, 104.9) | 6014.9 (4554.6, 8050.4) | 84.9 (62.7, 115.2) | 8111230.4 (6790121.1, 9532090.4) | 3023.3 (2505.1, 3556.1) | 416979.4 (326817.8, 510626.3) | 709 (542.8, 880.2) |
| **Southern Sub-Saharan Africa** | 299160 (236909.8, 370779.6) | 56.4 (42.9, 73.3) | 86015.1 (67440.4, 104145.4) | 36.1 (27.9, 44.3) | 5438464.1 (4534746.3, 6382465.3) | 2924 (2389.4, 3487.6) | 1092055.3 (785597.3, 1437960.4) | 602.8 (419.9, 800.7) |
| **Tropical Latin America** | 8334.4 (5990.7, 11324.9) | 42.7 (29.1, 60.7) | 58597.1 (41624.2, 78794.4) | 37.1 (24.9, 51.3) | 226084.1 (183398.1, 272972.4) | 752.4 (595.1, 926.7) | 8803759 (6920749.3, 10715721.2) | 2644.4 (2102.8, 3194.6) |
| **Western Europe** | 29385.3 (20360.2, 41108.8) | 40.1 (26.4, 57.6) | 722302.2 (548950.9, 911615) | 54.8 (40.2, 70.6) | 518780 (394861.6, 677466.6) | 633 (469.5, 832.2) | 2618149.4 (1952194.9, 3300961) | 1288 (980.6, 1597.8) |
| **Western Sub-Saharan Africa** | 8121.6 (6223.4, 10259.8) | 31.8 (23, 41.7) | 357152.5 (270091.6, 447610.3) | 91.1 (66.5, 118.7) | 9415086.9 (7741200.1, 11414726.7) | 1423 (1137.8, 1758.9) | 20648314 (16277213.8, 25194013.8) | 1362.6 (1063.7, 1679.8) |

Abbreviations: IHD, ischemic heart disease; DALY, disability adjusted life year; ASR, age-standardized rate; UI, uncertainty interval.

# Supplementary Table 8 High LDL-C attributable stroke death and DALY in different geographic region

| **Characteristics** | **1990 Death No. (95%UI)** | **1990 age-standardized Death rate per 100,000 No. (95%UI)** | **2019 Death No. (95%UI)** | **2019 age-standardized Death rate per 100,000 No. (95%UI)** | **1990 DALY No. (95%UI)** | **1990 age-standardized DALY rate per 100,000 No. (95%UI)** | **2019 DALY No. (95%UI)** | **2019 age-standardized DALY rate per 100,000 No. (95%UI)** |
| --- | --- | --- | --- | --- | --- | --- | --- | --- |
| **Overall** | 414562 (160213.3, 836789.9) | 13.2 (4.6, 27.3) | 612650 (229107.4, 1276394.5) | 8.7 (3.2, 18.2) | 9285107.8 (5333877.5, 15649509.4) | 249.2 (130.6, 438.6) | 13701627.1 (7719835.2, 23430310.9) | 170.2 (92.8, 294.7) |
| **Sex** | | | | | | | | |
| **Female** | 239347.3 (79594.4, 503226) | 13.1 (4, 28.1) | 328087.8 (102589.1, 702486.1) | 7.5 (2.4, 16) | 4959152.8 (2653695, 8558309.2) | 242.5 (120.8, 431.1) | 6894345.5 (3602162.5, 12176622.2) | 158.7 (83.6, 279) |
| **Male** | 175214.7 (81595.7, 329146.6) | 13.3 (4.3, 28) | 284562.2 (119661.6, 566513.1) | 8.1 (2.8, 17) | 4325955 (2646074.4, 6940997) | 252.1 (134.8, 439.1) | 6807281.6 (4048313.5, 11274207.7) | 181.5 (101.8, 317.5) |
| **Socio-demographic index** | | | | | | | | |
| **High SDI** | 76827.7 (36694.6, 144353.3) | 10.2 (3.6, 21.8) | 23004.8 (10492.9, 45473.4) | 5.9 (2, 13.2) | 2099098.3 (945811.2, 3899495.5) | 202.8 (95.7, 368.1) | 1651784.3 (737661.5, 3154114.2) | 89.6 (47, 155) |
| **High-middle SDI** | 116747.9 (33302.4, 251196.3) | 11.3 (3.3, 24.4) | 190151.8 (79938.8, 382274.6) | 9.3 (3.3, 20.1) | 2159683.4 (1359472.3, 3406007.7) | 215.8 (120.1, 375.2) | 4545771.6 (2402390.4, 7983761.4) | 227 (120, 396.9) |
| **Middle SDI** | 32317.8 (14680.2, 62589.5) | 7.8 (2.6, 17) | 90996.6 (22234.9, 207697.6) | 3.9 (1.1, 8.7) | 886896.9 (534345.7, 1426563.8) | 155.7 (80.7, 278.7) | 668529.7 (421922.7, 1037987.2) | 126 (67.8, 220) |
| **Low-middle SDI** | 178994 (69886.7, 359895.2) | 20.6 (7, 43) | 227020.2 (78182.9, 470954.8) | 11.6 (3.9, 24.2) | 3852717.8 (2224279.9, 6597011) | 380.6 (201, 671) | 2030112.8 (1220798.6, 3332983.7) | 152.7 (83.8, 267.2) |
| **Low SDI** | 9497.4 (4193.1, 18388.2) | 5.9 (1.9, 13) | 81175.7 (34742.8, 162466.6) | 7.4 (2.6, 15.7) | 282861.4 (170563.6, 474718) | 123 (62.5, 220.1) | 4798555.3 (2868050.8, 8004203.2) | 199.4 (108.6, 352.7) |
| **Geographic regions** | | | | | | | | |
| **Andean Latin America** | 70757.8 (35147.8, 136058.8) | 11.1 (4.1, 23.2) | 2299 (453.6, 5506.8) | 3.8 (0.8, 9.1) | 668208.7 (372477.8, 1158475.7) | 471.5 (252.2, 830.2) | 223137.7 (143999.9, 341875) | 300.5 (172.3, 512.8) |
| **Australasia** | 29043.3 (7892.9, 61692.8) | 7.8 (2.2, 16.5) | 53233.1 (11026.1, 123640.4) | 4.4 (1.1, 10) | 5025.2 (3292.6, 7721.1) | 159.7 (89.1, 281.1) | 375884.5 (144117.4, 769982.2) | 83 (42.4, 146) |
| **Caribbean** | 18977.1 (8112.3, 36868.8) | 10.2 (3.4, 22.5) | 317.9 (163.2, 570.2) | 6.2 (2.2, 13.2) | 2034145.6 (1285437.3, 3307953.4) | 240.5 (136.4, 417.3) | 4575119.3 (2517839.4, 7948480) | 231.7 (120.1, 411.9) |
| **Central Asia** | 19065.9 (5756.3, 41641.8) | 11.7 (3.2, 26.4) | 22260 (4566.1, 53204.6) | 3.5 (0.9, 7.9) | 374316.5 (184791.3, 677805.4) | 200.6 (90.7, 379.5) | 76219.1 (35448.2, 143188.2) | 91.5 (44.5, 166.3) |
| **Central Europe** | 2094.3 (543.1, 4667.1) | 10 (2.5, 22.2) | 31856.9 (9047.6, 69597.5) | 14.1 (4.2, 30.5) | 35598.1 (15213.2, 69727.8) | 158.7 (67.6, 305.9) | 1482442.8 (788383.2, 2602620.1) | 434.7 (238.7, 751.5) |
| **Central Latin America** | 3753.5 (1291.8, 7930.8) | 9.4 (2.9, 20.5) | 79642.2 (28845.4, 163293.5) | 22.7 (8.3, 46.4) | 30385.5 (17827.6, 52871.4) | 142.6 (69.9, 266.8) | 550079.6 (245751.1, 1029376.9) | 261.6 (130, 467.3) |
| **Central Sub-Saharan Africa** | 31915.6 (12191, 64186) | 24.8 (8.5, 51.3) | 15984.5 (5637.3, 33618.6) | 7.1 (2.4, 15.2) | 75543.1 (38727.4, 137145.4) | 171.4 (83.6, 315.8) | 731614.9 (248701.8, 1502274.6) | 75.2 (34.3, 141) |
| **East Asia** | 127.3 (62.3, 236.5) | 6.1 (2.1, 13.6) | 4288.9 (1182.9, 9502.6) | 5 (1.4, 10.9) | 1767323.5 (1008203.4, 3020501.3) | 650.8 (352.3, 1129.8) | 1334177 (807318, 2182194.5) | 225.1 (121.3, 392.9) |
| **Eastern Europe** | 1936.6 (755.2, 4005.6) | 8.4 (3, 17.9) | 3443.2 (1282.8, 7340.1) | 8.1 (2.4, 18.9) | 110490.7 (69109.9, 176280.6) | 130.5 (71.5, 229) | 33193.6 (12351.9, 67391.2) | 66 (29.2, 125.2) |
| **Eastern Sub-Saharan Africa** | 11494.6 (5328.3, 22038.6) | 16.3 (6, 33.5) | 51253.6 (21387.1, 103865.9) | 10.6 (3.6, 23.5) | 150197.7 (93475.6, 239159) | 315.7 (183.5, 538.1) | 188261.9 (112038.3, 309851.5) | 115.2 (57.4, 216.9) |
| **High-income Asia Pacific** | 80044.8 (20726.5, 178376.4) | 13.6 (3.6, 30.1) | 44064.2 (21234.7, 81159.6) | 12.2 (4.7, 25.1) | 575113.7 (258423, 1070846.6) | 107.6 (58.3, 186.4) | 1416338.2 (824670.2, 2351401.4) | 103.9 (55.3, 181.3) |
| **High-income North America** | 4462.6 (1811.2, 8930.9) | 6.6 (2.2, 14.2) | 188178.1 (73577.7, 392766.9) | 10.9 (3.6, 23.5) | 22668.2 (13632, 36119.7) | 146.1 (77.9, 256.9) | 180525 (100529.8, 305926.3) | 74.1 (37.8, 132) |
| **North Africa and Middle East** | 6008.6 (2740.5, 11472.5) | 14.3 (5.7, 28.7) | 8170.7 (3647.1, 15985) | 5.9 (2, 13) | 40865.8 (25045.7, 65549.7) | 212.4 (91, 402.7) | 83764.5 (49061.1, 141644.3) | 76.9 (41, 135.9) |
| **Oceania** | 16779.5 (8186.4, 31123) | 12.8 (4.7, 27.2) | 20640.4 (4661.4, 50466.3) | 2.8 (0.7, 6.7) | 1241793.7 (484816.8, 2434426) | 215.3 (113.7, 384.4) | 441598.5 (201332.1, 847263.8) | 156 (78.2, 287.3) |
| **South Asia** | 941.2 (372.8, 1908.6) | 5.4 (1.8, 11.7) | 6404.8 (2657.2, 13387.2) | 5.6 (1.7, 13.1) | 537041.7 (327662.8, 861790.3) | 166.3 (92, 290.5) | 246132.9 (154642.9, 385942.4) | 124.9 (66.2, 222.2) |
| **Southeast Asia** | 85649.2 (34745.7, 169585.9) | 35 (12.7, 70.8) | 3595.3 (1280.2, 7797.6) | 6.9 (2.5, 15.1) | 42896.3 (25121.7, 72581.9) | 136.1 (72.3, 232.3) | 39288.9 (21875.9, 66946.5) | 69.6 (37.1, 122.4) |
| **Southern Latin America** | 968.5 (444.5, 1912.5) | 6.8 (2.1, 15.5) | 8364.8 (4162.9, 15191.3) | 14.5 (5.6, 30) | 119292.2 (72038.1, 191804.1) | 277.1 (159.3, 469.3) | 1254856.4 (829219.6, 1862370.6) | 274.7 (160.6, 448.1) |
| **Southern Sub-Saharan Africa** | 22524.1 (9247.7, 43887.4) | 6.3 (1.9, 14.2) | 8407.9 (2708.4, 18340.2) | 3.8 (1.2, 8.4) | 478683.8 (314359.9, 727145.9) | 103.2 (51.7, 191.2) | 67594.2 (40216.9, 110749) | 132.4 (62.7, 251) |
| **Tropical Latin America** | 2401 (998.5, 4945.7) | 4.7 (1.4, 10.8) | 2351.4 (988.9, 4782.7) | 6.7 (2, 15.6) | 76253.3 (45717.4, 126921.9) | 162.8 (78.7, 289.5) | 12315.2 (7985.4, 18765.9) | 161.2 (91.7, 274) |
| **Western Europe** | 1480.8 (600, 3126.9) | 6.8 (2.1, 15.4) | 56070.1 (23080, 114094.2) | 5.1 (1.7, 11.2) | 278603 (175912.1, 442085.1) | 313.7 (174.9, 543.8) | 72806.6 (41487, 124399.5) | 141.6 (80.7, 241.8) |
| **Western Sub-Saharan Africa** | 4135.9 (1829, 8034.1) | 6.4 (2.2, 13.8) | 1822.9 (601.5, 4070.9) | 3.5 (1.1, 7.8) | 620661.7 (354482.5, 1058726.8) | 118.5 (54.9, 218.3) | 316276.5 (172349.1, 559563.2) | 133.4 (69.2, 240.9) |

Abbreviations: DALY, disability adjusted life year; ASR, age-standardized rate; UI, uncertainty interval.
